# The Malignant Transformation of Viral Hepatitis to Hepatocellular Carcinoma: Mechanisms and Interventions

**DOI:** 10.1002/mco2.70121

**Published:** 2025-03-08

**Authors:** Huimin Yuan, Ruochen Xu, Senlin Li, Mengzhu Zheng, Qingyi Tong, Ming Xiang, Yonghui Zhang

**Affiliations:** ^1^ Department of Pharmacology School of Pharmacy Tongji Medical College Huazhong University of Science and Technology Wuhan Hubei China; ^2^ Hubei Key Laboratory of Natural Medicinal Chemistry and Resource Evaluation School of Pharmacy Tongji Medical College Huazhong University of Science and Technology Wuhan Hubei China

**Keywords:** hepatocellular carcinoma, molecular mechanism, malignant transformation, therapeutic intervention, viral hepatitis

## Abstract

Hepatocellular carcinoma (HCC) is a leading cause of cancer‐related mortality globally, predominantly associated with chronic hepatitis B virus (HBV) and hepatitis C virus (HCV) infections. These infections drive persistent liver inflammation, culminating in cellular dysregulation, fibrosis, and cancer. Despite advancements in targeted therapies, drug resistance and the lack of reliable biomarkers for patient stratification still terribly hinder the treatment of viral HCC. To this end, the review delves into the intricate mechanisms underlying the malignant transformation of viral hepatitis to HCC, including viral integration, genomic instability, epigenetic modifications, oxidative stress, gut microbiota dysbiosis, chronic inflammation, immune escape, and abnormal signaling pathways, highlighting their complex interactions and synergies. Cutting‐edge preclinical and clinical advancements in HCC management, including lifestyle modifications, drug therapies, immunotherapies, gene‐based approaches, and innovative treatments, are further investigated, with particular priority given to their therapeutic potential and future applications in overcoming current limitations. By synthesizing recent scientific and clinical insights, this review aims to deepen the understanding of HCC pathogenesis in the context of chronic viral hepatitis, paving the way for novel therapeutic targets and personalized treatment strategies, ultimately improving patient outcomes.

## Graphical abstract

The malignant transformation from chronic viral hepatitis to hepatocellular carcinoma (HCC) involves intricate interactions among viral, host, and environmental factors. Key drivers of this transformation include viral integration, genomic instability, epigenetic modifications, oxidative stress, gut microbiota dysbiosis, chronic inflammation, immune evasion, and abnormal signaling pathways. Intervention strategies range from lifestyle modifications and drug therapies to immunotherapies, gene‐based approaches, and innovative treatments.

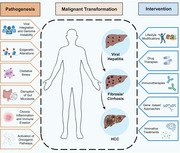

## 1 Introduction

Hepatocellular carcinoma (HCC) is the most common primary liver cancer and a leading cause of cancer‐related deaths worldwide, comprising nearly 90% of liver cancer cases [1, 2]. Challenges in its early diagnosis and the lack of specific treatments for advanced stages contribute to its aggressive nature and poor prognosis [3]. Chronic viral hepatitis, particularly infections with hepatitis B virus (HBV) and hepatitis C virus (HCV), is a major risk factor for HCC, as both cause long‐term liver damage that leads to cirrhosis and cancer [4]. Both viruses induce sustained liver inflammation, oxidative stress, and continuous hepatocyte regeneration, driving a progressive cycle of liver damage, fibrosis, and carcinogenesis [5, 6].

The molecular mechanisms of HCC development differ between HBV and HCV, highlighting their distinct oncogenic properties. The integration of HBV into the host genome significantly contributes to liver cancer development by inducing chromosomal instability, mutations, and the deregulation of oncogenes and tumor suppressor genes [7]. HBV also encodes viral proteins like HBV X protein (HBx), which directly promote tumorigenesis by altering cellular signaling pathways involved in cell proliferation, apoptosis, and DNA repair [8]. In chronic HBV infection, viral replication coexists with the host immune response, creating chronic liver inflammation that exacerbates DNA damage and supports malignant hepatocyte transformation [9]. Contrasting with HBV, HCV exerts its oncogenic effects mainly through indirect mechanisms, including chronic inflammation, oxidative stress, and steatosis (fat accumulation in the liver), leading to cirrhosis and HCC. HCV proteins, particularly core protein, nonstructural protein 3 (NS3), and nonstructural protein 5A (NS5A), are involved in dysregulating key cellular pathways, including Wingless‐int (Wnt)/β‐catenin, p53, and phosphoinositide 3‐kinase/protein kinase B (PI3K/AKT) signaling cascades, which disrupt normal cell growth, apoptosis, and immune surveillance, thereby forming a carcinogenic environment [10, 11]. Despite the well‐established link between chronic viral hepatitis and HCC, the molecular mechanisms governing the transition from chronic liver disease (CLD) to malignancy remain complex and not fully understood. Understanding the molecular drivers of hepatocarcinogenesis is crucial for developing strategies to prevent and treat HCC, especially in high‐risk populations with chronic HBV or HCV infection.

The strong association between viral hepatitis and HCC makes antiviral therapy necessarily important in reducing HCC risk in patients with chronic HBV or HCV infection. Nucleoside analogues (NAs) and interferon (IFN) therapy, which suppress HBV replication, have significantly reduced HCC incidence in chronic HBV patients [12, 13]. Similarly, direct‐acting antivirals (DAAs) for HCV have revolutionized chronic HCV treatment, achieving sustained virological response (SVR) in most patients and lowering HCC risk [14, 15]. Despite successful antiviral treatment, HCC risk persists, particularly in patients with advanced fibrosis or cirrhosis [15, 16]. Therefore, ongoing HCC surveillance is essential, and there is an urgent need for new therapy targeting the molecular pathways from viral hepatitis to liver cancer. As immunotherapies and targeted therapies emerge as promising options for advanced HCC, deeper insights into the molecular mechanisms of virus‐induced HCC are crucial for improving patient outcomes and advancing personalized medicine.

Herein, we first summarize the epidemiology of HBV‐ and HCV‐related HCC, followed by an exploration of the cellular and molecular mechanisms of malignant transformation, with a focus on important signaling pathways. Subsequently, we assess current treatments, including emerging therapies, and conclude by discussing future research directions, further emphasizing the need for innovative, multitargeted approaches for effective management. By integrating the latest research, we aim to bridge academic studies and clinical practice, deepen understanding of HCC progression from viral hepatitis, and foster the discovery of novel therapeutic targets and strategies to combat the disease.

## 2 Overview of Viral Life Cycles

HBV, a member of the Hepadnaviridae family, has a double‐layered envelope that surrounds a core particle. The envelope contains the surface antigen (S antigen) along with pre‐S1 and pre‐S2 antigens, which form three types of protein structures on the envelope, collectively known as the hepatitis B surface antigen (HBsAg) [17]. The core particle, which constitutes the viral capsid, is made of HBV core antigen (HBcAg). Inside the core particle is circular, partially double‐stranded DNA and polymerase, an enzyme essential for HBV genome replication [18]. The soluble antigen hepatitis B e antigen (HBeAg) is associated with the nucleocapsid and present in the serum [19]. The HBV genome consists of partially double‐stranded relaxed circular DNA (rcDNA) with four overlapping open reading frames (ORFs): ORF‐P, ‐S, ‐C, and ‐X, that also share overlapping regulatory sequences. ORF‐P encodes DNA polymerase, reverse transcriptase, and a terminal protein with priming enzyme activity [20]. ORF‐S includes pre‐S1, pre‐S2, and S genes, encoding HBsAg proteins: pre‐S1 protein, pre‐S2 protein, and S protein. ORF‐C contains pre‐C and C genes, encoding HBcAg and HBeAg, respectively [21]. Last, ORF‐X encodes the X protein, the smallest HBV protein, which regulates transcription and activates enhancers and promoters of homologous and heterologous genes. The X protein is also closely linked to HBV infection and the development of HCC (Figure S1A,C) [22].

The pivotal step in the HBV lifecycle is the binding of HBV virus to the host receptor taurocholic acid sodium cotransporter polypeptide 1 (NTCP1), after which the viral nucleocapsid releases its DNA, transforming it into covalently closed circular DNA (cccDNA) [23]. HBV cccDNA is crucial for persistent infection, combining with histone and nonhistone proteins to form viral microchromosomes, which serve as templates for viral RNA transcription [24]. In long‐term infections, HBV DNA may integrate into the host genome, leading to genomic instability in hepatocytes, a notable factor in the development of HCC [25, 26]. Viral RNA is transcribed from cccDNA and exported to the cytoplasm, where it is translated into viral proteins [27]. Furthermore, the pregenomic RNA (pgRNA) is packaged by core protein to form the viral nucleocapsid [28]. PgRNA is transported from the nucleus to the cytoplasm, reverse transcribed to form a rcDNA negative strand, which is then used as a template to synthesize a positive rcDNA strand, while viral DNA‐containing nucleocapsids are enveloped in the endoplasmic reticulum (ER) and secreted as mature virions [21]. During replication, some nucleocapsids may contain double‐stranded linear DNA (dslDNA) instead of rcDNA. These dslDNA‐containing virions, by‐products of pgRNA reverse transcription, may be released as defective virions, but they can still infect neighboring cells and integrate into the host genome at double‐stranded DNA breaks (Figure S1E) [29].

HCV, a member of the Flaviviridae family, primarily infects hepatocytes, with its outer layer consisting of envelope glycoproteins and its inner layer containing the nucleocapsid [30]. The nucleocapsid houses the HCV genome, a single‐stranded sense RNA approximately 9600 nucleotides long, which forms a core particle with a diameter of 30–35 nm and is wrapped to create a complete HCV particle [31]. In addition, a characteristic of HCV genes is the presence of 5′ and 3′ untranslated regions (UTRs) on both sides of the ORF, where ribosomes bind to the internal ribosome entry site (IRES) in the 5' UTR to translate the genome into a polyprotein precursor [32]. The polyprotein is cleaved by both host and viral proteases into functional HCV proteins, including the structural core protein and glycoproteins E1 and E2, as well as nonstructural proteins such as p7, nonstructural protein 2 (NS2), NS3, nonstructural protein 4A (NS4A), NS5A, and nonstructural protein 5B (NS5B), all of which are essential for the virus life cycle (Figure S1B,D) [33, 34].

The HCV life cycle involves several stages: attachment, endocytosis, fusion, genome release, RNA translation, RNA replication, viral assembly, maturation, and release [35]. Infection begins when HCV binds to host cells, primarily via two receptors: low‐density lipoprotein receptor (LDLR) and heparan sulfate proteoglycan (HSPG), which enhance the binding of the HCV envelope E1/E2 heterodimer to additional host receptors, such as CD81 and scavenger receptor B1 (SR‐B1) [36]. Once bound, HCV particles are internalized into endosomes, where acidic conditions trigger the release of viral RNA into cytoplasm through membrane fusion. Inside the cell, the viral RNA binds to ribosomes on the rough ER and serves as a template for translation into a polyprotein precursor [37]. The precursor protein, approximately 3000 amino acids long, is subsequently cleaved by viral proteases into 10 mature viral proteins, including both structural and nonstructural proteins [38]. Simultaneously, under the action of RNA‐dependent RNA polymerase (NS5B), negative‐stranded RNA intermediates are synthesized as templates for new HCV RNA replication. Subsequently, the core protein binds with viral RNA to form a nucleocapsid, recruits lipid droplets from the host cell for assembly, processes them in the Golgi apparatus, and transports them to the cell surface via multivesicular transport. Finally, mature viruses are released from the host cell through exocytosis, completing the virus lifecycle (Figure S1F) [39].

## 3 Epidemiology of HCC from Infection by Hepatitis Virus

The geographical variability in HCC incidence and its heterogeneity is closely tied to the global distribution of HBV and HCV infections. According to the World Health Organization (WHO), HBV‐related liver disease accounts for 30–50% of HCC cases, while HCV is responsible for roughly 25% of cases globally [1, 40]. The geographic distribution of HCC largely reflects the prevalence of chronic HBV and HCV infections due to the direct oncogenic role of these viruses.

HBV infection is a major global health concern, affecting approximately 2 billion people, with over 350 million chronic carriers [41]. In the world, the highest infection rates are in Africa and the Western Pacific, at 5.8 and 5.0%, respectively, whereas the Americas have a much lower rate of 0.5% [42]. Chronic HBV infection is a major cause of liver cancer and related deaths worldwide, contributing to about 50% of liver cancer deaths in 2020. Individuals with untreated chronic HBV infection face a risk of developing HCC that is 5 to 100 times greater than the general population [43]. A systematic review estimated HBsAg‐positive individuals have a 15‐ to 20‐fold higher relative risk (RR) of developing HCC compared with HBsAg‐negative individuals [44]. Additionally, studies from Asia have shown that HBV genotype C is associated with more severe liver disease, including cirrhosis and HCC, compared with genotype B, while genotype D is linked to a higher incidence of HCC than genotype A in Western Europe and North America [45, 46]. HBV can be transmitted through vertical transmission (from mother to child at birth) or horizontal transmission (through direct blood contact or sexual contact). In regions with high endemicity, vertical transmission is the most common, whereas in low‐endemic regions, transmission typically occurs through unprotected sexual contact, injection drug use, or occupational exposure [47]. Despite the introduction of HBV vaccination programs in the 1990s, which have significantly reduced new infections, HCC remains a major health concern due to the large population of chronically infected individuals.

Importantly, HBV‐infected individuals can develop HCC even without cirrhosis or significant liver fibrosis [48]. Serum HBV DNA levels are strong predictors of HCC development, independent of HBeAg status, cirrhosis, or alanine aminotransferase (ALT) levels. Additional risk factors for HCC include a family history of liver cancer, male gender, older age, coinfections with HCV, hepatitis D virus (HDV), or human immunodeficiency virus (HIV), and environmental factors such as aflatoxin exposure, heavy alcohol use, and smoking [49, 50]. Official guidelines recommend regular liver ultrasound monitoring for Asian men over 40 years of age, women over 50 years of age, and individuals with a family history of HCC, even if they do not have cirrhosis [51].

The global HCV infection rate is approximately 1.6%, affecting around 115 million people, with 71 million experiencing active viremia [52]. HCV geographical distribution is uneven, with infection rates exceeding 3.5% in Central Asia, East Asia, North Africa, and the Middle East, while Western countries have relatively low infection rates (<1%) [42, 53]. Over the past few decades, mortality from HCV‐related cirrhosis and HCC has risen significantly, reaching 500 thousand annual deaths by the 2020s. Epidemiological studies have demonstrated a strong association between HCV infection and HCC incidence, with chronic infection raising the risk of HCC by 15–30 times compared with HCV‐negative individuals, especially in high‐prevalence regions like Japan [54]. HCV is highly heterogeneous, with seven recognized genotypes that differ by 30–35% of their nucleotide sequences. The geographical distribution of HCV genotypes is complex, with “epidemic subtypes” like 1a, 1b, 2a, and 3a being widespread and accounting for a large proportion of cases, especially in high‐income countries. In contrast, “endemic” strains are rarer and found in specific regions, such as West Africa, South Asia, Central Africa, and Southeast Asia [53, 55]. HCV is primarily transmitted through blood‐to‐blood contact, most commonly via injection drug use, and to a lesser extent, through transfusion of contaminated blood products. Unlike HBV, sexual transmission of HCV is relatively rare, except in certain high‐risk groups, such as men who have sex with men who are coinfected with HIV [56].

Several factors predict the progression of HCV‐related HCC, including host factors such as older age, longer infection duration, male gender, and alcohol consumption exceeding 50 g per day, as well as viral factors like genotype, subtype, and viral load. Official guidelines classify patients into “ultra‐high‐risk” (those with hepatitis C cirrhosis) and “high‐risk” categories, recommending surveillance every 6 months [51, 57].

Epidemiological variability is closely linked to differences in healthcare infrastructure, vaccination programs, and access to medical resources across regions [1]. Thanks to the widespread use of the HBV vaccine, HBV infection rates have significantly decreased in many countries, making it one of the most effective measures for preventing HCC. However, there is currently no effective vaccine for HCV, making antiviral treatment the primary method of controlling HCV infection. In conclusion, despite ongoing advancements in prevention and treatment, the incidence and mortality of hepatitis‐related HCC remain high worldwide.

## 4 Pathogenesis of the Malignant Transformation of Viral Hepatitis to HCC

**FIGURE 1:**
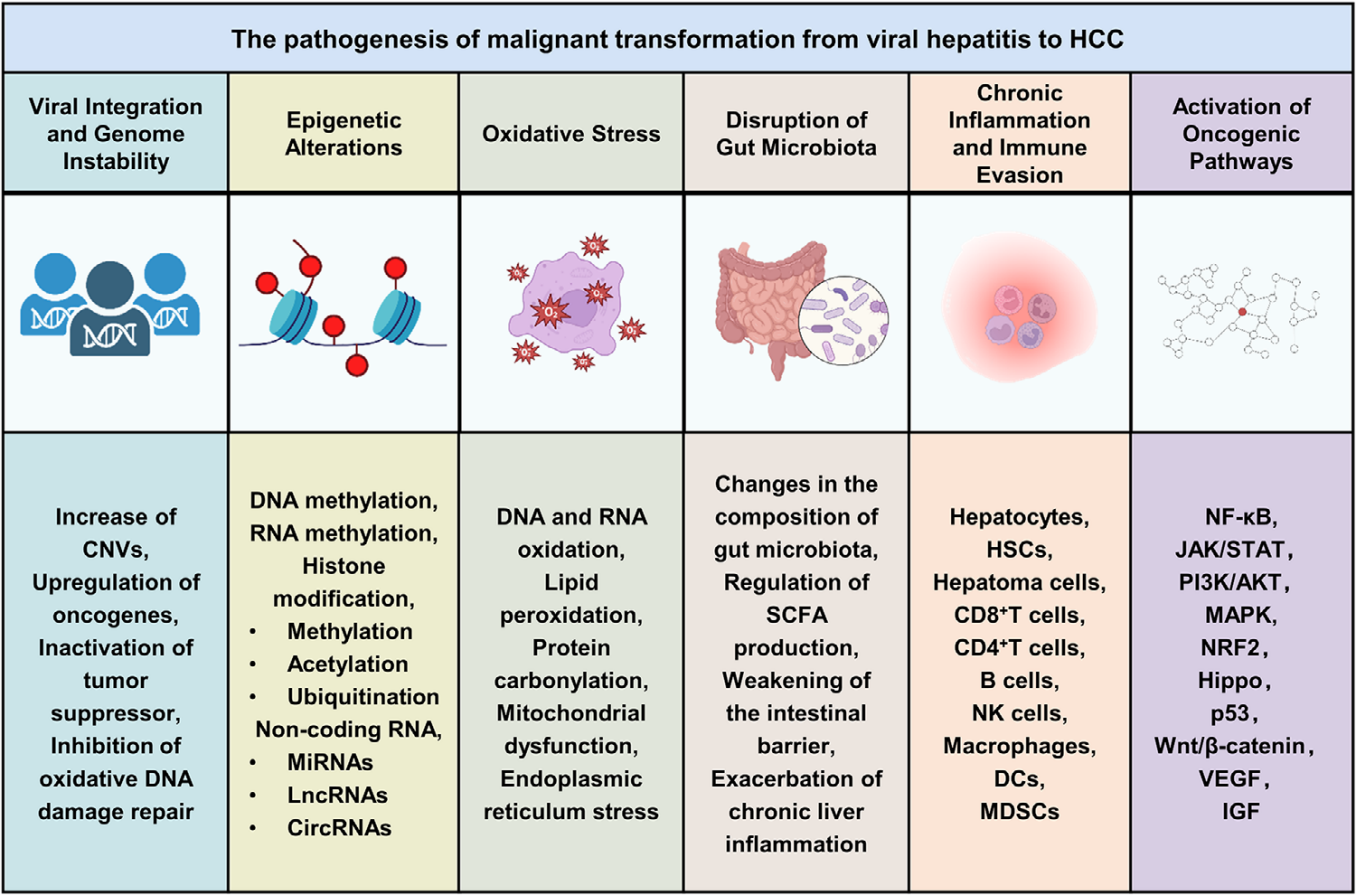
Mechanisms of the malignant transformation from viral hepatitis to HCC. Key mechanisms include viral integration and genome instability, epigenetic changes, oxidative stress, gut microbiota disruption, chronic inflammation and immune evasion, and oncogenic pathway activation. CNVs, copy number variations; DCs, dendritic cells; HBV, hepatitis B virus; HCV, hepatitis C virus; HCC, hepatocellular carcinoma; HSCs, hepatic stellate cells; IGF, insulin like growth factor; JAK/STAT, Janus tyrosine kinase/signal transducers and activators of transcription; MAPK, mitogen‐activated protein kinase; MDSCs, myeloid‐derived suppressor cells; NF‐κB, nuclear factor kappa B; NK cells, natural killer cells; NRF2, nuclear factor erythroid 2‐related factor 2; PI3K/AKT, phosphoinositide 3‐kinase/protein kinase B; SCFA, short‐chain fatty acid; VEGF, vascular endothelial growth factor.

Viral hepatitis, particularly caused by HBV and HCV, is a major driver of HCC. The transition from chronic hepatitis to malignancy involves multiple mechanisms, including viral integration and genome instability, epigenetic alterations, oxidative stress, disruption of gut microbiota, chronic inflammation, immune evasion, and activation of oncogenic pathways. Over time, these viral effects, combined with immune‐mediated liver injury, create a tumor‐prone microenvironment that induces the malignant transformation of hepatocytes (Figure 1).

### 4.1 Viral Integration and Genome Instability

Genomic instability plays a central role in the development of HCC. Chronic infections with HBV and HCV induce genomic instability in liver cells, initiating liver carcinogenesis and advancing the progression of HCC.

A major cause of genomic instability is the integration of HBV DNA into the host genome. In HBV‐infected liver tissues, the virus integrates into the chromosomal regions, often forming extrachromosomal circular DNA in unstable chromosomal regions, cytosine–phosphate–guanine dinucleotide (CpG) islands, and near telomeres, resulting in abnormal chromosomal alterations [58, 59]. Whole‐genome sequencing has shown that HBV integration sites are recurrent in tumor tissues, observed in 80% of HBV–HCC patients. The integration frequently causes copy number variations (CNVs) in adjacent chromosomes, further destabilizing the genome and triggering mutations, chromosomal deletions, and gene rearrangements [60]. These events not only disrupt chromosome structure but also accelerate tumor progression by upregulating genes near the integration sites [61]. Genes such as telomerase reverse transcriptase (TERT), mixed lineage leukemia 2 (MLL2), myelocytomatosis (MYC), and AT‐rich interaction domain 1A (ARID1A), frequent targets of HBV integration, are strongly associated with cell proliferation, chromatin regulation, and DNA repair [7, 62]. Abnormal gene expression due to HBV integration plays a pivotal role in tumorigenesis. TERT overexpression promotes tumor cell immortalization, driving malignant transformation and ultimately leading to HCC [63]. Additionally, MLL family genes, often affected by HBV integration, are involved in chromatin remodeling and gene regulation, and their mutations or dysregulation can further accelerate tumor progression. HBV integration also inactivates tumor suppressor genes, with the HBx protein inhibiting tumor suppressor genes such as p53 and retinoblastoma protein (RB) by interacting with various transcription factors, disrupting apoptosis, and promoting abnormal cell proliferation [64, 65].

Moreover, HBV integration generates mutant viral proteins that contribute to HCC development. Mutated or truncated forms of HBsAg, HBcAg, and HBx proteins, resulting from integration, can trigger stress responses in the ER and mitochondria, inducing apoptosis, inflammation, and impairments in DNA damage repair pathways [66]. Particularly, truncated HBx proteins form chimeric virus–host transcripts, typically containing C‐terminally truncated HBx proteins, which enhance cancer cell proliferation and migration by activating the Wnt‐5a signaling pathway and inhibiting apoptosis, thereby exacerbating malignant transformation through oxidative DNA damage and increased matrix metalloproteinase 10 (MMP‐10) expression [67]. Moreover, they impair the DNA damage repair response by interacting with DNA binding protein 1 (DDB1), exacerbating genomic instability and heightening cancer risk [68].

In contrast to HBV, HCV does not integrate into the host genome but induces significant genomic instability via other mechanisms. HCV infection induces chronic inflammation, characterized by immune cell recruitment and the release of proinflammatory cytokines. Persistent inflammation leads to oxidative stress and the generation of reactive oxygen species (ROS), damaging DNA, proteins, and lipids, and ultimately causing mutations and genomic instability [69]. HCV proteins, particularly core protein, NS3, and NS5A, disrupt cellular pathways responsible for maintaining genome integrity. HCV core protein inhibits the repair of oxidative DNA damage by suppressing DNA glycosylases that repair 8‐hydroxy‐2'‐deoxyguanosine (8‐OHdG) [70]. Additionally, HCV proteins interact with various DNA repair factors, hindering the repair of oxidative damage, which leads to increased levels of 8‐OHdG and single‐strand breaks in infected individuals [71]. HCV infection also induces elevated TERT expression and decreased p53 expression [72, 73]. Additionally, chronic HCV infection is linked to an increasing proportion of mitochondrial DNA (mtDNA) loss in patients over time [74].

In conclusion, genomic instability induced by both HBV and HCV plays a critical role in the development of HCC. HBV‐induced genomic instability, oncogene activation, tumor suppressor gene inactivation, and the production of mutant viral proteins collectively drive the malignant transformation of liver cells. Although HCV does not integrate into the host genome, its persistent replication and associated inflammation create an environment conducive to genetic changes, ultimately leading to HCC.

### 4.2 Epigenetic Alterations

Epigenetic alterations play a pivotal role in the malignant transformation of hepatocytes during chronic viral hepatitis by modulating gene expression without changing the underlying DNA sequence, thereby impacting processes such as cell proliferation, apoptosis, and inflammation, which are momentous factors in HCC development [75]. Hepatitis viruses regulate DNA methylation, RNA methylation, histone modifications, and noncoding RNA (ncRNA) expression, driving the malignant progression from chronic hepatitis to HCC.

#### 4.2.1 DNA Methylation

DNA methylation refers to the addition of a methyl group to cytosine residues, especially in CpG islands, which inhibits gene expression [76]. It plays a meaningful role in silencing tumor suppressor genes and in the early stages of progression from viral hepatitis to HCC.

HBV infection induces abnormal DNA methylation, primarily through the action of the HBx protein. The HBx protein upregulates DNA methyltransferase 1 (DNMT1), causing hypermethylation and inactivation of several tumor suppressor genes [77]. Studies have shown that Ras association domain family 1 isoform A (RASSF1A), a tumor suppressor involved in cell cycle regulation, is significantly hypermethylated in the livers of over 50% of HBV‐infected individuals, a pattern seen early in HCC [78]. The p16 (INK4A), a cell cycle inhibitor, also undergoes hypermethylation in HBV‐infected hepatocytes, disrupting cell cycle regulation and promoting tumor development [79]. E‐cadherin (CDH1) methylation is another critical event in HBV‐related HCC. E‐cadherin is vital for epithelial–mesenchymal transition (EMT), and its silencing via hypermethylation increases tumor cell invasiveness [80]. G Protein Subunit Alpha 14 (GNA14) expression is significantly downregulated in HBV‐related HCC, with DNA methylation being the primary cause. HBx protein regulates GNA14 methylation, affecting the notch homologue protein 1 (Notch1) signaling pathway, a key regulator of tumor proliferation and metastasis [81].

HCV does not integrate into the host genome like HBV, but it promotes liver cancer through epigenetic mechanisms. HCV infection upregulates DNMT1, inducing hypermethylation and silencing of several tumor suppressor genes, including suppressor of cytokine signaling‐1 (SOCS‐1), RASSF1A, and GSH S‐transferase P1 (GSTP1) [82, 83]. SOCS‐1, a negative regulator of the Janus kinase/signal transducer and activator of transcription (JAK/STAT) pathway, has strong antitumor activity in HCC progression [84]. In HCV‐infected HCC patients, SOCS‐1 methylation is significantly elevated, indicating that HCV suppresses SOCS‐1 expression through methylation, promoting liver malignancy [85]. Furthermore, HCV core protein overexpression inhibits STAT1 activation via methylation, interfering with IFN‐α signaling and hindering the antiviral response, facilitating viral replication [86]. The transcription factor cyclic AMP‐responsive element‐binding protein 3‐like protein 1 (CREB3L1) is silenced by methylation in HCV‐infected cells, impacting antiviral gene expression and potentially contributing to treatment resistance [86].

DNA methylation not only plays a crucial role in viral hepatitis infections but also dominates the early stages of HCC. Tumor suppressor gene hypermethylation is a hallmark of early HCC development. In early HCC, DNA methylation inhibits the binding of p53 to the tumor suppressor Zinc‐finger protein 334 (ZNF334), thereby reducing p53 expression, further emphasizing the importance of DNA methylation in the pathogenesis of HCC [87]. Secreted frizzled‐related protein 2 (SFRP2), another potential tumor suppressor, frequently shows promoter hypermethylation in HBV‐related HCC. SFRP2 methylation levels exceed normal and have higher diagnostic value than alpha‐fetoprotein (AFP), indicating its potential for early HCC diagnosis [88].

In contrast to localized hypermethylation, HBV and HCV infections also trigger global hypomethylation. Genome‐wide hypomethylation causes chromosomal instability, increasing gene mutation and rearrangement rates, thus promoting HCC malignancy [89]. HBx protein downregulates DNMT3B, inducing genome‐wide hypomethylation, leading to genomic instability and driving tumor progression [78]. The combined effects of localized tumor suppressor hypermethylation and genome‐wide hypomethylation caused by viral hepatitis drive the development and progression of HCC.

#### 4.2.2 RNA Methylation

RNA methylation refers to the process of adding methyl groups to RNA molecules, where methyl groups are transferred from methyl donors to RNA bases under the catalysis of RNA methyltransferases [90]. Hepatitis viruses promote HCC progression by regulating RNA methylation, especially N^6^‐methyladenosine (m6A). Recent studies have highlighted that m6A modifications are crucial for viral replication, immune evasion, and oncogenesis.

In HBV‐related HCC, m6A modifications drive tumor initiation and progression by regulating prominent genes. Methyltransferase‐like 3 (METTL3), a core component of the m6A methyltransferase complex, is pivotal in m6A modification after HBV infection [91]. HBV infection induces m6A modification in the 3′‐UTR of phosphatase and tensin homolog (PTEN) mRNA by upregulating METTL3, reducing its stability, and activating the PI3K/AKT pathway, thus promoting HCC development [92]. HBV also influences m6A modification via its HBx protein. HBx, an important regulator of viral transcription, promotes m6A modification of viral RNA and induces METTL3 nuclear localization, facilitating m6A addition to viral and host RNA. HBx interacts with the METTL3/14 complex to promote m6A modification of host genes, accelerating tumor progression [93]. YTH domain family 2 (YTHDF2), an “m6A reader,” is another important regulatory protein in HBV‐related HCC. Studies have shown that YTHDF2 undergoes O‐GlcNAcylation after HBV infection, increasing its stability and oncogenic activity, which promotes the cell cycle by stabilizing m6A‐modified transcripts like minichromosome maintenance 2 (MCM2) and MCM5, further driving HBV‐related HCC [94]. The m6A demethylase AlkB homolog 5 (ALKBH5) also contributes to HBV‐related HCC. ALKBH5 overexpression removes m6A from HBx mRNA, increasing its stability and promoting tumor cell proliferation and migration. Knocking down ALKBH5 inhibits HBV‐related HCC progression, indicating it as a potential therapeutic target [95].

Like HBV, HCV also utilizes m6A modification to promote HCC development. M6A modifications are prevalent in the RNA genome of HCV and regulate its life cycle and impact on host cells [96]. Studies have shown that m6A‐modified HCV RNA is recognized by the YTHDF protein family, which regulates HCV translation and RNA stability [97]. YTHDC2 recognizes m6A modifications on HCV RNA to promote IRES‐dependent translation initiation, while YTHDF2 binds to the 3′‐UTR of HCV RNA, inhibiting retinoic acid‐inducible gene I (RIG‐I)‐mediated immune recognition and facilitating immune evasion [98]. HCV also drives liver cancer by modulating m6A modifications in host cells. HCV infection increases m6A levels in genes like RIO kinase 3 (RIOK3) and cold‐inducible RNA‐binding protein (CIRBP), enhancing their translation, promoting immune evasion, and boosting tumor cell proliferation [99].

In summary, m6A modifications are vital in HBV‐ and HCV‐related HCC transformation through various mechanisms. HBV and HCV exploit m6A regulation of viral RNA and host genes to evade immune surveillance while promoting tumor proliferation and metastasis.

#### 4.2.3 Histone Modifications

Histone modification refers to the process in which histones undergo methylation, acetylation, ubiquitination, and phosphorylation, catalyzed by specific enzymes, to regulate cellular processes [100]. Hepatitis viruses manipulate chromatin structure by altering histone modifications, influencing host–cell transcription and potentially leading to tumorigenesis.

##### 4.2.3.1 Methylation

Histone methylation, a common posttranslational modification on the N‐terminal tails of histones H3 and H4, is catalyzed by histone methyltransferases (HMTs), with histone demethylases removing these modifications [101]. Both HBV and HCV promote hepatocyte malignant transformation and HCC progression by regulating histone methylation.

In HBV infection, the viral HBx protein significantly alters histone methylation, activating liver cancer‐related genes. In HBV‐related HCC, HBx interacts with various chromatin regulatory factors to modify chromatin structure, promoting oncogene expression. Studies have indicated that HBx binds to WD repeat domain 5 (WDR5), enhancing the trimethylation of lysine 4 on histone H3 (H3K4me3), an epigenetic marker associated with gene activation. HBx activates multiple tumor‐related genes, including ALKBH5 and MMP, through WDR5‐driven modification [102]. Inhibiting WDR5 with small‐molecule inhibitor WDR5‐0103 significantly suppresses HBV and HBx‐driven tumor growth, suggesting that WDR5 plays a critical role in HBV‐induced liver cancer [102]. Additionally, HBx colocalizes with WDR5 across genome‐wide chromatin regions, enhancing H3K4me3 modification and promoting HBV genome transcription and replication. The process depends on the interaction of HBx alpha‐helical domain with WDR5, which recruits WDR5 to gene promoters, activates gene expression, and drives malignant tumor progression [102].

Similarly, HCV infection influences HCC development through the regulation of histone methylation. It significantly alters histone modifications on cancer‐associated genes, particularly at lysine 27 (H3K27) and lysine 4 (H3K4) of histone H3. Specifically, HCV infection reduces H3K27 trimethylation while increasing H3K4 trimethylation, directly affecting genes involved in cell proliferation and tumor suppression, promoting abnormal cell growth [103]. Furthermore, the aberrant overexpression of vasohibin 2 (VASH2) in HCV‐related HCC is closely tied to increased H3K4 trimethylation, reinforcing the role of HCV in promoting malignant transformation through histone methylation [104]. Beyond methylation, demethylases also play a critical role in liver cancer development. Lysine‐specific demethylase 1 (LSD1), which removes methyl groups from H3K4 and H3K9, is upregulated in HCV‐related HCC and contributes to tumor suppressor gene silencing, promoting abnormal hepatocyte proliferation and malignant transformation [105]. Moreover, HCV stimulates the expression of protein phosphatase 2A (PP2A) through the ER stress pathway. PP2A interacts with protein arginine methyltransferase 1 (PRMT1) to inhibit its activity, thereby preventing PRMT1 from catalyzing the methylation of arginine 3 on histone H4, which regulates gene transcription and further disrupts epigenetic regulation, driving HCC progression [106, 107, 108].

In conclusion, both HBV and HCV significantly influence HCC malignant transformation by regulating histone methylation, which activates oncogene expression and suppresses essential tumor suppressor genes, thereby promoting hepatocyte malignant transformation.

##### 4.2.3.2 Acetylation

Histone acetylation is a frequent posttranslational modification in which an acetyl group from acetyl‐CoA is added to the lysine residues at the N‐terminus of histones [109]. The process is coregulated by histone acetyltransferases (HATs) and histone deacetylases (HDACs), which determine gene activation or silencing [110]. In HBV and HCV infections, abnormal histone acetylation can trigger hepatocyte malignant transformation, ultimately promoting HCC development.

During HBV infection, the core regulatory protein HBx interacts with epigenetic regulators, influencing histone acetylation and gene expression, thereby promoting hepatocyte carcinogenesis. Studies have indicated that HBx forms a complex with methyl‐CpG binding domain protein 2 (MBD2) and CREB‐binding protein (CBP)/p300, enhancing the acetylation of histones H3 and H4, which specifically alters the acetylation of histone H4 at the insulin‐like growth factor 2 (IGF‐2) promoter and activates IGF‐2 transcription [111, 112]. IGF‐2 is strongly linked to cell proliferation, and its overexpression directly drives abnormal hepatocyte growth, facilitating tumor initiation and progression [113]. In addition to upregulating histone acetylation, HDACs play a pivotal role in HBV‐related HCC by deacetylating histones, leading to tighter chromatin structure and suppression of essential tumor suppressor genes. HDACs suppress E‐cadherin expression, exacerbating EMT and enhancing tumor cell invasiveness. HDACs are widely upregulated in infected liver cells, further promoting hepatocyte malignant transformation [114, 115].

Likewise, HCV infection promotes HCC malignant transformation via histone acetylation regulation. For instance, HCV infection significantly alters H3K27 acetylation (H3K27ac), an epigenetic marker closely associated with gene activation, which increases the expression of various cancer‐related genes in the liver [116]. This modification activates proinflammatory signaling and influences important biological processes, including fatty acid metabolism, thus fostering a liver tumor microenvironment and accelerating cancer progression [117]. In a similar manner, H3K9 acetylation on histone H3 plays a key role in HCV‐related HCC. The HCV core protein modulates H3K9 acetylation, altering the expression of genes involved in cell proliferation and tumor progression [116, 118]. HDACs also play a crucial role in HCV‐related HCC. Research has shown that HDAC3 inhibitors can increase the expression of the hepcidin antimicrobial peptide (HAMP) in the liver and reduce apolipoprotein A1 (APOA1) levels, thus lowering HCV replication [119]. Additionally, HDAC3 inhibitors exhibit antiviral effects by altering histone acetylation levels of significant transcription factors such as hypoxia‐inducible factor‐1 alpha (HIF‐1α) and STAT3, leading to their evaluation in clinical treatment for HCC [119]. The HCV core protein inhibits the activity of HATs, such as p300 and CBP, reducing histone acetylation levels, which decreases the transcriptional activity of antiviral genes, facilitates viral persistence, and creates favorable conditions for cancer development [120].

Through the regulation of histone acetylation, HBV and HCV significantly influence the development and progression of HCC by increasing histone acetylation, which activates oncogene expression and suppresses key tumor suppressor genes, thereby promoting hepatocyte malignant transformation.

##### 4.2.3.3 Ubiquitination

Histone ubiquitination involves the covalent attachment of ubiquitin molecules to histones via enzymatic reactions, typically occurring at specific lysine residues, and plays a crucial role in regulating chromatin structure and gene expression [100]. During the transformation of chronic hepatitis to HCC, HBV and HCV exploit the ubiquitination system to manipulate the expression and stability of various proteins, thereby promoting tumor development and progression.

The conversion of healthy liver cells into tumor cells after HBV infection largely depends on the critical oncogenic protein HBx. HBx activates the ubiquitin–proteasome system (UPS), enhancing the oncogenic properties of cells by modulating the ubiquitination of various substrates. Studies have indicated that HBx, as an adaptor for ubiquitin E3 ligases, interacts with multiple E3 ligases to regulate substrate ubiquitination [121]. HBx inhibits the ubiquitination of E3 ligase ubiquitin‐like with PHD and ring finger domains 2 (UHRF2) while promoting its phosphorylation at serine 63, enhancing the invasiveness, migration, and proliferation of HBV‐infected cells [122]. Additionally, HBx interacts with nonmuscle myosin heavy chain (MYH9) to mediate the ubiquitination and degradation of glycogen synthase kinase 3β (GSK‐3β), activating the Wnt/β‐catenin pathway, which further promotes HCC progression and sorafenib resistance [123]. HBx also regulates the interaction between critical proteins, glucose‐regulated protein 78 (GRP78) and tripartite motif containing 25 (TRIM25) through ubiquitination, reducing TRIM25 ubiquitination levels, which increases SMAD family member 4 (SMAD4) activity and drives liver cancer progression [124]. Moreover, HBx upregulates E3 ligase MSL complex subunit 2 (MSL2) expression, promoting the ubiquitination and degradation of apolipoprotein B mRNA editing enzyme catalytic subunit 3B (APOBEC3B), thereby stabilizing HBV cccDNA, a critical factor in liver cancer development [125]. HBx also affects the degradation of cell cycle‐related proteins such as paired box 8 (PAX8) and pituitary tumor‐transforming gene 1 (PTTG1), disrupting normal cell cycle control and extending proliferation signals, promoting a tumor microenvironment [126]. Notably, HBx inhibits the ubiquitin‐dependent degradation of E12 and E47 proteins, delaying EMT, and enhancing the migration and invasiveness of liver cancer cells [127]. Additionally, HBx interacts with HIF‐1α, preventing its binding to von Hippel‐Lindau protein (pVHL), thereby inhibiting HIF‐1α ubiquitin‐mediated degradation, promoting angiogenesis and further driving HCC progression [128].

Analogously, HCV promotes HCC development through the regulation of ubiquitination. During HCV infection, the ubiquitination of important proteins is integral to viral replication and tumor progression. HCV NS5A protein, a core component of the viral replication complex, is subject to ubiquitination and degradation [129]. E3 ligase TRIM26 mediates the ubiquitination of the HCV NS5B protein, facilitating its interaction with NS5A, thereby boosting viral replication [130]. HCV NS2 protein is ubiquitinated by membrane‐associated RING‐CH 8 (MARCHF8) E3 ligase, which plays a key role in viral envelope assembly [131]. Additionally, research has shown that ubiquitin conjugating enzyme E2S (UBE2S) and Lys11‐linked chains are suppressed in HCV‐infected cells, leading to DNA damage and enhancing viral replication [132]. HCV infection also activates the ROS/JNK signaling pathway, promoting the activation of HECT‐type E3 ubiquitin transferase Itchy homolog (ITCH), which enhances HCV particle release through the polyubiquitination of vacuolar protein sortin 4 homolog A (VPS4A), thereby regulating viral release and spread, advancing the progression from chronic hepatitis to HCC [133]. Ubiquitination also regulates host proteins in the context of HCV infection. F‐box and WD repeat domain‐containing protein 7 (FBW7) recognizes HCV NS5B and mediates its K48‐linked ubiquitination, promoting NS5B degradation and inhibiting viral replication [134].

Beyond regulating viral protein stability, both HBV and HCV promote immune evasion and tumor progression by modulating the host immune response through ubiquitination. HBx enhances the ubiquitination of Beclin‐1, increasing autophagic flux and accelerating hepatocyte invasiveness [135]. HBV also regulates the ubiquitination of pVHL, inhibiting its degradation of HIF‐1α, promoting angiogenesis, and aiding tumor cells in evading immune surveillance [128]. During HCV infection, HCV‐E2 manipulates the activation of transcription factor Snail to induce fucosyltransferase 8 (FUT8) expression, which promotes TRIM40‐mediated RIG‐I K48 ubiquitination, inhibits the IFN response, and accelerates immune escape [136].

In summary, HBV and HCV critically influence the malignant transformation of HCC by regulating the stability and degradation of both viral and host proteins through the ubiquitination system. Ubiquitination not only affects the viral life cycle but also promotes liver cancer development by modulating the host immune response and cell proliferation.

#### 4.2.4 Noncoding RNA

NcRNAs are RNA molecules transcribed from the genome that do not encode proteins, playing critical roles in regulating gene expression and various cellular processes [137]. The abnormal expression of ncRNAs is closely associated with the metastasis, invasion, spread, and recurrence of virus‐associated HCC, such as that caused by HBV and HCV infections (Tables 1 and 2).

**TABLE 1:** NcRNAs in HBV‐related HCC.

Classification	Name	Expression	Target genes/proteins	Biological function	References
MiRNA	miR‐802	Up	SMARCE1	Promote HBV DNA replication and HbsAg/HbeAg expression	[138]
	miR‐203	Up	BANF1	Promote HBV replication	[139]
	miR‐302c‐3p	Down	BMPR2, HNF4A	Suppress HBV replication and HBsAg production	[140]
	miR‐29a	Up	SMARCE1	Promote HBV replication	[141]
	microRNA‐137	Down	NOTCH1	Suppress cell proliferation in HBV‐related HCC	[142]
	miR‐192‐5p	Up	–	Correlate with virological response	[143]
	miR‐3	Up	HBV‐RNA, SOCS‐5, PPM1A, PTEN	Suppress viral replication; promote cell proliferation in HBV‐related HCC	[144]
	miR‐23a	Down	CCL22	Inhibit Tregs recruitment	[145]
	miRNA‐30b‐5p	Up	MINPP1	Promote tumor growth, enhancing cell proliferation, promoting cell migration and invasion, regulating glycolytic bypass metabolism	[146]
	miR‐124	Down	PI3K/AKT	Suppress CSCs differentiation	[147]
	miR‐135a	Up	VAMP2	Prevent doxorubicin hydrochloride‐induced apoptosis	[148]
	miR‐200a/200b/429	Down	RICTOR	Impair HCC stem cell properties, regulating glutamine metabolism, sensitizing the response to anti‐PD‐L1 immunotherapy	[149]
	miRNA‐203a	Down	BMI1	Sensitize 5‐FU‐induced apoptosis, impairing HCC stem cell properties	[150]
	miR‐325‐3p	Down	DPAGT1	Sensitize the response to doxorubicin chemotherapy	[151]
	miR‐1236/miR‐329	Down	AFP	Sensitize chemotherapy‐induced apoptosis	[152]
	miR‐384	Down	PTN/PI3K/AKT/mTORC1	Inhibit high glucose‐induced lipogenesis	[153]
	miR‐3682‐3p	Up	FOXO3/PI3K/AKT/c‐Myc	Promote HCC stemness	[154]
	miR‐5188	Up	FOXO1/β‐catenin	Resist the effects of chemotherapy 5‐FU, CDDP, and EPI, promoting HCC stemness	[155]
LncRNA	HULC	Up	HBx/STAT3/miR‐539/APOBEC3B	Enhance the growth of HCC by activating HBV	[156]
	MALAT1	Up	PI3K/AKT	Promote HBx‐induced CSCs properties	[147]
	UCA1	Up	EZH2	Promote growth, metastasis, and EMT of HCC cell lines	[157]
	LINC00152	Up	EZH2	Promote the proliferation and EMT of HCC cell lines and tumourigenesis	[158]
	HOTTIP	Up	HOXA13	Suppress the generation of hepatitis B viral surface antigen, hepatitis B viral e antigen, and HBV replication	[159]
	ANRIL	Up	miR‐122‐5p	Inhibit apoptosis of HCC cells in vitro and promotes proliferation, invasion, and migration of HCC cells in vitro	[160]
	MAFG‐AS1	Up	E2F1, NM IIA	Promote proliferation and migration of HCC cells	[161]
	DBH‐AS1	Up	FAK/Src/ERK	Facilitate the tumorigenesis of HCC	[162]
	PVT1	Up	EZH2	Promote hepatitis B virus‑positive liver cancer progression	[163]
	DLEU2	Up	EZH2	Promote viral replication	[164]
	HOTAIR	Up	SP1	Promote viral replication	[165]
	LINC01152	Up	IL‐23, STAT3	Increase HCC cell proliferation and promotes tumor formation	[166]
	ZEB2‐AS1	Up	E‐cadherin, vimentin	Promote EMT induced by HBx	[167]
	PCNAP1	Up	miR‐154/PCNA/HBV cccDNA	Enhance HBV replication and hepatocarcinogenesis	[168]
	H19	Up	N‐cadherin, Vimentin, β‐catenin, MMP‐9	Promote malignant development of HBV‐related HCC	[169]
	LncRNA n335586	Up	miR‐924/CKMT1A	Promote HCC cells migration and invasion	[170]
	DREH	Down	Vimentin	Inhibit HBx‐mediated hepatocarcinogenesis	[171]
	SAMD12‐AS1	Up	NPM1	Promote cell proliferation and inhibits apoptosis	[172]
	HUR1	Up	p53	Promote cell proliferation and tumorigenesis	[173]
	lncRNA‐6195	Down	ENO1	Inhibit the energy metabolism in HCC cells	[174]
	SFMBT2	Up	–	Decrease the level of HBV DNA in human liver cancer cells	[175]
	AP000253	Up	–	Promote HBV transcription and replication in hepatoma cell lines	[176]
	LINC01010	Down	Vimentin filament	Suppress cell proliferation and migration	[177]
	MEG3	Down	α‐SMA, COL1A1	Serve as a serum biomarker for diagnosing CHB combined with liver fibrosis	[178]
	WEE2‐AS1	Up	FERMT3	Promote HBV infection and accelerate the proliferation, migration, invasion, and cell cycle progression of HCC cells	[179]
CircRNA	circBACH1	Up	MAP3K2	Promote HBV replication and hepatoma progression	[180]
	circRNA1002	Down	–	Serve as a reliable biomarker for HCC	[181]
	HBV_circ_1	Up	CDK1	Promote carcinogenesis and progress of HBV‐related HCC	[182]
	hsa_circ_0000650	Up	TGFβ2	Promote progression of CHB	[183]
	hsa_circ_0066966	Up	–	Promote the proliferation and migration of HBV‐positive liver cancer cells	[184]
	circ‐ARL3	Up	miR‐1305	Promote the proliferation and invasion of HBV HCC cells	[185]
	circ_0009582	Up	–	Serve as potential biomarker for predicting the occurrence of HCC in patients with HBV infection	[186]
	circ_0037120	Up	–	Serve as potential biomarker for predicting the occurrence of HCC in patients with HBV infection	[186]
	circ_0140117	Up	–	Serve as potential biomarker for predicting the occurrence of HCC in patients with HBV infection	[186]
	circ_101764	Up	hsa‐miR‐181	Promote the development of HBV‐related HCC	[187]
	circ_0004812	Up	miR‐1287‐5p	Promote HBV‐induced immune suppression	[188]
	circ_10156	Up	miR‐149‐3p	Promote HCC cell proliferation	[189]

Abbreviations: 5‐FU, 5‐fluorouracil; AFP, alpha‐fetoprotein; AKT, protein kinase B; ANRIL, antisense noncoding RNA in the INK4 locus; APOBEC3B, apolipoprotein B mRNA editing enzyme catalytic polypeptide 3B; ARL3, ADP‐ribosylation factor‐like protein 3; BACH1, BTB and CNC homology 1; BANF1, barriers to autointegration factor 1; BMI1, BMI1 proto‐oncogene, polycomb ring finger chromatin; BMPR2, bone morphogenetic protein receptor 2; cccDNA, covalently closed circular DNA; CCL22, C‐C motif chemokine ligand 22; CDDP, cis‐diamminedichloroplatinum(II); CDK1, cyclin‐dependent kinase 1; CHB, chronic hepatitis B; CircRNA, circular RNA; CKMT1A, creatine kinase mitochondrial 1A; c‐Myc, Myc proto‐oncogene protein; COL1A1, collagen type I alpha 1 chain; CSC, cancer stem cell; DBH‐AS1, dopamine beta‐hydroxylase antisense RNA 1; DLEU2, deleted in lymphocytic leukemia 2; DPAGT1, dolichyl‐phosphate N‐acetylglucosaminephosphotransferase 1; DREH, DNA replication element‐binding protein homolog; E2F1, E2F transcription factor 1; EMT, epithelial–mesenchymal transition; ENO1, enolase 1; EPI, epirubicin; ERK, extracellular signal‐regulated kinase; EZH2, enhancer of zeste homolog 2; FAK, focal adhesion kinase; FERMT3, fermitin family member 3; FOXO1, forkhead box O1; FOXO3, forkhead box O3; HbeAg, hepatitis B e antigen; HbsAg, hepatitis B surface antigen; HBV, hepatitis B virus; HBx, hepatitis B virus X protein; HCC, hepatocellular carcinoma; HNF4A, hepatocyte nuclear factor 4 alpha; HOTAIR, HOX transcript antisense RNA; HOTTIP, HOXA transcript at the distal tip; HOXA13, homeobox A13; HULC, highly upregulated in liver cancer; HUR1, heterogeneous nuclear ribonucleoprotein U; IL‐23, interleukin‐23; LncRNA, long noncoding RNA; MAFG‐AS1, MAF BZIP transcription factor G antisense RNA 1; MALAT1, metastasis‐associated lung adenocarcinoma transcript 1; MAP3K2, mitogen‐activated protein 3 kinase 2; MEG3, maternally expressed 3; MINPP1, multiple inositol polyphosphate phosphohydrolase 1; MiRNA, microRNA; MMP‐9, matrix metallopeptidase 9; mTORC1, mechanistic target of rapamycin complex 1; NM IIA, nonmuscle myosin IIA; NOTCH1, notch homologue protein 1; NPM1, nucleophosmin 1; PCNA, proliferating cell nuclear antigen; PCNAP1, proliferating cell nuclear antigen‐associated protein 1; PD‐L1, programmed death‐ligand 1; PI3K, phosphoinositol‐3 kinase; PPM1A, protein phosphatase, Mg^2+^/Mn^2+^ dependent 1A; PTEN, phosphatase and tensin homolog; PTN, pleiotrophin; PVT1, plasmacytoma variant translocation 1; RICTOR, rapamycin‐insensitive companion of mTOR; SFMBT2, scm‐like with four mbt domains 2; SMAD12‐AS1, SAMD12 antisense RNA 1; SMARCE1, SWI/SNF related, matrix associated, actin dependent regulator of chromatin, subfamily e, member 1; SOCS‐5, suppressor of cytokine signaling 5; SP1, specificity protein 1; Src, sarcoma viral oncogene homolog; STAT3, signal transducer and activator of transcription 3; TGFβ2, transforming growth factor beta 2; UCA1, urothelial cancer associated 1; VAMP2, vesicle‐associated membrane protein 2; WEE2‐AS1, WEE2 antisense RNA1; ZEB2‐AS1, zinc finger E‐box binding homeobox 2 antisense RNA 1; α‐SMA, alpha‐smooth muscle actin.

**TABLE 2:** NcRNAs in HCV‐related HCC.

Classification	Name	Expression	Target genes/proteins	Biological function	References
miRNA	miRNA‐182	Down	Claudin‐1	Suppress HCV replication	[190]
	miRNA‐122	Up	IRES	Facilitate HCV proliferation and suppress tumor formation	[191]
	miR‐200c	Down	OCLN	Reduce viral infectivity	[192]
	miR‐21‐5p	Up	HCV‐3a	Promote HCV life cycle and steatosis	[193]
	miR‐155‐5p	Up	AFP	Potential HCC molecular markers for AFP‐negative HCC patients	[194]
	miR‐199a‐5p	Down	AFP	Potential HCC molecular markers for AFP‐negative HCC patients	[194]
	Let‐7b	Down	NS5B	Suppress HCV replicon activity and down‐regulated HCV accumulation	[195]
	miR‐181c	Down	ATM	Promote apoptosis of HCV‐infected hepatocytes and regress tumor growth in HCC	[196]
	miR‐99a	Down	mTOR/SREBP‐1c	Ameliorate intracellular lipid accumulation and cause inefficient replication and packing of HCV	[197]
	miR16	Up	SMAD7, IRF3	Inhibit IFN production	[198]
	miR‐93‐5p	Up	IFNAR1	Induce inactivation of the IFN signaling pathway	[199]
	miR‐373	Up	IRF5	Promote HCV RNA expression	[200]
	miR‐135a	Up	RIPK2, MTD88, CXCL12	Promote viral genome replication	[201]
	miR‐125a	Up	MAVs, TRAF6	Modulate IFN signaling and promote HCV infection	[202]
	miR‐221	Up	SOCS	Stand as a standalone biomarker for staging various HCV‐associated disorders	[203]
	miR‐542	Up	–	Stand as a standalone biomarker for staging various HCV‐associated disorders	[203]
	Let‐7c	Down	HO‐1	Suppress HCV replication	[204]
	miR‐29c	Down	STAT3	Repress HCV infection via promoting type I IFN response	[205]
	miR‐125b‐5p	Up	HuR	A negative regulator of HCV infection	[206]
	miR‐130a	Down	ATG5	Downregulate HCV replication	[207]
	miR‐185‐5p	Down	GALNT8	Inhibit HCV replication	[208]
	miR‐483	Up	–	Serve as potential noninvasive early diagnostic biomarkers for HCC	[209]
	miR‐335	Up	–	Serve as potential noninvasive early diagnostic biomarkers for HCC	[209]
	miR‐19a	Up	SOCS‐3	Activate fibrosis	[210]
	miR‐192	Up	TGF‐β1	Promote HCV‐mediated hepatic fibrosis	[211]
	miR‐29a	Up	SREBP‐1c, CAV1	A potential biomarker for hepatic disease	[212]
	miR‐10a	Up	RORA, BMAL1	Promote abnormal liver metabolism in cirrhotic liver	[213]
	miR‐150	Down	–	Serve as a predictive marker for detection of cirrhosis progression in HCV infected patients	[214]
	miR‐200a	Up	–	Serve as a promising novel biomarker for liver disease	[215]
	miR‐135a‐5p	Up	PTPRD	Drive malignant progression of HCV‐associated liver disease	[216]
	miR‐138	Down	TERT	Promote HCC cell senescence	[217]
	miR‐30c	Down	PAI‐1	Reduce HCV‐associated CSCs properties in hepatocytes	[218]
	miR‐152	Up	–	A potentially marker of hepatocarcinogenesis in HCV^+^ patients	[219]
	miR‐124	Down	STAT3	Inhibit the differentiation and suppressive functions of MDSCs	[220]
	miR‐148a‐3p	Down	c‐JUN, MAPK	Suppress the proliferation of HCC cells infected with HCV	[221]
LncRNA	GAS5	Up	HCV NS3	Attenuate virus replication	[222]
	HOTAIR	Up	SIRT1	Impair metabolic disorder of liver cell	[223]
	HULC	Up	RXRA	Increase the association of HCV‐core protein with lipid droplets and promote HCV particles release	[224]
	Lnc‐ATV	Up	RIG‐I	Promote viral replication	[225]
	Lnc‐ITPRIP‐1	Up	MDA5	Inhibit HCV replication	[226]
	lnc‐IFI6	Up	JAK–STAT	Promote HCV replication.	[227]
	Lnc‐BISPR	Up	BST2	Promote the antiviral IFN response.	[222]
	Lnc‐ITM2C‐1	Up	ISG	Promote HCV replication	[228]
	aHIF	Down	–	Predict the occurrence of HCC in cirrhotic patients related to chronic viral hepatitis	[229]
	hPVT1	Up	–	Predict the occurrence of HCC in cirrhotic patients related to chronic viral hepatitis	[229]
	ANRIL	Up	–	Predict the occurrence of HCC in cirrhotic patients related to chronic viral hepatitis	[229]
	LINC02499	Down	–	Inhibit proliferation, migration, and invasion abilities of HCC cells in vitro	[230]
	MALAT1	Up	–	Represent a putative noninvasive prognostic biomarker indicating worse liver failure score in HCV‐related HCC patients	[231]
	UCA1	Up	SOCS‐7	Suppress antiviral response	[232]
.	HEIH	Up	–	Serve as a potential biomarker in the HCV‐related HCC	[233]
	CASC2	Up	–	Serve as a biomarker and help in HCC diagnosis induced by HCC	[234]
	TUG1	Down	–	Serve as a biomarker for predicting HCC in HCV patients	[235]
	NEAT1	Down	–	Serve as a biomarker for predicting HCC in HCV patients	[235]
	HOTTIP	Up	–	Play indicative role as noninvasive biomarkers for HCC	[236]
CircRNA	circSERPINA3	Up	E‐cadherin	Serve as sensitive molecular marker for early diagnosis of HCC	[237]
	circPSD3	Up	eIF4A3	Regulate RNA amplification in a proviral manner	[238]
	circSMARCA5	Down	–	Serve as a sensitive predictor of HCC disease	[239]

Abbreviations: AFP, alpha‐fetoprotein; aHIF, hypoxia‐inducible factor‐1α; ANRIL, antisense noncoding RNA in the INK4 locus; ATG5, autophagy related 5; ATM, ataxia telangiectasia mutated protein; BMAL1, brain and muscle arnt‐like protein 1; BST2, bone marrow stromal cell antigen 2; CASC2, cancer susceptibility candidate 2; CAV1, caveolin‐1; circPSD3, circRNA pleckstrin and sect. 7 domain containing 3; CircRNA, circular RNA; circSERPINA3, circular RNA SERPINA3; circSMARCA5, circular RNA SMARCA5; c‐JUN, jun proto‐oncogene, AP‐1 transcription factor subunit; CSC, cancer stem cell; CXCL12, C‐X‐C motif chemokine ligand 12; eIF4A3, eukaryotic translation initiation factor 4A3; GALNT8, polypeptide N‐acetylgalactosaminyltransferase 8; GAS5, growth arrest specific 5; HCC, hepatocellular carcinoma; HCV NS3, hepatitis C virus nonstructural protein 3; HCV, hepatitis C virus; HEIH, hepatocellular carcinoma upregulated EZH2‐associated long noncoding RNA; HO‐1, heme oxygenase‐1; HOTAIR, HOX transcript antisense RNA; HOTTIP, HOXA transcript at the distal tip; hPVT1, human plasmacytoma variant translocation 1; HULC, highly upregulated in liver cancer; HuR, human antigen R; IFN, interferon; IFNAR1, interferon alpha and beta receptor; IRES, internal ribosome entry site; IRF3, interferon regulatory factor 3; ISG, interferon‐stimulated gene; JAK–STAT, Janus kinase‐signal transducer and activator of transcription; LINC02499, long intergenic nonprotein coding RNA 2499; Lnc‐ATV, long noncoding RNA associated with tumor vascularization; Lnc‐BISPR, long noncoding RNA‐BST2 interferon stimulated positive regulator; lnc‐IFI6, long noncoding RNA‐IFI6; Lnc‐ITM2C‐1, long noncoding RNA ITM2C‐1; Lnc‐ITPRIP‐1, long noncoding RNA‐inositol 1,4,5‐trisphosphate receptor interacting protein; LncRNA, long noncoding RNA; MALAT1, metastasis‐associated lung adenocarcinoma transcript 1; MAPK, mitogen‐activated protein kinase; MAVs, mitochondrial antiviral signaling protein; MDA5, melanoma differentiation‐associated gene 5;MiRNA, microRNA; MTD88, myeloid differentiation primary response protein 88; mTOR, mechanistic target of rapamycin; NEAT1, nuclear paraspeckle assembly transcript 1; NS5B, nonstructural 5B; OCLN, occluding; PAI‐1, plasminogen activator inhibitor‐1; PTPRD, protein tyrosine phosphatase receptor type D; RIG‐I, retinoic acid‐inducible gene‐I; RIPK2, receptor interacting serine/threonine kinase 2; RORA, retinoic acid receptor‐related orphan receptor alpha; RXRA, retinoid X receptor alpha; SIRT1, sirtuin 1; SMAD7, SMAD family member 7; SOCS, suppressor of cytokine signaling; SREBP‐1c, sterol regulatory element‐binding protein 1c; STAT3, signal transducer and activator of transcription 3; TERT, telomerase reverse transcriptase; TGF‐β1, transforming growth factor beta‐1; TRAF6, tumor necrosis factor receptor‐associated factor 6; TUG1, taurine upregulated gene 1; UCA1, urothelial cancer associated 1.

##### 4.2.4.1 MicroRNAs

MicroRNAs (miRNAs) are small ncRNA molecules, 19–25 nucleotides in length, that primarily regulate gene expression through translational repression or mRNA degradation [240]. Infections by HBV and HCV significantly alter miRNA expression in host cells, affecting cell proliferation, apoptosis, and the tumor microenvironment, contributing to the malignant transformation of HCC.

HBx promotes tumor formation by regulating miRNA expression in host cells. It downregulates several tumor‐suppressive miRNAs, particularly miR‐15a and miR‐16, through a “sponge” mechanism, where HBx interacts with miRNA binding sites, preventing these miRNAs from performing their gene‐suppressive functions [241]. MiR‐15a and miR‐16 normally inhibit cell proliferation and cancer development, but HBx weakens their suppressive effects, resulting in uncontrolled cell growth and tumor formation [242]. In HBV‐infected liver tissues, in addition to miR‐15a and miR‐16, other key tumor‐suppressive miRNAs such as miR‐145, miR‐199b, let‐7a, and miR‐152 are significantly downregulated [241]. MiR‐145 downregulation is linked to hypermethylation of CDH1, whose reduced expression promotes EMT, increasing tumor cell invasiveness [243]. Additionally, miR‐199b downregulation is closely linked to abnormal Wnt/β‐catenin pathway activation, further driving malignant hepatocyte transformation [244]. Research has shown that miR‐3677‐3p is significantly upregulated in HBV‐related HCC cells, associated with high expression of stem cell markers. It inhibits F‐box protein 31 (FBXO31), reducing ubiquitin‐mediated degradation of forkhead box M1 (FOXM1), ultimately leading to tumor progression and increased sorafenib resistance [245]. Besides downregulating tumor‐suppressive miRNAs, HBx also significantly upregulates oncogenic miRNAs. MiR‐21, a well‐known oncogenic miRNA, is highly expressed in various tumors [246]. In HBV‐associated HCC, HBx induces miR‐21 upregulation by activating the interleukin‐6 (IL‐6)/STAT3 signaling pathway [247]. MiR‐21 inhibits tumor suppressors like programmed cell death 4 (PDCD4), phosphatase and PTEN, suppressing apoptosis, promoting cell proliferation, and driving the malignant transformation of HCC [248].

Unlike HBV, HCV does not integrate its genome into host DNA, but its infection still significantly alters host miRNA expression, particularly in the regulation of tumor‐related genes. MiR‐122, highly expressed in the liver, plays a crucial role in HCV infection, which binds to the 5′‐UTR of HCV RNA, stabilizing the viral RNA and promoting its replication, thereby exacerbating hepatocyte damage and increasing the risk of HCC [249, 250]. Overexpression of miR‐122 also regulates lipid metabolism in the liver, leading to lipid accumulation and cell proliferation, further contributing to liver cancer development [251]. In HCV‐associated HCC, several tumor‐suppressive miRNAs, such as miR‐198 and miR‐145, are downregulated [252, 253]. MiR‐198 downregulation is associated with uncontrolled hepatocyte proliferation, while miR‐145 downregulation disrupts the balance between apoptosis and proliferation, leading to tumor cell malignancy [252, 253]. Furthermore, upregulation of miR‐141 correlates with downregulation of the tumor suppressor gene deleted in liver cancer 1 (DLC‐1), which further promotes HCC development [254]. MiR‐155, a miRNA closely linked to inflammatory responses, plays a role in both liver inflammation and HCV‐related tumorigenesis [255]. Studies have shown that miR‐155 is upregulated in the serum and peripheral mononuclear cells of HCV‐infected patients, potentially promoting tumor progression by inhibiting apoptosis, enhancing cell proliferation, and activating the Wnt signaling pathway [256]. HCV promotes miR‐155 upregulation via the nuclear factor kappa B (NF‐κB) pathway, enhancing tumor cell survival [256]. MiR‐21 is also upregulated in HCV‐associated HCC, targeting and inhibiting SMAD7 in the transforming growth factor beta (TGF‐β) signaling pathway, promoting liver fibrosis and tumorigenesis [257]. MiR‐21 upregulation is not only closely related to fibrosis severity but also serves as a potential biomarker for HCV‐related HCC [258]. In HCV infection, miR‐29, miR‐34, and miR‐223 also exhibit abnormal expression [259, 260]. MiR‐29 inhibits liver fibrosis and tumor metastasis by regulating ECM protein expression, while miR‐34 acts as a tumor suppressor by promoting cell cycle arrest and apoptosis [259]. MiR‐223 is upregulated in late‐stage HCV infection and is associated with the progression of liver fibrosis [260].

In conclusion, both HBV and HCV affect tumor cell proliferation and apoptosis by regulating miRNAs, while also promoting the malignant transformation of HCC through alterations in the tumor microenvironment and immune responses.

##### 4.2.4.2 Long ncRNAs

Long ncRNAs (lncRNAs) are RNA molecules longer than 200 nucleotides that do not encode proteins [261]. Hepatitis viruses contribute to liver cancer development by regulating lncRNAs, affecting gene expression, cell proliferation, apoptosis, and immune responses.

Studies have found that HBV‐encoded proteins, such as HBx and HBsAg, promote hepatocyte malignancy by modulating various lncRNAs. LncRNA opa interacting protein 5‐antisense 1 (OIP5‐AS1) is significantly downregulated in HBV‐positive HCC patients, and its low expression is generally associated with poor prognosis [262]. HBx reduces OIP5‐AS1 levels by inhibiting peroxisome proliferator‐activated receptor α (PPAR‐α) expression [262]. Specifically, OIP5‐AS1 promotes ubiquitination and degradation of the transcription factor sterol regulatory element‐binding protein 1 (SREBP1), inhibiting hexokinase domain containing 1 (HKDC1) transcriptional activity, blocking glycolysis in liver cancer cells, and ultimately restraining tumor progression [262]. LncRNA X‐inactive specific transcript (XIST) is markedly upregulated in HBV‐associated HCC tissues and HepG2.2.15 cells. Research has indicated that XIST promotes HCC development by targeting miR‐192 and regulating TRIM25 expression. Overexpression of XIST inhibits miR‐192, leading to TRIM25 upregulation that enhances HCC cell proliferation and migration [263]. HBsAg plays a crucial role in miRNA regulation of HBV‐related HCC. Research has shown that HBsAg upregulates the oncogenic lncRNA LINC00665 by activating the NF‐κB signaling pathway. LINC00665 is highly upregulated in HBV‐infected liver and HCC tissues, particularly in HBV‐associated HCC, where it promotes tumor cell proliferation, migration, and colony formation, while inhibiting apoptosis [264]. LncRNA high expressed in HCC (HEIH) is also highly expressed in HBV‐related HCC and regulates the expression of cell cycle proteins p15, p16, p21, and p57 through interaction with transcription factor specificity protein 1 (Sp1), promoting cell proliferation and tumor growth. HEIH upregulation is closely associated with HCC progression, particularly in the transition from chronic HBV infection to cirrhosis and HCC. Moreover, HEIH enhances liver cancer cell proliferation by interacting with enhancer of zeste homolog 2 (EZH2) and modulating epigenetic modifications [265]. LncRNA urothelial carcinoma associated 1 (UCA1), upregulated by HBx in HBV‐associated HCC, is closely linked to enhanced cell proliferation and metastasis. UCA1 promotes the G1/S phase transition by recruiting EZH2 to inhibit p27 expression, thereby accelerating HCC development. Additionally, UCA1 functions as a molecular sponge for miR‐216b and miR‐203, enhancing the metastatic potential of tumor cells by regulating these miRNAs [266].

HCV infection is strongly associated with HCC development, with multiple lncRNAs showing abnormal expression during infection, which contributes to liver cancer progression. Research has indicated that lncRNA LINC01189 is upregulated in both HCV infection and HCV‐induced HCC, with high expression correlating with a poor prognosis. LINC01189 promotes HCC cell proliferation and metastasis by sponging miR‐155‐5p [267]. LncRNA highly upregulated in liver cancer (HULC) is significantly upregulated in HCV‐related HCC, promoting liver cancer by regulating autophagy and cell cycle proteins, and inhibiting p21 expression through sponging miR‐675, thereby enhancing HCC cell proliferation. Additionally, HULC facilitates HCV particle release by regulating lipid metabolism, further both advancing viral infection and tumor progression [268]. HCV infection induces the upregulation of lncRNA nuclear‐enriched abundant transcript 1 (NEAT1), which downregulates miR‐9‐5p, leading to overexpression of the oncogene TGF‐β‐induced protein (BGH3) and promoting HCC progression. NEAT1 upregulation is not only linked to tumor development but may also influence antiviral treatment effectiveness, suggesting its multifaceted role in HCV‐related HCC [269]. In conclusion, HBV and HCV play crucial roles in HCC malignancy through the regulation of lncRNAs.

##### 4.2.4.3 Circular RNAs

Circular RNAs (circRNAs) are a type of ncRNA characterized by a stable circular structure in cells, regulating gene expression, cell proliferation, and cancer metastasis through interactions with miRNAs and proteins [270]. Studies have shown that HBV and HCV infections promote HCC development and progression by modulating both host‐derived and viral circRNAs.

Several circRNAs regulate critical molecules involved in HCC development linked to HBV infection. Circ_0000650 regulates TGF‐β2 expression by sponging miR‐6873‐3p, which is closely associated with HBV‐related‐HCC proliferation and metastasis [271]. CircRNAs also influence HBV replication by modulating host‐virus interactions. For instance, circ_0004812 is overexpressed in patients with chronic hepatitis B (CHB) and HBV‐infected liver cancer cells, promoting viral replication by suppressing antiviral immune responses via the circ_0004812/miR‐1287‐5p/follistatin‐like protein 1 (FSTL1) axis [272]. Another critical circRNA, circ_101764, regulates the PI3K/AKT signaling pathway by sponging the miR‐181 family [273]. Research has indicated that HBx upregulates circRNA_101764 expression by activating miR‐181, which enhances liver cell “stemness” and promotes HCC progression [273]. Moreover, circRNA_100338 is closely associated with the metastatic progression of HBV‐related HCC, promoting tumor cell migration and invasion by sponging miR‐141‐3p and regulating metastasis suppressor 1 (MTSS1), whose downregulation is linked to increased metastatic potential in HCC, making the circRNA_100338/miR‐141‐3p/MTSS1 axis essential for HBV‐related HCC progression [274]. Some circRNAs, on the other hand, overlap in their regulatory functions. For instance, circ‐Arf‐like 3 (circ‐ARL3) is highly expressed in HBV‐positive HCC patients and acts as a molecular sponge for miR‐1305, promoting liver cancer by influencing multiple oncogenes [275]. MiR‐1305 inhibits cancer stem cells (CSCs) self‐renewal and tumor proliferation by suppressing oncogenes such as ubiquitin‐conjugating enzyme E2T (UBE2T) and TGF‐β2, while circ‐ARL3 upregulation counteracts miR‐1305 effects, enhancing oncogene expression [275]. Likewise, circ‐ATP synthase 5H (circ‐ATP5H) exhibits significant oncogenic effects in HBV‐related HCC, promoting both HBV DNA replication and tumor cell proliferation by sponging miR‐138‐5p and regulating tumor necrosis factor α (TNF‐α)‐induced protein 3 (TNFAIP3) [276].

Similarly, HCV infection also influences circRNAs involved in HCC development and progression. Dysregulated circRNAs induced by HCV infection are closely linked to liver cancer. Circ‐serpin family A member 3 (circ‐SERPINA3) is significantly upregulated in both plasma of the HCV‐infected patients and HCV‐related HCC patients, promoting cell proliferation and migration by sponging miR‐944 [277]. Circ‐SNF2 related chromatin remodeling ATPase 5 (circ‐SMARCA5) shows abnormal expression in HCV‐related HCC tissues and is negatively correlated with tumor markers like AFP and alkaline phosphatase (ALP), indicating its potential as an early diagnostic marker [239]. Additionally, hsa_circ_0051443 regulates the malignant characteristics of HCC by sponging miR‐331‐3p, further supporting the critical role of circRNAs in HCV‐associated HCC [278].

In summary, chronic HBV and HCV infections contribute to HCC development and progression through the regulation of circRNAs. CircRNAs influence liver cancer development by modulating immune responses, miRNA functions, and key signaling pathways, and may serve as potential biomarkers for early detection and prognosis.

### 4.3 Oxidative Stress

Oxidative stress refers to the excessive accumulation of ROS in cells, overwhelming the cellular antioxidant defense systems [279]. In HCC triggered by viral hepatitis, oxidative stress accelerates malignant transformation by promoting gene mutations, cellular damage, and fibrosis. Prolonged oxidative stress not only causes hepatocyte damage but also triggers inflammatory responses, leading to fibrosis and cirrhosis, key precursors to liver cancer development [280].

In patients with chronic HBV infection, ROS levels in the liver and plasma are significantly elevated, correlating with disease severity and viral replication activity [281]. HBx plays a central role in this process. Studies have shown that the HBx protein interacts with cytochrome *c* oxidase subunit 3 (COXIII) in the mitochondrial respiratory chain, leading to increased mitochondrial ROS (mitoROS) levels [282]. Mitochondrial dysfunction not only enhances ROS production but also activates pyroptosis pathways, upregulating proinflammatory factors such as apoptosis‐associated speck‐like protein containing a CARD (ASC), IL‐1β, IL‐18, and high mobility group B1 (HMGB1) [283]. Additionally, HBV protein accumulation induces ER stress. High levels of viral proteins like HBsAg and HBeAg often misfold during ER processing, triggering the unfolded protein response (UPR). UPR induces intracellular calcium ion release and enhances ROS production, further damaging hepatocytes and promoting fibrosis progression, which creates favorable conditions for liver cancer development [284]. Furthermore, HBV infection suppresses the host antioxidant mechanisms, such as the kelch‐like ECH‐associated protein 1 (KEAP1)/nuclear factor erythroid 2‐related factor 2 (NRF2)/antioxidant response elements (AREs) pathway, reducing levels of antioxidant enzymes like glutathione (GSH) and catalase (CAT). This diminishes cellular antioxidant capacity, exacerbating oxidative stress and fostering a microenvironment conducive to both viral replication and HCC progression [285].

Similarly, HCV infection also significantly elevates oxidative stress in the liver. Several HCV proteins, including the core protein, NS4B, and NS5A, directly or indirectly induce ROS production [286, 287]. The HCV core protein localizes to mitochondria, inhibiting critical components of the electron transport chain, causing mitochondrial dysfunction and increased ROS production [10]. Additionally, the NS5A protein enhances calcium ion uptake by mitochondria and ER, further boosting ROS production [288]. These processes elevate oxidative stress, driving HCC development. In chronic HCV infection, antioxidant enzyme glutathione peroxidase (GSH‐Px), CAT and superoxide dismutase (SOD) levels in the liver are significantly reduced, while ROS levels rise dramatically. Persistent oxidative stress increases free radical levels, leading to DNA adduct formation, protein oxidation, and lipid peroxidation, which accelerates fibrosis progression, exacerbates hepatocyte proliferation, and promotes the accumulation of mutations, ultimately leading to HCC [289].

Oxidative stress promotes HCC malignant transformation through multiple mechanisms. First, ROS directly induce gene mutations by damaging DNA, affecting key tumor suppressor genes or oncogenes, which contribute to tumor initiation and progression [290]. Second, oxidative stress activates multiple proinflammatory pathways, worsening liver inflammation and fibrosis [291]. Additionally, oxidative stress induced by HBV and HCV infections disrupts intracellular signaling through ER stress and mitochondrial dysfunction, which impairs normal mechanisms of cell proliferation and apoptosis while activates oncogenic pathways such as the NLR family, pyrin domain containing 3 (NLRP3) inflammasome and the NF‐κB pathway [292]. Notably, ROS accumulation in cirrhotic patients can reduce intestinal permeability, allowing bacteria and bacterial products to enter the bloodstream, potentially leading to complications like HCC [293]. Studies have shown that 30–40% of cirrhotic patients have bacterial DNA in their blood and ascites, along with significantly elevated plasma levels of lipopolysaccharides (LPS)‐binding protein (LBP) and IL‐6 [294].

In conclusion, hepatitis viruses promote HCC malignant transformation by inducing oxidative stress in host cells. ROS production and accumulation not only damage hepatocytes but also accelerate liver cancer development by promoting inflammation, gene mutations, and fibrosis progression.

### 4.4 Disruption of Gut Microbiota

The liver and gut are interconnected through the “gut–liver axis,” with gut microbiota playing a critical role in the malignant transformation of viral hepatitis into HCC, such as HBV and HCV [295]. Gut microbiota dysbiosis promotes HCC development by affecting liver inflammation, immune regulation, and metabolic function.

Although research on gut microbiota in HBV‐related HCC patients remains limited, existing studies have identified significant alterations in their microbiota. For instance, a study of 90 Asian patients found that the gut microbiota in HBV‐associated HCC (B‐HCC) patients exhibited increased diversity and heterogeneity. Butyrate‐producing bacteria, including *Ruminococcus, Faecalibacterium*, and *Clostridium*, were significantly reduced in B‐HCC patients, which may be closely linked to HCC development [296]. Dysbiosis of *Bifidobacterium* was particularly pronounced in HBV cirrhosis patients, potentially exacerbating liver inflammation and advancing HCC progression [296]. Additionally, *Bacteroidetes* and *Lachnospiraceae incertae sedis* were enriched in HBV–HCC patients with high tumor burden, suggesting their involvement in HCC progression [297]. Microbiota dysbiosis may promote HBV‐driven HCC by increasing short‐chain fatty acid (SCFA) production, weakening the intestinal barrier, and exacerbating chronic liver inflammation [298].

In the same way, HCV infection significantly alters gut microbiota composition and is closely associated with increased gut permeability, bacterial translocation, and liver inflammation. Patients with HCV‐related cirrhosis exhibit reduced gut microbiota diversity, marked by an increase in *Streptococcus* species and a decrease in *Clostridium* [298]. In HCV‐associated HCC patients, the significant reduction in *Bifidobacterium* and *Lactobacillus* levels, along with the marked increase in *Bacteroides* and *Enterobacteriaceae*, further worsen liver inflammation and create a proinflammatory environment that drives HCC development [299]. Early‐stage HCV infection is also associated with transient increases in *Bacteroidaceae* and *Enterobacteriaceae*, potentially laying the groundwork for HCC development [300]. Notably, an increase in *Streptococcus salivarius* is closely linked to tumor occurrence in HCV‐related HCC patients, indicating that the impact of HCV on gut microbiota spans the entire disease process and plays a crucial role in the malignant transformation to HCC [301].

Gut microbiota dysbiosis promotes HCC malignant transformation through several mechanisms. First, increased gut permeability allows bacteria and their metabolites, such as pathogen‐associated molecular patterns (PAMPs) and LPS, to translocate to the liver, where they activate receptors like Toll‐like receptor 4 (TLR4) and TLR9, triggering sustained immune responses and chronic inflammation [302]. Second, the reduction in beneficial SCFA‐producing bacteria, such as *Bifidobacterium* and *Clostridium*, decreases butyrate production, which is essential for maintaining intestinal barrier integrity and immune homeostasis. This metabolic imbalance exacerbates oxidative stress and inflammation in the liver, creating conditions conducive to HCC development [303, 304]. Furthermore, microbial dysbiosis alters the tumor metabolic environment, with certain gut microbes producing metabolites like acetate that fuel tumor cell growth and promote malignant transformation upon entering the liver. The gut microbiota may also influence bile acid metabolism, further modifying the tumor immune microenvironment and facilitating HCC progression [305, 306].

In conclusion, HBV and HCV infections promote the malignant transformation of HCC by altering the composition and function of the gut microbiota, where dysbiosis not only drives chronic liver inflammation and metabolic disturbances but also accelerates HCC development and progression through various mechanisms.

### 4.5 Chronic Inflammation and Immune Evasion

**FIGURE 2:**
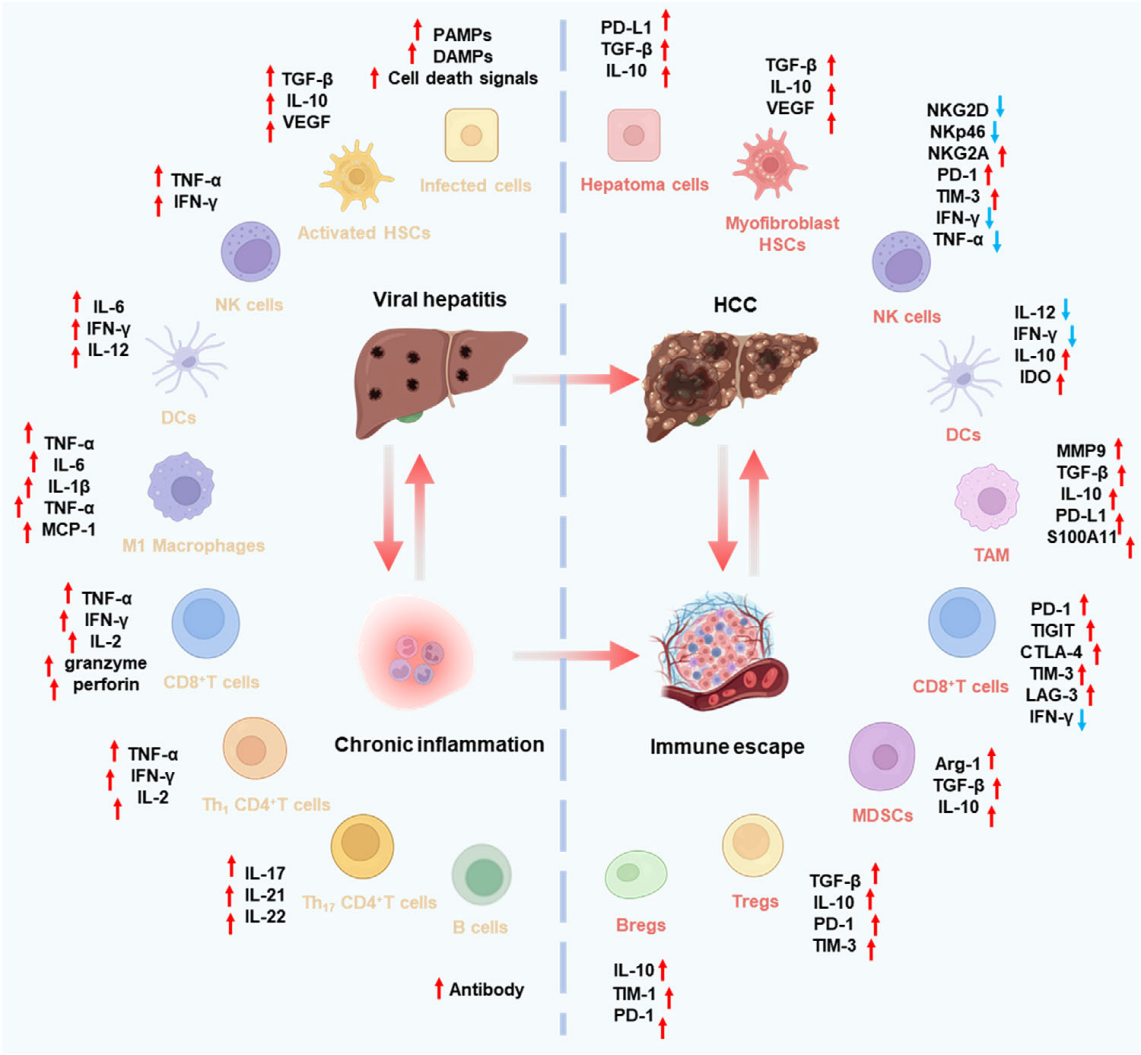
Cell types and characteristics involved in chronic inflammation and immune evasion during the malignant transformation of viral hepatitis to HCC. CTLA‐4, cytotoxic T‐lymphocyte antigen 4; DAMPs, damage‐associated molecular patterns; HCC, hepatocellular carcinoma; IDO, indoleamine 2,3‐dioxygenase; IFN, interferon; IL, interleukin; LAG‐3, lymphocyte activation gene 3; MCP‐1, monocyte chemoattractant protein 1; MMP, matrix metalloproteinases; NKG2A, natural killer group 2 member A; NKG2D, natural killer group 2 member D; NKp46, natural killer p46; PAMPs, pathogen‐associated molecular patterns; PD‐1, programmed death 1; PD‐L1, programmed death ligand 1; S100A11, S100 calcium binding protein A11; TGF, transforming growth factor; TIM, T cell immunoglobulin domain and mucin domain; TNF, tumor necrosis factor; TIGIT, T cell immunoreceptor with Ig and ITIM domains; VEGF, vascular endothelial growth factor.

Chronic inflammation and immune evasion are crucial in the progression from chronic viral hepatitis to HCC. The sustained presence of viruses leads to continuous immune activation and tissue damage, creating a microenvironment enriched with proinflammatory cytokines and ROS. This environment induces persistent liver damage and fibrosis, promotes the accumulation of gene mutations, and increases the likelihood of malignant transformation. Conversely, uncontrollable chronic viral hepatitis induces immune suppression, enabling infected and malignant cells to evade immune surveillance, further accelerating HCC development. Various cells, including hepatocytes, hepatic stellate cells (HSCs), hepatoma cells, and immune cells, play key roles in this process (Figure 2).

#### 4.5.1 Hepatocytes

Following hepatitis virus infection, PAMPs, such as viral nucleic acids and proteins, are detected by pattern recognition receptors (PRRs) on the surface of liver cells, including TLRs and RIG‐I [307]. Activation of receptors such as TLR4, TLR3, and RIG‐I triggers proinflammatory signaling pathways like NF‐κB and STAT3, increasing the secretion of inflammatory cytokines, including IL‐6, TNF‐α, and IFN‐γ, which sustain chronic inflammation by activating immune cells and draw macrophages, Natural killer (NK) cells, and T cells to liver [308, 309]. Persistent inflammation damages hepatocytes and worsens the inflammatory response, thus leading to a “cytokine storm” and continuous liver tissue damage and fibrosis.

Remarkably, in early HBV‐infected hepatocytes, TLR2 expression is significantly downregulated, suppressing inflammatory signaling pathways and reducing cytokine production [310]. Additionally, HBsAg and HBx interfere with the activation of critical proinflammatory transcription factors, including IFN regulatory factor 3 (IRF‐3) and NF‐κB, allowing the virus to evade immune detection [311]. HBV further diminishes antiviral activity by suppressing IFN‐β and IFN‐stimulated genes (ISGs), safeguarding its replication and promoting persistent infection and tumorigenesis [312].

During early HCV infection, activation of TLR3 and RIG‐I induces hepatocytes to secrete large amounts of chemokine C‐X‐C ligand 10 (CXCL10), a process that can occur via nonparenchymal cell (NPC) immune responses independent of type I or type III IFNs, which suggests that HCV regulates hepatocyte–NPC interactions through multiple mechanisms, causing sustained inflammation [313]. Additionally, HCV employs unique mechanisms to evade host immune responses. HCV‐infected hepatocytes modify the expression of immune‐suppressive molecules, including programmed death‐ligand 1 (PD‐L1) and TGF‐β, inhibiting T‐cell activity. While T cells are essential for controlling HCV infection, particularly those of CD4^+^ and CD8^+^ T cells, are significantly impaired in chronic cases, allowing HCV to sustain chronic infection by suppressing T‐cell responses [314]. Moreover, HCV‐infected hepatocytes secrete exosomes and TGF‐β, inducing the expansion of regulatory T cells (Tregs) and further suppressing antiviral responses. Exosomes also carry specific miRNAs that regulate the tumor microenvironment and promote HCC development [315, 316]. In summary, HBV and HCV manipulate hepatocytes through various mechanisms, inducing chronic inflammation and immune evasion, which ultimately promotes HCC development.

#### 4.5.2 Hepatic Stellate Cells

Chronic viral hepatitis induces persistent inflammation and drives HCC progression by activating HSCs, which results in fibrosis. In chronic hepatitis, HSCs transition from a quiescent to an activated state, and produce large amounts of extracellular matrix (ECM) that lead to fibrosis and cirrhosis, creating a microenvironment conducive to HCC development [317].

Activated HSCs are central to fibrosis development. Chronic inflammation from HBV and HCV infections continuously stimulates HSCs, leading to excessive collagen buildup and worsening fibrosis. Studies have shown that activated HSCs secrete cytokines such as TGF‐β through autocrine and paracrine effects, which further drive HSCs activation and fibrosis, creating a vicious cycle [318]. Additionally, HSCs secrete vascular endothelial growth factor (VEGF) and fibroblast growth factor (FGF), promoting tumor angiogenesis and the growth of precancerous and cancerous hepatocytes, thereby creating a tumor‐promoting microenvironment conducive to HCC development [319]. HSCs also play a critical role in immune evasion.

HSCs, by secreting immunosuppressive cytokines like TGF‐β and IL‐10, suppress the activity of immune cells, including NK cells and cytotoxic T lymphocytes (CTLs). In this case, infected hepatocytes can evade immune surveillance and maintain persistent infection [320]. Moreover, research has revealed that HBV and HCV infections upregulate specific genes in HSCs, such as nicotinamide N‐methyltransferase (NNMT) and CD44, which enhance cancer cell migration and immune evasion, accelerating HCC progression [321]. HSC‐secreted TGF‐β induces EMT in cancer cells, enhancing their invasiveness and metastatic potential, thereby driving both tumor progression and fibrosis severity [322]. In summary, HBV and HCV promote chronic inflammation, fibrosis, and HCC progression by activation of HSCs.

#### 4.5.3 Hepatoma Cells

Chronic viral infections induce gradual malignant transformation of hepatocytes, eventually forming tumors. Hepatoma cells, in turn, use various mechanisms to evade immune surveillance, enabling their survival and spread in the immune environment.

Normally, cells present antigens to CTLs via major histocompatibility complex class I (MHC I) molecules, triggering an immune response [323, 324]. However, during HBV and HCV infections, the expression of MHC I in HCC cells is downregulated. Hence, the likelihood of antigen recognition by T cells is reduced, allowing HCC cells to evade CTL‐mediated attacks and promoting their proliferation and spread [325]. Another important immune evasion strategy involves the overexpression of immune checkpoint molecules like PD‐L1 by hepatoma cells, which suppresses T‐cell activity. HBV‐DNA polymerase interacts with poly (ADP‐ribose) polymerase 1 (PARP1), preventing its nuclear translocation and upregulating PD‐L1 expression [326]. PD‐L1 binds to cell death protein 1 (PD‐1) receptors on T cells, inhibiting their function and preventing effective tumor cell attacks. Meanwhile, activation of the PD‐L1/PD‐1 axis reduces T‐cell killing capacity while leading to T‐cell exhaustion, further promoting the survival and spread of tumor [326, 327]. Hepatoma cells also secrete immunosuppressive cytokines such as IL‐10 and TGF‐β, which further dampen the host's antitumor immune response. These cytokines directly suppress effector T cells and promote the activation of Tregs. Increased Treg activity further inhibits CTLs and NK cells, while the accumulation of these immunosuppressive factors in the tumor microenvironment weakens immune defenses, facilitating tumor growth and metastasis [328]. In summary, HBV and HCV can effectively drive immune evasion in liver cancer through multiple pathways, facilitating the malignant transformation of HCC.

#### 4.5.4 CD8^+^ T Cells

CD8^+^ T cells are essential for host antiviral immunity and tumor suppression. They curb viral spread by killing infected hepatocytes directly, secreting proinflammatory cytokines like IFN‐γ, IL‐2, and TNF‐α, and releasing cytotoxic molecules such as granzyme and perforin [329]. Besides, they also contribute to malignant transformation by inducing chronic inflammation in cases of HBV and HCV infections, which promotes aggravation of liver disease. Over time, they gradually become exhausted, hindering the effective clearance of virus‐infected cells, which is closely related to the occurrence of HCC [330].

In chronic HBV infection, CD8^+^ T cells persist in circulation and the liver, yet their function is significantly impaired. Studies have shown that HBV‐specific CD8^+^ T cells express high levels of inhibitory receptors like CTL‐associated antigen 4 (CTLA‐4), PD‐1, T cell immunoreceptor with Ig and ITIM domains (TIGIT), lymphocyte activation gene 3 (LAG‐3) and T‐cell immunoglobulin and mucin domain‐containing protein 3 (TIM‐3) [331]. The continued expression of these inhibitory receptors suppresses CD8^+^ T cell functions, limiting their production of antiviral cytokines and ability to eliminate virus‐infected hepatocytes, which hinders viral clearance and perpetuates chronic inflammation, raising the risk of HCC [330]. Furthermore, although HBV‐specific CD8^+^ T cells exhibit functional exhaustion, they still retain some inflammatory activity. They secrete low levels of IFN‐γ and recruit other immune cells, such as macrophages, into the liver, exacerbating chronic inflammation [332]. Macrophages, in turn, secrete proinflammatory cytokines like TNF‐α, IL‐6, and monocyte chemoattractant protein‐1 (MCP‐1), which accelerate liver damage and fibrosis, ultimately contributing to HCC progression [333].

In chronic HCV infection, CD8^+^ T cell exhaustion follows a similar mechanism. Despite sufficient numbers of HCV‐specific CD8^+^ T cells, their function is severely impaired, limiting effective viral clearance [334]. As in HBV infection, chronic inflammation from HCV infection related to CD8^+^ T cells promotes tumorigenesis and progression via enhanced oxidative stress and proinflammatory pathways [335].

In addition to virus‐specific CD8^+^ T cells, nonspecific CD8^+^ T cells also play a crucial role in chronic hepatitis and HCC progression. Activated during chronic inflammation, these cells secrete proinflammatory cytokines such as IFN‐γ, driving the inflammatory response, though they cannot directly eliminate viruses [336]. In summary, despite functional suppression from high inhibitory receptor expression, CD8^+^ T cells still recruit inflammatory cells and secrete proinflammatory factors, causing repeated cycles of hepatocyte injury, regeneration, and mutation accumulation. The long‐term cycle of inflammation and regeneration fosters liver cancer development.

#### 4.5.5 CD4^+^ T Cells

CD4^+^ T cells hold much significance in the immune system, particularly in antiviral and antitumor responses. In chronic HBV and HCV infections, CD4^+^ T cells produce cytokines that regulate immune responses by differentiating into various effector subgroups, which promote HCC development through chronic inflammation [337].

Th1 cells, a subset of CD4^+^ T cells, mediate immune responses by secreting proinflammatory cytokines like IFN‐γ, TNF‐α, and IL‐2, activating CD8^+^ T cells [338]. In HBV‐related HCC, Th1 cell frequency is significantly reduced, especially in tumor tissues, which weakens antiviral and antitumor immunity, facilitating HCC progression [339]. In contrast, Th17 cells secrete cytokines such as IL‐17, IL‐21, and IL‐22, and sustain inflammation. In chronic HBV infection, an imbalance in the Th1/Th17 ratio, particularly an increase in Th17 cells, correlates with liver damage, fibrosis, and late‐stage liver disease [340]. Increased tumor‐infiltrating Th17 cells are poor prognostic indicators in HBV‐related HCC, where a high Th17/Th1 ratio predicts poor survival, especially in patients undergoing liver resection [341, 342].

T follicular helper cells (Tfh cells), another CD4^+^ T cell subset, support B cell differentiation and antibody production, playing a key role in antiviral immunity [343]. In HBV‐related HCC, circulating Tfh cell frequency is significantly reduced, and their function deteriorates as the disease progresses. Tumor tissue infiltration of Tfh cells is also markedly decreased, which weakens B cell‐mediated adaptive immunity and promotes chronic liver inflammation and HCC development [344].

CD4^+^ CTLs, which can directly kill virus‐infected cells by secreting granzyme and perforin, are also impaired in HBV‐related HCC. They gradually disappear or lose function as HBV‐related HCC develops, correlating with poor outcomes [345]. CD4^+^ CTL dysfunction is linked to increased Tregs, further weakening tumor immune surveillance [346]. In HCV infection, CD4^+^ T cells are similarly impaired. Chronic HCV patients exhibit reduced CD4^+^ T cell numbers and diminished function, with lower levels of IL‐2 and IFN‐γ secretion, indicating a decline in activation and antiviral efficacy [347].

Tregs maintain immune tolerance by suppressing effector T cell functions, but in chronic HBV and HCV infections, Treg frequency is significantly increased, leading to immune suppression and persistent viral infection [348]. Tregs inhibit CD8^+^ T cell proliferation and cytotoxicity by secreting immunosuppressive cytokines like IL‐10 and TGF‐β, promoting immune evasion in HCC [348]. In the HCC tumor microenvironment, Tregs suppress both antiviral and antitumor immunity by exhibiting higher levels of PD‐1 expression, enhancing their suppressive effects and allowing HCC to progress under immune surveillance, closely associated with poor prognosis and increased tumor aggressiveness [349].

In conclusion, hepatitis viruses promote chronic inflammation and HCC development by impairing CD4^+^ T cell function. In HBV and HCV infections, CD4^+^ T cell function becomes exhausted, with reduced Th1 cells, elevated Th17 and Treg cells, and a cycle of chronic inflammation and immune suppression, which hinders viral clearance and creates an environment conducive to HCC progression.

#### 4.5.6 B Cells

B cells, a key component of adaptive immunity, produce antibodies and regulate immune responses to help eliminate viral infections [350]. However, during chronic HBV and HCV infections, B cell dysfunction contributes to viral persistence and promotes HCC development by fostering a chronic inflammatory environment [351].

In the early stages of HBV infection, B cells exert antiviral effects by producing anti‐HBs and anti‐HBc antibodies that neutralize the virus and prevent its spread [352]. However, in chronic HBV infection, B cells become functionally impaired and fail to produce sufficient antibodies, particularly HBsAg‐specific antibodies. B cells often exhibit an atypical memory phenotype (CD21^−^CD27^−^) and increased PD‐1 expression in patients suffering from chronic HBV, leading to reduced functionality and antibody production [352]. The B cell dysfunction enables HBV persistence, activates the host immune system, and triggers chronic inflammation. Furthermore, HCV impairs B cell‐mediated humoral responses, limiting the production of neutralizing antibodies against the virus, which creates a chronic inflammatory environment [353].

The inhibition of B cell function not only hampers HBV and HCV clearance but also promotes HCC development through persistent chronic inflammation. One mechanism by which hepatitis viruses induce B cell dysfunction is through increased regulatory B (Breg) cells, which secrete IL‐10 and TGF‐β, suppressing effector T cells and other immune responses [354, 355]. Breg cells also inhibit CD4^+^ T cell production of granzyme and perforin through TIM‐1 expression, weakening T cell cytotoxicity, which promotes immune evasion and contributes to HCC progression [356]. Furthermore, in postoperative HCC patients, Breg cell frequency is significantly increased and correlates with higher HBeAg levels and HBV‐DNA copies. It suggests that Breg cells are crucial for tumor progression, as they facilitate the evasion of immune attacks by tumor cells and enhance HCC expansion [352].

Briefly, while B cells are essential for antiviral immunity, their dysfunction in chronic HBV and HCV infections hinders viral clearance and leads to chronic inflammation. The increased presence of Breg cells further intensifies an immunosuppressive environment that facilitates HCC progression through mechanisms of immune evasion.

#### 4.5.7 NK Cells

NK cells are essential components of the innate immune system, capable of directly killing virus‐infected cells and playing a key role in early infection control [357]. However, during chronic HBV and HCV infections, NK cell function is modulated by the virus, leading to immune dysfunction that promotes chronic inflammation and the progression to HCC.

In the early stages of HBV infection, NK cells inhibit viral replication and spread by secreting IFN‐γ and TNF‐α, as well as through cytotoxic activity [358]. However, prolonged NK cell activation exacerbates hepatocyte damage and contributes to chronic liver inflammation [359]. Studies regarding HBV transgenic mouse models have shown that NK cells promote EMT pathways through IFN‐γ secretion, leading to hepatocyte injury and facilitating HCC development [360]. As HBV infection persists, the virus regulates NK cell function through various mechanisms, suppressing their antiviral capabilities. For instance, HBsAg inhibits JAK–STAT3 signaling pathway in NK cells, impeding viral clearance and promoting the progression from chronic hepatitis to HCC [361]. Additionally, chronic HBV infection upregulates inhibitory receptors on NK cells, such as PD‐1, TIM‐3, and NK group 2 member A (NKG2A), reducing their ability to secrete IFN‐γ and TNF‐α, which not only hinders the clearance of infected hepatocytes but also perpetuates chronic inflammation [362, 363]. NK cells also contribute to fibrosis by secreting proinflammatory cytokines, such as IL‐4 and IL‐13, which activate HSCs [364].

During HCV infection, NK cells play antiviral and immune regulatory roles in the early stages. In acute HCV infection, NK cells exhibit high cytotoxicity and IFN‐γ secretion, effectively suppressing viral replication [365]. However, as HCV infection becomes chronic, the virus diminishes NK cell antiviral activity by downregulating activating receptors, such as NKp46 and NKp30 [366]. Similar to HBV infection, HCV upregulates inhibitory receptors, such as NKG2A, reducing NK cell cytolytic capacity and IFN‐γ secretion, facilitating viral persistence and chronic liver inflammation [367, 368]. Additionally, the HCV E2 protein binds to CD81 on NK cells, further suppressing their function, which prevents viral clearance and creates conditions that support HCC development [369, 370].

Notably, miR‐146a expression is significantly elevated in NK cells from patients with HBV‐ and HCV‐related HCC, downregulating NK cell activity and reducing IFN‐γ and TNF‐α production, which renders them unable to effectively eliminate cancerous hepatocytes and promotes tumor growth and metastasis [371]. Furthermore, HBV and HCV secrete exosomes that directly affect NK cell function and survival, contributing to viral transmission and the creation of the tumor microenvironment, thereby driving HCC progression [372, 373].

In summary, while NK cells are crucial for antiviral immunity, their function is suppressed by viral mechanisms in chronic HBV and HCV infections. Downregulation of activated receptors and upregulation of inhibitory receptors on NK cells not only hampers viral clearance but also promotes HCC development by inducing chronic inflammation and creating an immunosuppressive environment.

#### 4.5.8 Macrophages

Macrophages, particularly Kupffer cells, the liver‐resident macrophages, play a critical role in hepatitis virus infections and in chronic inflammation‐induced malignant transformation, leading to HCC [374]. As primary liver immune cells, Kupffer cells are persistently activated by viral infections, releasing proinflammatory cytokines and contributing to both chronic hepatitis and fibrosis [375].

In chronic HBV and HCV infections, liver macrophages present two primary functional states: proinflammatory M1 and immunosuppressive M2 types. M1 macrophages are activated during the acute infection phase, releasing cytokines like TNF‐α, IL‐6, and IL‐1β to control infection [334]. In chronic infection, macrophages gradually shift toward the immunosuppressive M2 phenotype, with M2 macrophages, especially tumor‐associated macrophages (TAMs), promoting immune evasion in the tumor microenvironment by secreting inhibitory cytokines such as IL‐10 and TGF‐β, thereby fostering immune escape and tumor growth [376]. In HBV and HCV infections, persistent viral stimulation induces M2 macrophage polarization, suppressing T cell function to support tumor growth, and releasing ECM remodeling factors, such as MMP‐9, thereby enhancing cancer cell invasion and metastasis [377].

In HBV infection, viral factors such as HBx protein and HBV DNA polymerase regulate macrophage activity through multiple mechanisms. HBx protein induces high IL‐8 levels in macrophages via the mitogen‐activated protein kinase/extracellular signal‐regulated kinase (MEK/ERK) pathway, activating TGF‐β in liver sinusoidal endothelial cells and promoting immunosuppressive Treg cell accumulation via the IL‐8/C‐X‐C motif chemokine receptor 1 (CXCR1) axis [378]. The process strengthens immunosuppressive effects in the tumor microenvironment, enhancing tumor angiogenesis and invasiveness [379]. Additionally, HBV DNA polymerase interacts with PARP1 in macrophages, blocking its nuclear translocation and increasing PD‐L1 expression. Elevated PD‐L1 weakens T cell‐mediated tumor cell killing in macrophages, enabling tumor cells to evade immune surveillance through checkpoint pathways [380]. Furthermore, Kupffer cells engage with HBcAg via TLR2, leading to CD8^+^ T cell exhaustion and reduced antiviral immunity. This interaction creates an environment that not only fails to eliminate the virus but also encourages chronic inflammation and cancer development [381].

In HCV infection, chronic inflammation is primarily driven by NF‐κB and STAT3 pathway activation in macrophages [382]. HCV nonstructural protein NS5A activates the NF‐κB pathway via TNF receptor (TNFR)‐associated factor 2 (TRAF2), inducing the release of IL‐6 and TNF‐α, which damages hepatocytes and promotes uncontrollable chronic inflammation [383]. Additionally, macrophages detect viral RNA via TLR2, triggering the NLRP3 inflammasome and inducing IL‐1β and IL‐18 release, which amplify local inflammation and activate HSCs, thereby promoting fibrosis [384, 385]. Furthermore, HCV modulates host miRNA expression to enhance immunosuppressive function of macrophages. HCV infection upregulates miR‐155 expression, promoting M2 macrophage polarization and accelerating tumor cell proliferation and migration by inhibiting src homology 2‐containing inositol‐5′‐phosphatase 1 (SHIP1) expression. The miR‐155/SHIP1 axis establishes a potent protumor pathway in HCV‐infected livers, maintaining immunosuppressive conditions in the tumor microenvironment [386].

Significantly, within the tumor microenvironment of virus‐associated HCC, interactions between macrophages and CSCs further drive tumor malignancy. Single‐cell sequencing analyses reveal that stem‐associated HCC cell subclones such as CD24^+^, CD47^+^, and intercellular adhesion molecule 1 (ICAM1)^+^ subsets, are more likely to establish ligand–receptor‐based communication with macrophages [387]. The interaction drives further M2 TAM polarization and release of tumor‐promoting factors like S100 calcium‐binding protein A11 (S100A11), enhancing the stemness and invasiveness of CSCs, which promotes tumor stemness and drug resistance [387].

In conclusion, HBV and HCV modulate macrophages to achieve immune evasion, induce sustained chronic inflammation, and create an immunosuppressive tumor microenvironment, ultimately promoting the malignant transformation of HCC. Through various molecular pathways, such as MEK–ERK, NF‐κB, and STAT3, macrophages maintain inflammatory responses and immune evasion effects, accelerating HCC progression through interactions with tumor cells.

#### 4.5.9 Dendritic Cells

Dendritic cells (DCs) are crucial antigen‐presenting cells that initiate host immune responses [388]. However, HBV and HCV modulate DC function to induce chronic inflammation and hamper immune system's ability to clear infections, promoting the malignant transformation into HCC.

In chronic HBV infection, DC function is significantly impaired. Studies have indicated that plasmacytoid DCs in CHB patients, affected by IL‐6 receptor signaling blockage, exhibit marked functional decline, further leading to DC dysfunction [389]. HBV also disrupts interaction between NK cells and DCs, weakening the overall antiviral response [390]. HBV impairs DC maturation without affecting their expression of human leukocyte antigen class I (HLA‐I), enabling DCs to evade NK cell lysis and remain immature, while the increased expression of IL‐10 and the immunosuppressive enzyme indoleamine 2,3‐dioxygenase (IDO) in these immature DCs reduces NK cell proliferation [390]. Normally, mature DCs secrete IL‐12, a key cytokine for Th1 polarization, which activates CD8^+^ T cells and strengthens antiviral and antitumor immunity. However, this function is severely suppressed in HBV infection, leading to failure in antiviral responses [391].

Similarly, HCV infection significantly affects DC function. Research has found that monocyte‐derived DCs in chronic HCV patients fail to mature properly, suppressing the ability to stimulate allogeneic T cells and diminishing IFN‐γ production [392]. Mechanistically, the HCV core protein interacts with the globular C1q (gC1q) receptor on DCs, inhibiting IL‐12 production and suppressing DC maturation, which skews CD4^+^ T cell differentiation toward a Th2 phenotype, weakening Th1‐mediated antiviral responses. This Th2‐biased response not only reduces antiviral efficacy but also promotes chronic inflammation, driving HCC development [393]. Additionally, HCV binds to CD81 receptor on DCs, further suppressing cytokine production, particularly IFN‐γ, impairing NK cell and CD8^+^ T cell function. This facilitates viral persistence and exacerbates chronic liver inflammation, contributing to fibrosis and HCC progression [394, 395].

In brief, hepatitis viruses manipulate DC function to maintain chronic inflammation, accelerating malignant transformation into HCC. Impaired DC function leads to weakened antiviral immunity, viral persistence, and chronic inflammation, being important drivers of liver cancer development.

#### 4.5.10 Myeloid‐Derived Suppressor Cells

Myeloid‐derived suppressor cells (MDSCs) play a significant immunoregulatory role in chronic hepatitis virus infections, particularly in HBV infection [396]. They are immunosuppressive cells accumulated in tissues such as liver, where they suppress the function of effector T cells, especially the antiviral activity of CD8^+^ T cells, which contribute to maintenance of chronic inflammation and promotes tumor progression [397]. During chronic HBV infection, MDSCs induce immune tolerance and facilitate immune evasion through various mechanisms. Studies have revealed that MDSCs accumulating in the liver during HBV infection inhibit the proliferation of HBsAg‐specific T cells, weakening the host antiviral immune response [398]. Additionally, MDSCs secrete high levels of immunosuppressive cytokines, such as TGF‐β and IL‐10, which further impair CD8^+^ T cell function, leading to their gradual exhaustion and reduced ability to effectively clear the virus [399]. In summary, MDSCs modulate the immune microenvironment by suppressing antiviral immunity and sustaining chronic inflammation, thereby acting as key drivers of hepatitis virus‐induced malignant transformation into HCC.

### 4.6 Activation of Oncogenic Pathways

**FIGURE 3:**
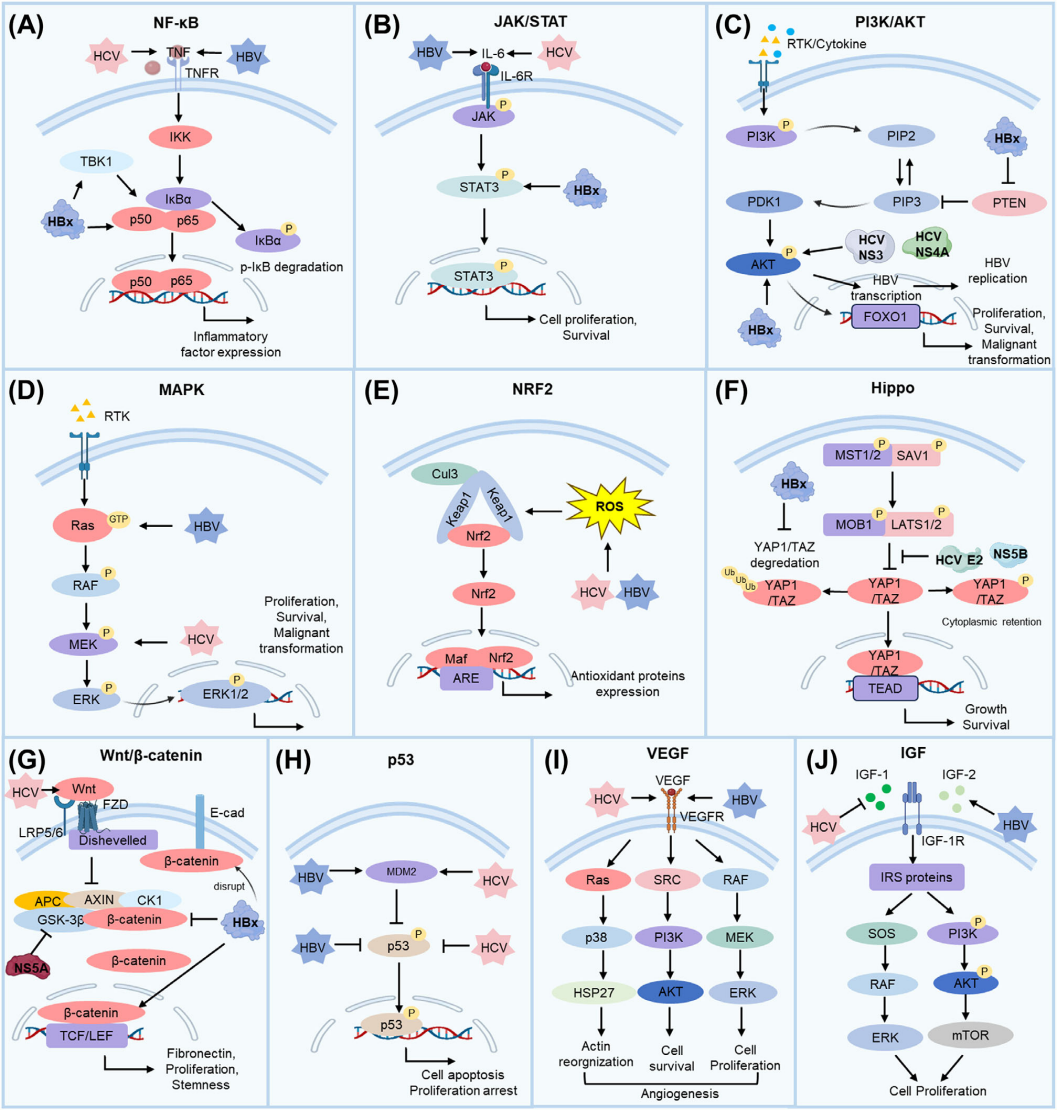
Signaling pathways involved in the malignant transformation of viral hepatitis to HCC. HBV and HCV impact signaling pathways including NF‐κB (A), JAK/STAT (B), PI3K/AKT (C), MAPK (D), NRF2 (E), Hippo (F), Wnt/β‐catenin (G), p53 (H), VEGF (I), and IGF (J), promoting malignant transformation. ARE, antioxidant response element; APC, antigen‐presenting cell; AXIN, axis inhibition protein; CK1, casein kinase 1; Cul3, cullin 3; ERK, extracellular regulated protein kinases; FZD, frizzled receptor; FOXO1, forkhead box O1; GSK‐3β, glycogen synthase kinase 3β; GTP, guanosine triphosphate; HBV, hepatitis B virus; HCC, hepatocellular carcinoma; HCV, hepatitis C virus; HSP27, heat shock protein 27; IGF, insulin like growth factor; IKK, inhibitor of kappa B kinase; IκBα, inhibitor kappa B alpha; JAK/STAT, Janus tyrosine kinase/signal transducers and activators of transcription; Keap1, kelch‐like ECH‐associated protein 1; LRP5/6, low‐density lipoprotein‐related receptors 5/6; LATS, large tumor suppressor kinase 1; MAPK, mitogen‐activated protein kinase; MDM2, murine double minute 2; MEK, mitogen‐activated extracellular signal‐regulated kinase; MST1/2, mammalian Ste20‐like kinases 1/2; MOB1, Mps One binder 1; mTOR, mammalian target of rapamycin; NF‐κB, nuclear factor kappa B; NRF2, nuclear factor erythroid 2‐related factor 2; PIP2, phosphatidylinositol‐4,5‐bisphosphate; PIP3, phosphatidylinositol‐3,4,5‐trisphosphate; PI3K/AKT, phosphoinositide 3‐kinase/protein kinase B; PDK1, pyruvate dehydrogenase kinase 1; PTEN, phosphatase and tensin homolog; RAF, rapidly accelerated fibrosarcoma; ROS, reactive oxygen species; RTK, receptor tyrosine kinase; SOS, son of sevenless; SRC, sarcoma oncogene; TAZ, transcriptional coactivator with PDZ‐binding motif; TEAD, transcriptional enhanced associate domain; TCF/LEF, T cell factor/lymphoid enhancer factor; VEGF, vascular endothelial growth factor; YAP1, yes‐associated protein 1; TBK1, TANK‐binding kinase 1.

In chronic viral hepatitis, oncogenic pathways activated by viral proteins offer critical insights into HCC development mechanisms. Deeper insights into pathways related to malignant transformation may help identify potential therapeutic targets. Key signaling pathways in the malignant transformation from viral hepatitis to HCC include NF‐κB, JAK/STAT, PI3K/AKT, MAPK, NRF2, Hippo, Wnt/β‐catenin, p53, VEGF, and IGF (Figure 3).

#### 4.6.1 Nuclear Factor‐κB

NF‐κB is a transcription factor complex composed of multiple subunits, including p105/p50, p100/p52, RelA (p65), cRel, and RelB, which plays a critical role in cellular processes such as growth, differentiation, proliferation, apoptosis, angiogenesis, and immune responses [400]. In hepatitis virus infections, aberrant activation of the NF‐κB pathway is closely linked to inflammation, fibrosis, and tumor progression.

HBV activates the NF‐κB pathway through various mechanisms, with its encoded protein HBx playing a central role. HBx induces oxidative stress and promotes proinflammatory cytokine expression, thereby activating NF‐κB pathway and driving the malignant transformation of hepatocytes [401, 402]. Studies have shown that HBx decreases cytoplasmic levels of p105 and p50 while promoting the phosphorylation and degradation of NF‐κB inhibitor alpha (IKBα), which releases RelA and prolongs NF‐κB activation [403]. Additionally, HBx interacts with TANK‐binding kinase 1 (TBK1), further enhancing NF‐κB activity, which is strongly associated with HCC invasiveness and metastasis [404]. HBx also indirectly activates NF‐κB by modulating multiple signaling pathways, including PIK3C, Ras/rapidly accelerated fibrosarcoma (Raf) /MAPK, proto‐oncogene tyrosine‐protein kinase Src (Src), and JAK–STAT, all of which contribute to IKBα degradation and persistent NF‐κB activation, triggering procarcinogenic signals [405]. NF‐κB activation driven by HBx promotes hepatocyte proliferation, dysregulated apoptosis, and accelerates HCC progression by increasing the expression of proinflammatory cytokines such as IL‐6, IL‐8, and CXCL2, which are crucial in the inflammatory response, and fostering liver fibrosis, thereby creating a microenvironment conducive to HCC transformation [406, 407].

HCV also promotes HCC development by activating the NF‐κB pathway, both directly and indirectly, enhancing inflammation and tumor progression. The HCV core protein induces proinflammatory cytokines such as TNF‐α, thereby activating the NF‐κB pathway through TNFR activation. On the other hand, the core protein also binds to the cytoplasmic domain of TNFR1, mimicking TNF‐α signaling and amplifying NF‐κB activation [408]. Additionally, HCV promotes Th17 cell activation, leading to secretion of IL‐17 and IL‐22, which further stimulate NF‐κB activity [409]. Beyond the core protein, other HCV proteins, including NS4A, and NS5A, activate NF‐κB pathway by inducing oxidative stress. Once activated, NF‐κB translocates to the nucleus and initiates the expression of genes like cyclooxygenase‐2 (COX‐2) and IL‐8, which are crucial for inflammation and tumor microenvironment formation [410]. While HCV activates NF‐κB through multiple mechanisms, some studies identify that certain HCV proteins (such as NS3 and NS5B) can inhibit TNF‐α‐induced NF‐κB activation, potentially allowing HCV to modulate immune responses and evade immune surveillance, thus contributing to viral persistence [411]. Overall, hepatitis viruses promote a tumorigenic microenvironment and drive HCC development by modulating NF‐κB pathway through oxidative stress and proinflammatory signaling.

#### 4.6.2 Janus Kinase/Signal Transducer and Activator of Transcription

The JAK–STAT signaling pathway, mediated by cytokines such as interleukins and IFNs, plays a critical role in controlling gene expression involved in cell proliferation, survival, and immune regulation [412]. In viral hepatitis, abnormal activation of the JAK–STAT pathway is a significant mechanism contributing to hepatocyte malignant transformation and HCC development.

In HBV infection, the viral protein HBx enhances JAK–STAT activity by interacting with JAK1, increasing its kinase activity, and upregulating STAT3 phosphorylation, leading to sustained activation. This, in turn, activates genes related to cell proliferation and survival, such as cyclin D1 (CCND1) and myeloid cell leukemia 1 (MCL1), promoting uncontrolled hepatocyte proliferation [413]. Additionally, HBx activates STAT3 to induce Twist, a gene promoting EMT, increasing tumor invasiveness and metastatic potential [414]. In HBV‐related HCC, IL‐6 is a key activator of the JAK–STAT pathway. It binds to its receptor, glycoprotein 130 (GP130), initiating JAK1 phosphorylation and STAT3 signaling, which further drive hepatocyte malignant transformation [415]. Elevated IL‐6 levels are strongly linked to HCC development, particularly in men. Mechanistically, estrogen can inhibit IL‐6 secretion from macrophages, reducing HCC risk in women [416].

HCV also accelerates liver cancer progression by modulating the JAK–STAT pathway. HCV infection increases IL‐6 and other proinflammatory cytokines production, leading to STAT3 activation, which drives uncontrolled hepatocyte proliferation and upregulation of antiapoptotic genes such as B‐cell lymphoma‐extra‐large (Bcl‐xL) and CCND1, promoting HCC development [417]. In HCV‐related HCC patients, overactivation of STAT3 is closely linked to tumor progression and poor prognosis, underscoring the pivotal role of JAK–STAT pathway in HCV‐induced HCC [415]. Notably, as viral load increases in the later stages of infection, HCV upregulates SOCS‐1, a negative regulator of the JAK–STAT pathway. HCV infection increases SOCS protein expression through various mechanisms specific to different HCV proteins. Specifically, the HCV protein p7 induces SOCS‐3 via STAT3‐ and ERK‐mediated pathways. Conversely, SOCS‐7 expression induced by HCV core protein genotype 3a operates independently of STAT3 and may be regulated by PPAR‐γ activity. Elevated SOCS‐1 levels inhibit the antiviral effects of type I and type III IFN signaling, creating conditions favorable for viral replication and tumorigenesis [418].

Both HBV and HCV promote HCC by modulating the JAK–STAT pathway, enhancing cell proliferation, EMT, and accelerating tumor growth and metastasis. Dysregulation of the pathway is critical for HCC development and progression in chronic viral hepatitis.

#### 4.6.3 Phosphoinositide 3‐Kinase/Protein Kinase B

The PI3K/AKT signaling pathway plays a crucial role in regulating cellular processes such as proliferation, survival, metabolism, and migration, and its dysregulation is closely linked to HCC development [419]. Hepatitis viruses, particularly HBV and HCV, activate the pathway through multiple mechanisms, promoting HCC onset and progression.

HBV interacts with the PI3K/AKT pathway primarily through HBx, driving HCC malignancy. HBx regulates the PI3K/AKT pathway by activating AKT and inhibiting GSK‐3β, stabilizing β‐catenin and upregulating CCND1 expression, which leads to uncontrolled cell proliferation and carcinogenesis [123, 420]. Different HBx isoforms exhibit distinct roles in cell survival. The HBx–S31 isoform, phosphorylated at Ser31, activates AKT, promoting antiapoptotic effects and tumor growth, while the HBx–L31 isoform, lacking the phosphorylation site, induces apoptosis and inhibits tumor formation [421]. Persistent AKT activation supports HCC progression by inhibiting apoptosis, enhancing cell survival through phosphorylation of transcription factors such as c‐Myc and NF‐κB, which accelerates cell cycle and tumor growth [422, 423].

In a similar manner, HCV promotes HCC development by activating the PI3K/AKT pathway. HCV NS3 protein interacts with key regulators such as p21 and p53, disrupting cell cycle control and activating epidermal growth factor receptor (EGFR) signaling, which further enhances PI3K/AKT activation. This signaling promotes downstream proliferation and survival pathways, driving malignant transformation [424]. Additionally, the HCV NS4A protease activates the PI3K/AKT signaling pathway, promoting infected cell growth and contributing to HCC progression [425]. The PI3K/AKT pathway also interacts with other oncogenic pathways, such as the Ras/ERK signaling pathway, forming a complex network that coregulates HCC development in hepatitis virus infection [426].

In summary, hepatitis virus promotes HCC by activating the PI3K/AKT pathway, which enhances cell proliferation and survival, driving further cancer progression.

#### 4.6.4 Mitogen‐Activated Protein Kinase

The MAPK pathway, comprising Ras, Raf, MEK, and ERK, is a notable signaling cascade activated by external growth factors or cytokines. Ras, a critical protein in the pathway, binds to guanosine triphosphate (GTP), activating Raf, which in turn activates MEK and ERK [427]. Hepatitis viruses, including HBV and HCV, hijack the pathway, activating the Ras–ERK signaling cascade to upregulate cell cycle proteins, as well as oncogenes, ultimately driving abnormal cell proliferation and malignant transformation.

In HBV infection, the viral protein HBx directly impacts MAPK pathway activation. HBx interferes with Ras–GTP turnover, sustaining Ras and MAPK pathway activation, which also enhances EGFR expression, further promoting the Ras/MEK/ERK cascade [428]. The amplified signaling drives abnormal hepatocyte proliferation and inhibits apoptosis, allowing the virus to persist and replicate [429]. Moreover, HBx activates the ERK pathway, upregulating the cell cycle inhibitor cyclin‐dependent kinase inhibitor 1 (p21Cip1) and inducing G2‐phase cell cycle arrest, which benefits HBV replication as the virus thrives during the G1 and G2 phases [430]. Additionally, HBV middle surface antigen activates the Ras/MEK/ERK pathway through a protein kinase C (PKC)‐dependent mechanism, further enhancing the survival of HBV‐infected cells and contributing to tumor formation [431].

Persistent EGFR activation by HCV triggers the MAPK/ERK signaling pathway, enhancing multiple oncogene expression. Microarray analysis has shown that HCV infection significantly upregulates genes related to inflammation and angiogenesis, including amphiregulin (AREG), IL‐8, and C‐C motif chemokine ligand 20 (CCL20), which accelerate HCC progression by promoting inflammation and angiogenesis in the tumor microenvironment [432]. The HCV core protein, key factor in HCC development, affects the expression of transcription factors, such as polyoma enhancer‐activator 3 (PEA3), serum response factor (SRF), and c‐Fos by activating the MAPK/ERK pathway [433]. Specifically, the core protein activates the Raf/MEK/ERK cascade, driving abnormal proliferation and antiapoptotic responses in liver cells. Further research has shown that core protein acts directly upstream of MEK or promote oncogenic gene expression by enhancing downstream transcription factor ETS‐like transcription factor (ELK1) activity [434]. Activation of these pathways increases liver cell proliferation and enhances migration and invasion by upregulating critical molecules like matrix metalloproteinases MMP‐2 and MMP‐9 [3].

In conclusion, aberrant activation of the MAPK pathway plays a pivotal role in HCC transformation from viral hepatitis infection. MAPK activation disrupts cell cycle regulators, enabling viral replication while preventing apoptosis. Sustained MAPK activation also triggers transcription factors like STAT3, enhancing tumor cell survival, invasiveness, and metastatic potential, exacerbating HCC malignancy and worsening patient prognosis.

#### 4.6.5 Nuclear Factor Erythroid 2‐Related Factor 2

NRF2 is a notable transcription factor involved in the cellular response to oxidative stress, regulating antioxidant defense mechanisms by controlling AREs [435]. Under normal conditions, NRF2 protects cells from ROS damage. However, during viral infections, viruses such as HBV and HCV hijack NRF2 activation to evade the host immune system, promoting tumorigenesis and disease progression [436].

In HBV infection, viral proteins like HBx and the large surface proteins (LHBs) activate the NRF2 pathway. HBx, in particular, activates NRF2 through c‐Raf and MEK pathways, leading to the expression of antioxidant genes that enhance the antioxidant capacity of infected liver cells, helping them resist ROS‐induced damage and survive [437]. However, HBV‐driven NRF2 activation also serves as an immune evasion strategy. Studies have shown that upregulation of NRF2‐regulated genes, such as NADPH quinone oxidoreductase 1 (NQO1), GSH‐Px, and glutamate cysteine ligase (GCLC), strengthens antioxidant defenses, modulates proteasomal activity, reduces viral antigen presentation, and diminishes T‐cell recognition, allowing HBV‐infected cells to survive immune surveillance, thereby promoting chronic infection and worsening liver disease [438, 439].

Similarly, HCV activates the NRF2/ARE pathway through viral proteins, including the core protein, NS3, NS4B, and NS5A, which activate signaling molecules such as PKC, casein kinase 2 (CK2), and PI3K. It induces NRF2 phosphorylation and dissociation from KEAP1, allowing NRF2 to translocate to the nucleus and promote antioxidant gene expression [287, 440]. However, HCV employs a nuanced strategy to regulate NRF2. While NRF2 is activated, the HCV core protein binds to small Maf (sMaf) proteins, preventing NRF2 from entering the nucleus, which limits excessive antioxidant protection. This controlled oxidative stress environment is beneficial for HCV replication and viral release [440].

In summary, NRF2 activation following HBV and HCV infection promotes liver cell proliferation and tumorigenesis by inhibiting apoptosis and enhancing survival signaling. The upregulation of NRF2‐regulated genes not only protects cells from oxidative stress but also improves metabolism and reduces ROS production, creating a tumor‐friendly environment. Additionally, viral proteins like HBx and NS5A further enhance NRF2 activity through PI3K/AKT and MAPK pathways, promoting cell proliferation and inhibiting apoptosis, which accelerates chronic liver inflammation and cell damage, ultimately leading to HCC.

#### 4.6.6 Hippo

The Hippo signaling pathway is an important pathway that regulates cell proliferation, apoptosis, and organ size [441]. In viral hepatitis, inactivation of the Hippo pathway leads to the nuclear translocation and activation of transcription factors, disrupting the balance between cell proliferation and apoptosis, contributing to malignant transformation, and activating tumorigenic genes that promote cell proliferation, migration, and survival [442].

HBV influences the Hippo/Yes‐associated protein (YAP)/transcriptional coactivator with PDZ‐binding motif (TAZ) pathway through multiple mechanisms, driving the malignant transformation of HCC. Studies have shown that HBx is closely associated with YAP activation in liver cancer. HBx activates YAP by upregulating the transcription factor forkhead box A1 (FOXA1) and the ubiquitin E3 ligase MSL2, which regulate histone H2B ubiquitination, enhancing YAP transcriptional activity [125]. Additionally, HBx interacts with the E3 ligase human double minute 2 (HDM2), preventing YAP ubiquitination and degradation, further stabilizing YAP in the nucleus [443]. Activated YAP induces oncogene expression, promoting hepatocyte proliferation and tumor progression [444]. Furthermore, in some HBV‐related HCC cases, HBV integrates into the host genome, particularly in the C‐terminal truncation of HBV surface proteins, which inhibits miR‐338‐3p expression, activates TAZ and increases tumor invasiveness and metastatic potential [445].

Likewise, HCV modulates the Hippo pathway to drive HCC malignancy. HCV NS5B protein inhibits Hippo signaling, activating YAP and upregulating Snail, which promotes EMT [446]. HCV E2 protein also regulates the Hippo pathway by mimicking the function of CD81, which is a significant regulator of the Hippo pathway and a significant ligand for glypican‐3 (GPC3). By reducing Hippo pathway activation, E2 decreases YAP inhibition, promoting cell proliferation and facilitating early tumor cell expansion, making HCV‐related HCC more aggressive [447, 448].

In conclusion, hepatitis viruses significantly contribute to the malignant transformation of HCC by modulating the Hippo–YAP/TAZ pathway, enhancing the aggressive nature of liver cancer.

#### 4.6.7 Wingless‐int/β‐Catenin

The Wnt/β‐catenin signaling pathway is essential for cell proliferation, differentiation, and survival, abnormal activation of which is closely linked to HCC development [449]. Both HBV and HCV modulate the Wnt/β‐catenin pathway through distinct mechanisms, promoting HCC onset and progression.

In HBV‐related HCC, the abnormal activation of the Wnt/β‐catenin pathway is a prominent driver of tumorigenesis. Studies have shown that mutations in the catenin beta1 (CTNNB1) gene, which encodes β‐catenin, are present in about 12% of HBV–HCC patients and directly cause abnormal activation of the Wnt/β‐catenin pathway [450]. In HBV‐infected livers, several regulators of the Wnt/β‐catenin pathway exhibit abnormal expression patterns. For example, Wnt2, Wnt7, and disheveled segment polarity protein 3 (DVL‐3) are upregulated in tumors, while negative regulators like naked cuticle homolog 1 (NKD1) and NKD2 are significantly downregulated, underscoring the crucial role of the Wnt pathway in HBV‐related tumor development [451]. Furthermore, HBx activates the Wnt/β‐catenin pathway via multiple mechanisms. HBx first suppresses E‐cadherin expression, disrupting cell adhesion, and promotes Wnt pathway activity via promoter hypermethylation and Src signaling activation [452]. HBx also directly binds to the tumor suppressor gene adenomatous polyposis coli (APC), preventing β‐catenin degradation, which causes β‐catenin accumulation in the cytoplasm and its translocation to the nucleus, where it activates Wnt target genes [453]. Inhibition of GSK‐3β degradation complex further accelerates the process, owing to persistent Wnt pathway activation [454].

HCV similarly promotes HCC by modulating the Wnt/β‐catenin pathway. In HCV‐related HCC patients, CTNNB1 mutations are found in approximately 26% of cases, significantly higher than the 12% observed in HBV–HCC [455]. HCV proteins like NS3 disrupt DNA repair mechanisms, increasing CTNNB1 mutation frequency and promoting Wnt/β‐catenin activation [456]. The HCV core protein, a major regulator of the Wnt/β‐catenin pathway, activates the pathway through modulation of nuclear transcription factors [457]. The core protein also enhances Wnt signaling by upregulating Frizzled (FZD) receptors and LDLR‐related proteins 5/6 (LRP5/6) while inhibiting antagonists like FZD‐related protein 2 and Dickkopf (DKK) [458]. In early HCV infection, DDK1 recruits DNA methyltransferases and HDACs, causing epigenetic silencing of gene promoters, which further drives β‐catenin activation [459]. Additionally, HCV NS5A protein interacts directly to GSK‐3β, preventing β‐catenin degradation and further enhancing Wnt pathway activation [460].

In conclusion, hepatitis viruses modulate the Wnt/β‐catenin pathway via multiple mechanisms, significantly promoting HCC malignant transformation. Abnormal Wnt/β‐catenin activation drives hepatocyte proliferation and metastasis, while fostering fibrosis and the tumor microenvironment, ultimately accelerating HCC onset and progression.

#### 4.6.8 p53

p53, often called the “guardian of the genome,” is a critical tumor suppressor protein activated in response to DNA damage or cellular stress, which regulates downstream genes to induce cell cycle arrest or apoptosis, preventing the proliferation of abnormal cells [461]. However, hepatitis viruses like HBV and HCV inhibit p53 activity through various mechanisms to evade immune surveillance, promote viral persistence, and contribute to HCC development.

In HBV infection, the viral HBx protein directly interferes with p53 function. HBx forms a complex with p53, preventing it from binding to DNA, thus blocking its role in DNA repair and apoptosis [462]. This inhibition mimics the behavior of mutated p53 in cancers, allowing the accumulation of DNA damage and promoting hepatocyte transformation into tumor cells [463, 464]. Murine double minute 2 (MDM2), a noteworthy negative regulator of p53, binds to p53 and facilitates its ubiquitination and degradation, inhibiting p53 tumor‐suppressing functions [465]. During HBV infection, HBx interacts with MDM2, promoting its nuclear translocation and further suppressing p53 transcriptional activity, which not only diminishes p53 anticancer functions but also stabilizes HBx, enhancing its carcinogenic effects [466]. Furthermore, MDM2 undergoes NEDDylation, increasing HBx stability and accelerating HBV‐induced liver fibrosis [443]. By disrupting the MDM2–p53 axis, HBV evades immune surveillance and promotes hepatocyte malignancy. MDM2 also interacts with the TGF‐β1 signaling pathway, contributing to liver fibrosis and tumorigenesis [467]. Beyond HBx, HBeAg suppresses p53‐dependent apoptosis pathways, such as the Fas/Fas Ligand (FasL) and TNF‐related apoptosis‐inducing ligand (TRAIL) pathways, enabling infected hepatocytes to evade immune clearance [468]. HBeAg interacts with NUMB endocytic adaptor protein (NUMB), weakening p53 transcriptional activity, making p53‐dependent apoptosis ineffective. Additionally, HBeAg accelerates p53 degradation via MDM2‐mediated ubiquitination, contributing to p53 inactivation. This dual mechanism allows HBV‐infected hepatocytes to survive longer, accumulate genetic damage, and eventually progress to liver cancer [469].

In like manner, HCV disrupts the p53 pathway to promote HCC development. HCV infection increases oxidative stress, inhibiting p53 acetylation and enhancing MDM2‐mediated p53 degradation [470]. The HCV core protein suppresses p53 transcriptional activity, reducing p53‐dependent apoptosis and helping infected hepatocytes evade cell death [471]. Furthermore, HCV activates the Nrf2 signaling pathway, promoting MDM2 expression and further disrupting the p53 pathway, enabling long‐term survival of infected hepatocytes and accumulation of damage that drives tumorigenesis [472].

Through these modes, both HBV and HCV disrupt p53 function, allowing infected hepatocytes to evade immune surveillance, accumulate mutations, and progress to HCC. The inactivation of p53 is a crucial step in the carcinogenesis associated with viral hepatitis.

#### 4.6.9 Vascular Endothelial Growth Factor

Angiogenesis is a critical process for growth and metastasis of HCC and is closely associated with VEGF [473]. Hepatitis viruses, such as HBV and HCV, promote HCC development by activating the VEGF signaling pathway, thereby enhancing angiogenesis.

In HBV‐related HCC, VEGF serves as a significant angiogenic factor. Studies have shown that VEGF and COX‐2 expression is significantly increased in HBV–HCC mice and patient tumor tissues, promoting an increase in microvascular density (MVD) [474]. MVD, a crucial marker of tumor angiogenesis, is strongly driven by the overexpression of VEGF, which stimulates the formation of new tumor blood vessels [474]. HBx, the HBV‐encoded protein, contributes to the process by stabilizing HIF‐α and activating mechanistic target of rapamycin (mTOR) and IκB kinase beta (IKKβ) signaling pathways, thereby enhancing VEGF expression [475]. Additionally, HBx activates the MAPK signaling pathway, increasing the secretion of angiopoietin‐2 (Ang‐2), further promoting angiogenesis in liver tissues [476]. Beyond VEGF stimulation, HBx upregulates MMPs, such as MMP‐2, MMP‐9, and MMP‐14, which degrade the ECM, facilitating endothelial cell migration and new blood vessel formation, thereby enhancing HCC angiogenic capacity [477].

In a comparable way, HCV promotes HCC progression by regulating angiogenesis pathways. The HCV core protein is closely linked to angiogenesis, as it activates various growth factor signaling pathways, including p38 MAPK, PI3K, and JNK, leading to increased VEGF and Ang‐2 expression, significantly enhancing angiogenesis in liver cancer [478, 479]. HCV core protein also activates the STAT3 signaling pathway, promoting VEGF expression, and interacts with androgen receptors to increase VEGF transcription [480]. Additionally, the proangiogenic effect of HCV core protein is dose‐dependent, with activation of the activator protein‐1 (AP‐1) pathway being another important mechanism driving increased VEGF expression [481].

By regulating the VEGF pathway, hepatitis viruses enhance angiogenesis, providing tumor with essential nutrients and oxygen, while also creating pathways for tumor cell dissemination and metastasis, which accelerates HCC progression.

#### 4.6.10 Insulin‐Like Growth Factor

The IGF signaling pathway, involving IGF‐1, IGF‐2, and their receptors (IGF‐1R and IGF‐2R), plays a crucial role in regulating cell proliferation, metabolism, and antiapoptotic processes [482]. Aberrant activation of the IGF signaling pathway is a key factor in the progression from viral hepatitis to HCC.

In HBV infection, which is strongly associated with HCC development, the IGF/IGF‐1R signaling pathway is activated. HBx upregulates IGF‐1R expression and increases IGF‐2 transcription by interacting with the Sp1 binding site on IGF‐2 promoter, which collectively lead to IGF‐2 overexpression and aberrant IGF signaling, accelerating liver cancer progression [483]. Additionally, HBV enhances IGF‐1R expression through the activation of HIF‐1α, further promoting HCC development. Notably, HIF‐1α activation under hypoxic conditions exacerbates IGF signaling, facilitating tumor growth and invasion [484].

Dysregulation of the IGF signaling pathway in chronic HCV infection, mediated by insulin resistance and chronic inflammation, drives HCC progression [485]. Insulin resistance and impaired growth hormone function, common in HCV‐infected patients, are associated with reduced IGF‐1 levels [486]. Low levels of IGF‐1 not only reduce proliferative capacity of liver cells but also heighten their sensitivity to inflammatory and tumor growth factors, thereby facilitating cancer cell formation and proliferation. IGF‐1 activates STAT5 signaling and induces EMT in HCC cells by downregulating E‐cadherin and upregulating N‐cadherin and vimentin [487].

The IGF signaling pathway not only promotes liver cancer cell proliferation but is also linked to CSCs characteristics. IGF‐1 treatment activates IGF/IGF‐1R signaling in HBV‐positive HCC cells, increasing expression of cancer stemness markers like nanog homeobox (NANOG) and octamer‐binding transcription factor 4 (OCT4). This suggests that IGF pathway activation maintains CSCs properties, promoting HCC recurrence and progression [488].

In conclusion, hepatitis viruses, particularly HBV and HCV, promote the malignant transformation of hepatocytes by regulating the IGF signaling pathway, particularly through upregulation of IGF‐2 and IGF‐1R. Aberrant activation of the IGF pathway not only exacerbates liver inflammation and metabolic dysregulation but also enhances CSCs self‐renewal, contributing to tumor invasiveness and recurrence risk.

## 5 Therapeutic Interventions

**FIGURE 4:**
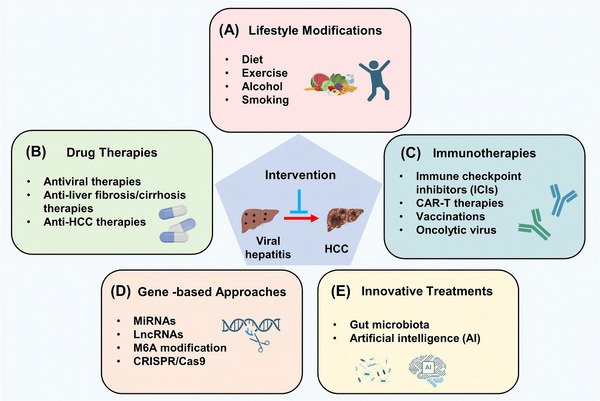
Interventions in the malignant transformation of viral hepatitis to HCC. Lifestyle modifications, drug therapies, immunotherapies, gene‐based approaches, and innovative treatments could inhibit the malignant transformation of viral hepatitis to HCC. CAR‐T, chimeric antigen receptor T‐cell; HCC, hepatocellular carcinoma; ICIs, immune checkpoint inhibitors; LncRNA, long noncoding RNA; M6A, N^6^‐methyladenosine; MiRNA, microRNA.

Therapeutic interventions for HBV/HCV‐associated HCC have evolved over the past decade. A healthy lifestyle effectively reduces the risk of HCC progression and enhances the quality of life for patients with viral hepatitis. Furthermore, as understanding of the pathogenesis of HBV/HCV‐related HCC deepens, advancements in drug therapies, immunotherapies, gene therapies, and other innovative treatments have broadened treatment options, making HCC management more precise and personalized (Figure 4).

### 5.1 Lifestyle Modifications

Recent studies have demonstrated that lifestyle changes, particularly in diet, smoking cessation, alcohol reduction, and exercise, can lower the risk of HCC progression in patients with viral hepatitis.

Diet is closely linked to the malignant progression of viral hepatitis into HCC. Excessive animal protein intake has been linked to increased liver damage in HBV‐infected mice. HBV transgenic mice on high‐casein diets showed a 5.1‐fold increase in serum HBsAg, while reducing casein intake to 6% limited this rise to 1.6‐fold, suggesting that moderating animal protein, especially dairy, helps mitigate liver damage and HCC progression in viral hepatitis patients [489]. Diets with a high glycemic index (GI) and glycemic load (GL) are also associated with HCC. Among HBV and HCV patients, high‐GL diets significantly raise HCC risk (RR = 1.52). Excessive dietary sugar can lead to insulin resistance and type 2 diabetes, which exacerbate liver inflammation and fibrosis. Thus, managing GL and selecting low‐GI foods help inhibit HCC progression [490]. Omega‐3‐rich diets, such as fish‐based diets, offer liver protection. A study in Japan involving 90,000 participants found that omega‐3 fatty acids reduced HCC incidence due to their anti‐inflammatory and antioxidant effects, which improve liver conditions in viral hepatitis patients [491]. Green tea, with its antioxidants, has shown similar benefits, with long‐term consumption linked to a 56% lower HCC risk in Chinese studies [492, 493]. Additionally, consuming garlic multiple times weekly may reduce HCC risk through bioactive compounds that decrease inflammation and inhibit cancer cell growth [492]. Smoking exacerbates HCC risk in HBV and HCV patients. Meta‐analyses show that smoking combined with HBV raises HCC risk by 19.81‐fold, and with HCV, by 24.86‐fold [494]. Tobacco components induce oxidative stress, promoting gene mutations and HCC formation while impairing NK cell function, reducing their ability to target HBV‐infected hepatocytes [495]. Smokers who quit experience a lowered HCC risk, with improved liver enzyme levels and decreased viral loads over time. Alcohol intake correlates with higher HCC incidence, particularly with daily consumption over 30 g. Alcohol‐induced liver inflammation and oxidative stress exacerbate HBV and HCV activity, leading to higher viral loads and mutation rates [496]. Reducing or abstaining from alcohol is crucial for preventing liver cancer progression. Regular physical activity also supports liver health. Studies have indicated that moderate exercise 3–4 times weekly can reduce liver cancer risk by over 30% [497]. Proper exercise and weight management can limit hepatitis severity and progression to cirrhosis and HCC.

In summary, lifestyle improvements, including a balanced diet, regular exercise, and avoiding smoking and alcohol, effectively reduce the risk of HCC progression and enhance quality of life for viral hepatitis patients. For high‐risk individuals with chronic HBV or HCV, these lifestyle modifications are critical for preventing HCC.

### 5.2 Drug Therapies

During the malignant transformation from viral hepatitis to HCC, drugs intervene by inhibiting progression through various mechanisms at different disease stages, offering therapeutic benefits (Table 3).

**TABLE 3:** Therapeutic applications for HBV, HCV, liver fibrosis/cirrhosis, and HCC.

Disease	Classification	Agents	Targets/mechanism	Application/stage	References
HBV	IFN	PEG–IFN	Inhibits HBV replication and regulates antiviral immune responses	Approved	[498]
	NA	ETV	HBV polymerase inhibitor	Approved	[499]
		TDF	HBV polymerase inhibitor	Approved	[499]
		TAF	HBV polymerase inhibitor	Approved	[499]
HCV	DAA	Glecaprevir	NS3/4A inhibitor	Approved	[500]
		Grazoprevir	NS3/4A inhibitor	Approved	[501]
		Paritaprevir	NS3/4A inhibitor	Approved	[502]
		Simeprevir	NS3/4A inhibitor	Approved	[503]
		Voxilaprevir	NS3/4A inhibitor	Approved	[504]
		Daclatasvir	NS5A inhibitor	Approved	[505]
		Pibrentasvir	NS5A inhibitor	Approved	[500]
		Elbasvir	NS5A inhibitor	Approved	[501]
		Ledipasvir	NS5A inhibitor	Approved	[506]
		Ombitasvir	NS5A inhibitor	Approved	[502]
		Velpatasvir	NS5A inhibitor	Approved	[504]
		Sofosbuvir	NS5B inhibitor	Approved	[504]
Liver fibrosis/cirrhosis	Natural product	Silymarin combination with sofosbuvir and ribavirin	Ameliorates oxidative stress, diminish latent viral load, revamp the fluster level of sex hormones	Miniature clinical trial	[507]
		Caffeine	Blocks the TGF‐β/Smad3 and TLR4/MAPK/NF‐kB signaling pathway	Nationwide cross‐sectional study	[508, 509, 510]
		Berberine	Induces ferrous redox to activate ROS‐mediated HSC ferroptosis, regulates lipid metabolism and intestinal flora	Preclinical research	[511, 512]
		Curcumin	Inhibits oxidative stress by activating PPAR‐α and regulates AMPK and PI3K/AKT/mTOR pathway to increase the autophagic flow in hepatocytes	Preclinical research	[513]
		Glycyrrhizic acid	Promotes CUGBP1‐mediated IFN‐γ/STAT1/Smad7 signalling pathways	Preclinical research	[514]
		Physalin D	Inhibits the TGF‐β/Smad and YAP signaling pathways	Preclinical research	[515]
		Demethylzeylasteral	Inhibits the AGAP2 mediated FAK/AKT signaling axis	Preclinical research	[516]
		Schizandrin C	Regulate lipid metabolism and inflammation by NF‐kB and p38/ERK MAPK signaling pathways	Preclinical research	[517]
	MSC	UC‐MSC	Improves long‐term survival rates and liver function in patients with HBV‐related decompensated liver cirrhosis	Randomized controlled trial	[518]
		Autologous MSC	Exhibits a supportive role in the treatment of end‐stage liver disease with beneficial effects on liver synthetic functions and hepatic fibrosis	Randomized controlled trial	[519]
		hUC‐MSC	Regulates mitochondrial function through Sirt3	Preclinical research	[520]
		MSC‐ex	Downregulates YAP/LOXL2 pathway and inhibits Th17 differentiation	Preclinical research	[521, 522]
		BM‐MSC	Promotes Ly6C^high^/Ly6C^low^ subset conversion and Ly6C^low^ macrophage restoration through secreting antifibrogenic–cytokines and activating the apoptotic pathway	Preclinical research	[523]
		T‐MSCs	Inactivates HSCs by suppressing hedgehog signaling	Preclinical research	[524]
HCC	Targeted drug	Sorafenib	RAF, VEGFR1‐2, PDGFR‐β, KIT RET, and KIT	First‐line	[525]
		Lenvatinib	VEGFR1‐2, FGFR, PDGFR, RET, and KIT	First‐line	[526]
		Bevacizumab	VEGF‐A	First‐line	[527]
		Donafenib	VEGFR, PDGFR, and RAF	First‐line	[528]
		Regorafenib	VEGFR1‐2, FGFR, PDGFR, RAF, BRAF, and KIT	Second‐line	[529]
		Cabozantinib	VEGRF1‐2, RET, MET, and AXL	Second‐line	[530]
		Ramucirumab	VEGFR‐2	Second‐line	[531]
		Apatinib	VEGFR‐2	Second‐line	[532]
	ICI	Tremelimumab	CTLA‐4	Phase III trial	[533]
		Ipilimumab	CTLA‐4	Phase I/II trial	[534]
		Nivolumab	PD‐1	Phase I/II trial	[534]
		Pembrolizumab	PD‐1	Phase III trial	[535]
		Tislelizumab	PD‐1	Phase III trial	[536]
		Atezolizumab	PD‐L1	Phase III trial	[537]
		Durvalumab	PD‐L1	Phase III trial	[533]

Abbreviations: AGAP2, Arf GTPase‐activating protein 2; AKT, protein kinase B; AMPK, AMP‐activated protein kinase; AXL, AXL receptor tyrosine kinase; BM‐MSC, bone marrow‐mesenchymal stem cells; BRAF, B‐Raf; CTLA‐4, cytotoxic T‐lymphocyte‐associated protein 4; CUGBP1, CUGBP Elav‐like family member 1; ERK, extracellular signal‐regulated kinase; ETV, entecavir; FAK, focal adhesion kinase; FGFR, fibroblast growth factor receptor; HBV, hepatitis B virus; HCC, hepatocellular carcinoma; HCV, hepatitis C virus; HSC, hepatic stellate cell; HSCs, hepatic stellate cells; ICI, immune checkpoint inhibitor; IFN, interferon; IFN‐γ, interferon gamma; KIT, KIT proto‐oncogene, receptor tyrosine kinase; LOXL2, lysyl oxidase like 2; Ly6C, lymphocyte antigen 6 family member C; MAPK, mitogen‐activated protein kinase; MET, mesenchymal–epithelial transition; MSC, mesenchymal stem cell; MSC‐ex, mesenchymal stem cell‐derived exosomes; mTOR, mammalian target of rapamycin; NA, nucleoside analogue; NF‐κB, nuclear factor kappa B; NS3/4A, nonstructural protein 3/4A; NS5A, nonstructural protein 5A; p38, p38 mitogen‐activated protein kinase; PD‐1, programmed death‐1; PDGFR‐β, platelet‐derived growth factor receptor beta; PD‐L1, programmed death‐ligand 1; PEG–IFN, polyethylene glycol–interferon; PI3K, phosphoinositol‐3 kinase; PPAR‐α, peroxisome proliferator‐activated receptor alpha; RAF, rapidly accelerated fibrosarcoma; RET, REarranged during transfection; ROS, reactive oxygen species; SIRT3, sirtuin 3; SMAD3, SMAD family member 3; SMAD7, SMAD family member 7; STAT1, signal transducer and activator of transcription 1; TAF, tenofovir alafenamide; TDF, tenofovir disoproxil fumarate; TGF‐β, transforming growth factor beta; Th17, T helper cell 17; TLR4, toll‐like receptor 4; T‐MSCs, tissue‐derived mesenchymal stem cells; UC‐MSC, umbilical cord mesenchymal stem cell; VEGF‐A, vascular endothelial growth factor A; VEGFR1‐2, vascular endothelial growth factor receptor 1–2; YAP, Yes1‐associated transcriptional regulator.

#### 5.2.1 Antiviral Therapies

Chronic infections with HBV or HCV are well‐recognized primary risk factors for HCC development through multiple mechanisms. Consequently, achieving sustained viral suppression or eradication has proven effective strategy in preventing HCC. Over the last decade, advancements in antiviral agents, including IFN, NAs, and DAAs, have enabled sustained viral suppression or cure in most chronic HBV/HCV patients, significantly impacting HCC prevention (Table 3).

##### 5.2.1.1 Hepatitis B Virus

The recommended treatment for CHB includes IFNs and NAs, such as entecavir (ETV), tenofovir disoproxil fumarate (TDF), and tenofovir alafenamide fumarate (TAF), which reduce hepatic inflammation by suppressing viral replication [538]. IFNs are cytokines generated from immune cells in response to viral infections. Compared with NAs, IFNs offer the benefits of a finite treatment duration, the absence of viral resistance, and a higher likelihood of achieving SVR, along with the potential for sustained HBsAg loss and anti‐HBs seroconversion [539]. Long‐term follow‐up studies of PEGylated IFN (PEG–IFN) treatment have demonstrated that nearly half of the patients achieved a sustained response without further need for NAs, resulting in favorable outcomes such as HBsAg loss and absence of disease progression [540]. Studies on immune mechanisms have suggested that IFN‐γ^+^ Th1 cells activate intrahepatic resident memory T cells, promoting HBsAg loss via M1 macrophage polarization [541]. Meta‐analyses have indicated that finite‐duration IFN therapy improves survival in HBV‐related HCC postcurative surgery patients, ultimately enhancing overall survival (OS) [542]. Although IFN has a higher cure rate, it is frequently accompanied by significant adverse effects, including cytopenia, neuropsychiatric exacerbations, and increased production of thyroid autoantibodies, which can hinder patient tolerance [543].

NAs inhibit DNA polymerase, an enzyme essential for HBV replication in hepatocytes, thereby suppressing viral replication and exerting antiviral effects. According to the American and European Societies for the Study of the Liver, the objective of NA therapy is to achieve permanent clearance of HBsAg, with or without seroconversion after treatment [544]. However, NAs are unable to eradicate cccDNA or integrated HBV DNA sequences, leading to limited rates of serum HBsAg clearance [545]. Nonetheless, the role of NAs in reducing HCC risk remains significant. Over the past decade, an increasing number of patients with HBV‐related HCC have received antiviral treatment, with evidence suggesting that NAs therapy improves survival whether initiated before or after HCC diagnosis [546]. Timely and appropriate preoperative NAs therapy also enhances the prognosis of HBV‐related HCC, even with normal ALT levels and negative HBeAg [547]. Notably, ETV and TDF are associated with a significantly lower HCC risk compared with other NAs [548], underscoring their critical role in preventing the progression of HBV to HCC.

##### 5.2.1.2 Hepatitis C Virus

The primary goal of HCV therapy is to achieve SVR, defined as undetectable HCV–RNA. Achieving SVR is associated with normalization of transaminase levels, reduction in liver necro‐inflammation and fibrosis, improved liver function, and reduced HCC risk [549]. The approval of DAAs revolutionized HCV treatment, enabling cure in nearly all patients [550]. Several DAAs approved by the European Medicines Agency (EMA) and the United States Food and Drug Administration (US FDA) are classified into three main groups based on their HCV protein targets: NS3/4A protease inhibitors that block HCV polyprotein processing (e.g., glecaprevir, grazoprevir, simeprevir, paritaprevir, voxilaprevir); NS5A inhibitors that disrupt viral replication and assembly (e.g., daclatasvir, elbasvir, ledipasvir, ombitasvir, pribrentasvir, velpatasvir); and NS5B polymerase inhibitors that block HCV RNA replication (e.g., nucleoside inhibitor sofosbuvir, non‐nucleoside inhibitor dasabuvir) [551].

DAA treatment has also been shown to reduce the risk of HCC occurrence. A nationwide, multicenter retrospective cohort study in South Korea has investigated the impact of DAA on disease burden in patients with HCV infection. Compared with untreated group, DAA‐treated group exhibited a significantly reduced risk of HCC, liver decompensation, and mortality, with hazard ratios of 0.41, 0.31, and 0.22, respectively [552]. Additionally, Liu et al. conducted a prospective study on changes in liver stiffness (LS) and HCC occurrence following HCV eradication with DAA treatment over 3 years. Matched analysis revealed that LS reduction was significantly greater in the DAA group than in the PEG–IFN group, indicating more pronounced fibrosis regression [553].

#### 5.2.2 Antiliver Fibrosis/Cirrhosis Therapies

Chronic liver injury caused by HBV and HCV leads to disease progression, from hepatocyte injury to fibrosis, cirrhosis, and ultimately HCC. Although no therapy approved by the US FDA or EMA directly target liver fibrosis or cirrhosis, several promising agents have shown success in reversing liver fibrosis in clinical/preclinical trials (Table 3) [554].

Antiviral therapy targeting the underlying infection has made significant progress in preventing and even reversing liver fibrosis progression. In CHB patients with hepatic steatosis, antiviral therapy was independently associated with a lower risk of fibrosis progression [555]. Additionally, a prospective study of HCV patients treated with DAAs found that HCV clearance improved liver function and fibrosis [556]. Further research has underscored the significance of chemokine‐related proinflammatory pathways in the immunopathology of liver fibrosis. Antiviral treatment has been shown to reduce CXCL9, CXCL10, and CXCL11 levels, thereby mitigating the severity of liver cirrhosis [557].

Furthermore, anti‐inflammatory, hepatoprotective, and antifibrotic agents address hepatitis virus‐related‐fibrosis symptoms and play distinct roles at different disease stages. Silymarin, a flavonoid compound, protects healthy and partially damaged liver cells by reducing oxidative stress and mitigating cytotoxicity. It has been approved in several countries for treating liver conditions [558]. In a small clinical trial, silymarin adjunct therapy enhanced DAA efficacy, normalizing hepatological, serological, hormonal, and antioxidant markers in HCV patients [507]. Berberine has also been investigated as a potential antifibrotic agent, with mechanisms including the activation of ROS‐mediated HSC ferroptosis [511], inhibition of inflammation via a sirtuin 3‐dependent mechanism [559], and regulation of lipid metabolism and gut microbiota to improve liver health [512].

Beyond traditional drug therapies, mesenchymal stem cells (MSCs) have emerged as a novel treatment for liver fibrosis and cirrhosis. They can migrate to injury sites, inhibit HSC activation, differentiate into hepatocytes, and participate in immune regulation, thereby reducing ECM deposition and ameliorating hepatic fibrosis [560]. In a randomized controlled trial, umbilical cord‐derived MSC treatment significantly improved long‐term survival rates and liver function in patients with decompensated cirrhosis induced by HBV, introducing a novel therapeutic approach [518]. Additionally, autologous MSC infusion via peripheral veins has shown promise in treating patients with HCV and end‐stage liver disease, demonstrating satisfactory effects on liver synthetic function and fibrosis [519]. However, due to the complex mechanisms involved in liver fibrosis formation and significant individual variations, antifibrotic drug development continues to face considerable challenges.

#### 5.2.3 Anti‐HCC Therapies

Surgical resection, liver transplantation (LT), and local ablative therapy are the primary treatments for HCC [561]. LT is the preferred treatment for early‐stage HCC patients who are not candidates for resection due to hepatic dysfunction or multifocal neoplasms, as it addresses both HCC and the underlying liver pathophysiology. For patients with minimal tumor load and no vascular invasion or metastatic spread, transcatheter arterial chemoembolization (TACE) is the frontline therapy [561]. Due to its aggressive nature and potential for asymptomatic progression, many patients are diagnosed at intermediate or advanced stages, limiting the opportunity for optimal intervention. Conventional therapeutic approaches have demonstrated suboptimal efficacy in advanced HCC, prompting a paradigm shift toward the utilization of systemic therapeutics.

Targeted therapies have introduced new treatment approaches for hepatitis virus‐associated HCC, particularly in advanced‐stage patients. Sorafenib remains the primary first‐line therapy, followed by lenvatinib, bevacizumab (BVZ), and donafenib as additional first‐line options. Sorafenib, a multikinase inhibitor, exerts antineoplastic effects by inhibiting VEGF receptor (VEGFR), platelet‐derived growth factor receptor‐beta (PDGFR‐β), and Raf/MEK/ERK signaling cascades, thereby inhibiting tumor proliferation and angiogenesis [525]. In 2018, lenvatinib, a multikinase inhibitor with a spectrum of activity against VEGFR, FGF receptor (FGFR), PDGFR, rearranged during transfection (RET), and stem cell factor receptor (KIT), was approved by the US FDA and China's NMPA as a frontline therapeutic option for patients afflicted with unresectable HCC [526]. BVZ, a recombinant IgG1 monoclonal antibody, demonstrates high‐affinity binding to VEGF and thereby preventing VEGF–VEGFR interaction [562]. The combination regimen of atezolizumab with BVZ has been established as the standard first‐line treatment protocol for patients with unresectable or metastatic HCC [563]. Donafenib, an independently developed molecular‐targeted drug in China and a derivative of chemically modified sorafenib, targets receptor tyrosine kinases (RTKs), including VEGFR and PDGFR, and directly inhibits Raf kinases and Raf/MEK/ERK pathways. The mechanism of anti‐HCC is to inhibit tumor cell proliferation and neovascularization, exerting antitumor effects through multitarget blockade (Table 3) [528].

Resistance to first‐line targeted therapy has necessitated the expansion of second‐line treatment options for HCC. Regorafenib, a multitarget kinase inhibitor structurally sharing structural homology with sorafenib, exerts its therapeutic effects by targeting VEGFR, FGFR‐1, PDGFR, RAF, KIT, and RET, thereby inhibiting angiogenesis and modulating tumor microenvironment, which was approved by the US FDA in 2017 for HCC patients who have demonstrated refractoriness to sorafenib [529]. Cabozantinib is also a multitarget small molecule tyrosine kinase inhibitor (TKI) that inhibits mesenchymal‐epithelial transition (MET), VEGFR, ROS proto‐oncogene 1 (ROS1), AXL receptor tyrosine kinase (AXL), neurotrophic tyrosine receptor kinase (NTRK), KIT, RET, and was approved by the US FDA in 2019 for advanced HCC [530]. Ramucirumab, an IgG1 monoclonal antibody, binds to VEGFR‐2 thereby inhibiting angiogenesis. The US FDA approved ramucirumab in 2019 for advanced HCC patients with AFP levels ≥400 ng/mL [531]. Apatinib, a TKI selectively inhibiting of VEGFR‐2 and blocking of VEGF signaling, has also been approved for advanced HCC (Table 3) [532].

In summary, antiviral treatments, including IFN, NAs and DAAs, have proven effective strategies in preventing HCC by achieving sustained viral suppression and eradication. Several promising agents such as silymarin and berberine, as well as MSCs therapy, have demonstrated potential in ameliorating liver fibrosis caused by HBV and HCV. At the HCC stage, targeted therapies such as sorafenib have successfully inhibited tumor proliferation and angiogenesis. However, side effects and patient tolerance may limit the clinical use of these drugs, highlighting the need for new targets and therapies to improve efficacy.

### 5.3 Immunotherapies

Hepatocytes create a tolerogenic immune environment, where both innate and adaptive immunity are crucial in preventing permanent hepatic injury [564]. Consequently, the modulation of immune responses represents a promising therapeutic avenue for prevention of HCC progression, particularly in the context of HCC etiology linked to viral hepatitis (Table 4).

**TABLE 4:** Interventions for immune system.

Classification	Agents	Effects	Stage	References
CAR‐T	GPC3–CAR‐T	Reverses tumor immunosuppressive microenvironment by reducing PMN–MDSC and Treg cell infiltration	Phase I trial	[565, 566]
	CD133‐directed CAR‐T	Demonstrates promising antitumor activity and a manageable safety profile	Phase II trial	[567]
	GPC3/PD‐1 CART‐T	Limits PD‑1‑PD‑L1 binding and sustains cytotoxicity to PD‑L1 HCC cells	Preclinical research	[568]
	CD39 HBV–CAR‐T	Increases the secretion of IFN‐γ	Preclinical research	[569]
	DLK1 directed CAR‐T	Enhances T cell proliferation and activation in a DLK1‐dependent manner	Preclinical research	[570]
Vaccination	PTCV	Induces antitumor T cells and has clinical activity in advanced HCC	Phase I/II trial	[571]
	Novel therapeutic vaccine	Induces CD8^+^ T cells infiltration into tumors	Phase I trial	[572]
	Neoantigen‐based DC vaccine	Induces antitumor responses against recurrence of HCC	Phase II trial	[573]
	HepaVac‐101	Demonstrates a good safety profile and immunogenicity in HCC	Phase I/II trial	[574]
	GPC3 vaccine	Improves long prognosis of HCC	Phase II trial	[575]
	Personalized neoantigen vaccine	Prevents postoperative recurrence in HCC patients	Clinical trial	[576]
	OVA–aPD1 NMP	Induces a strong CD8^+^ memory T cells response	Preclinical research	[577]
	LDHs–cGAMP–nanovaccine	Promotes the response efficiency of αPD‐L1 immunotherapy	Preclinical research	[578]
	Neoantigen vaccine	Increases tumor‐reactive immune responses	Preclinical research	[579]
	Poly‐ICLC	Increases infiltration of tumor‐specific CD8^+^ T cells and induces CD8^+^ T cell‐dependent inhibition of tumor growth	Preclinical research	[580]
	DCs‐based neoantigen nano‐vaccine	Induces the activation and proliferation of neoantigen‐specific T cells to suppress the primary/distal tumor growth	Preclinical research	[581]
OV	OV H101	Reverses ICI resistance and demonstrates efficacy in treating refractory advanced HCC	Clinical trial	[582]
	VSV–IFNβ	Generates antitumor T cell populations upon which ICIs can effectively work	Preclinical research	[583]
	OV with deINS1–GM‐CSF	Activates CD4^+^ and CD8^+^ T cells through the JAK2–STAT3 pathway	Preclinical research	[584]
	OV carrying GV1001	Increases the number of CD4^+^ and CD8^+^ T cells and improves survival	Preclinical research	[585]
	OV expressing rgFlu/PD‐L1	Activates the cGas–STING pathway in CD8^+^ T cells to kill HCC cells	Preclinical research	[586]
	WNV‐poly(A)	Activates DCs and trigger tumor antigen specific response mediated by CD8^+^ T cell	Preclinical research	[587]

Abbreviations: aPD1, alpha programmed death‐1; CAR‐T, chimeric antigen receptor T‐cell; CD133, cluster of differentiation 133; CD39, cluster of differentiation 39; cGAMP, cyclic GMP–AMP synthase; cGAS, cyclic GMP–AMP synthase; DC, dendritic cell; delINS1, deleted NS1; DLK1, delta‐like canonical Notch ligand 1; GM‐CSF, granulocyte‐macrophage colony‐stimulating factor; GPC3, glypican‐3; GV1001, a 16‐amino‐acid peptide comprising a sequence from the human enzyme telomerase reverse transcriptase; HBV, hepatitis B virus; HCC, hepatocellular carcinoma; ICI, immune checkpoint inhibitor; IFN‐γ, interferon gamma; JAK2, Janus kinase2; LDHs, lactate dehydrogenases; NMP, microsphere and nanosphere particle; OV, oncolytic virus; OVA, ovalbumin; PD‐1, programmed death‐1; PD‐L1, programmed death‐ligand 1; PMN‐MDSC, polymorphonuclear myeloid‐derived suppressor cells; Poly‐ICLC, polyinosinic‐cytidylic acid and poly‐L‐lysine carbohydrate; PTCV, peritumoral T cell infiltration; rgFlu, recombinant influenza virus; STAT3, signal transducer and activator of transcription 3; STING, stimulator of interferon genes; VSV‐IFN, vesicular stomatitis virus, expressing interferon; WNV‐poly(A), West Nile virus‐poly(A); αPD‐L1, alpha programmed death‐ligand 1.

#### 5.3.1 Immune Checkpoint Inhibitors

Virus‐infected liver cells and HCC cells evade immunosurveillance by upregulating PD‐L1 expression on their surface, which interacts with T‐cell immune checkpoint receptors, including B‐ and T‐lymphocyte attenuator (BTLA), PD‐1, and CTLA‐4, inhibiting T‐cell activation and immune responses, enabling malignant cells to escape immune detection, proliferate uncontrollably, and accelerating the progression of viral hepatitis to HCC [588]. Immune checkpoint inhibitors (ICIs) reshape the tumor microenvironment by activating suppressed immune cells to attack the tumor. US FDA‐approved ICIs include agents targeting PD‐L1 (atezolizumab, durvalumab), PD‐1 (pembrolizumab, nivolumab), and CTLA‐4 (ipilimumab, tremelimumab). By disrupting immune checkpoints, ICIs relieve tumor‐induced T‐cell inhibition, restoring their activation and cytotoxic function.

Two pivotal clinical trials, IMbrave150 and HIMALAYA, have demonstrated a significant enhancement in overall and progression‐free survival for patients with unresectable HCC treated with ICIs compared with sorafenib [589, 590]. In the IMbrave150 trial, the combination of atezolizumab and BVZ exhibited superior efficacy over sorafenib, with improved median OS (19.2 vs. 13.4 months), median progression‐free survival (6.8 vs. 4.3 months), and objective response rates (30 vs. 11%) [589]. The HIMALAYA trial investigated the synergistic effect of two ICIs: durvalumab and tremelimumab. The STRIDE (single tremelimumab regular interval durvalumab) treatment, based on phase II data, demonstrated superior OS compared with sorafenib (median 16.4 vs. 13.8 months) and a higher objective response rate (20.1 vs. 5.1%) [590].

Meta‐analyses have indicated that HCC patients with viral hepatitis achieve favorable survival outcomes with ICIs treatments [591]. Notably, ICIs have the potential to rejuvenate exhausted T‐cell immunity, which may facilitate not only the treatment of cancer but also potentially lead to the cure of CHB. In HCC patients receiving ICIs, a functional cure of hepatitis B has been observed, with a higher cumulative incidence of HBsAg loss, particularly in patients with baseline HBsAg levels <100 IU/mL [592].

While ICIs have emerged as valuable treatment options for solid tumors, further research is warranted to confirm their suitability as a primary treatment for HCC. However, not all patients benefit from immunotherapies. As more data become available, personalized treatment for HCC is likely to become the prevailing approach.

#### 5.3.2 Chimeric Antigen Receptor‐T Cell Therapy

Chimeric antigen receptor (CAR)‐T cell therapy is a novel immunotherapeutic modality that targets and eliminates virus infected liver cells and HCC cells. The CAR is a recombinant protein engineered to bind specific tumor‐associated antigens, independent of MHC restriction, enabling T cells to recognize and eliminate malignant cells expressing the designated antigen [593]. The application of CAR‐T cell therapy to HCC necessitates the identification of antigens that are selectively expressed on the surface of cancer cells [594]. GPC3, a membrane‐anchored member of the HSPG family, is specifically upregulated in HBV/HCV‐associated HCC, making it a compelling therapeutic target. Experimental evidence suggests that GPC3‐targeted CAR‐T cells are capable of inducing sustained tumor regression in murine models of HCC [595]. Phase I clinical trials have documented the antitumor activity of CAR–GPC3 T cells in HCC patients, thereby establishing the feasibility and preliminary safety of treatment [565]. Recently, a dual‐target CAR‐T cell therapy that targets both GPC3 and PD‐1 has been developed, demonstrating the ability to eliminate PD‐L1‐positive HCC tumor cells and modulate the immunosuppressive tumor microenvironment, thereby exhibiting enhanced tumor inhibition compared with single‐target CAR‐T cells [568].

Recent studies have also concentrated on engineered T cells targeting the hepatitis virus genome or proteins for treating viral hepatitis‐induced HCC. Tan et al. identified that HBV‐related HCC tumor cells express antigenic epitopes translated from integrated HBV mRNAs, which can activate T cells. Therefore, autologous T cells engineered to express T‐cell receptors (TCRs) specific for HBV‐DNA epitopes were adoptively transferred to two HCC patients with posttransplant recurrence [596]. For chronic HCV infection, a CAR‐T therapy targeting the HCV/E2 glycoprotein has been developed to secrete antiviral and proinflammatory cytokines while lysing HCV‐infected hepatocytes, thereby controlling HCV infection [597]. However, no clinical trials have been conducted to date for HCV‐targeting CAR‐T therapy, and its efficacy in treating HCV‐related HCC remains undetermined.

#### 5.3.3 Vaccinations

Vaccines are essential for preventing and treating hepatitis virus‐induced HCC, functioning in two primary categories: preventive and therapeutic. Prophylactic hepatitis B vaccines induce neutralizing antibodies against HBV envelope proteins, preventing hepatitis B and reducing both HBV infection and HCC incidence [598]. However, the development of effective prophylactic hepatitis B vaccines is complicated by viral hypervariability and immune evasion mechanisms [599]. Therapeutic vaccines are essential for HCC treatment as they aim to elicit a robust T‐cell response that enhances the liver's immune microenvironment and inhibits the progression from viral hepatitis to HCC.

In a phase II trial with GPC3 peptide vaccination for HCC, the vaccine reduced 1‐year recurrence by 15% and improved 5‐ and 8‐year survival rates by 10 and 30%, respectively. The vaccine demonstrated greater efficacy in HCC patients with elevated GPC3 expression, highlighting the effectiveness of cancer vaccination in specific HCC subgroups [575]. In another phase II study focused on HBV infection, the GS‐4774 vaccine was shown to enhance the production of IFN, TNF, and IL‐2 by CD8^+^ T cells exposed to antigenic peptides, thereby disrupting immune tolerance in HBV‐infected individuals [600]. In a phase I/II multicenter trial for HCC, HepaVac‐101, which combines multipeptide antigen IMA970A with the TLR7/8 RIG‐I agonist CV8102, demonstrated a favorable safety profile and elicited immune responses against tumor‐associated antigens (TAAs) [574].

In addition to peptide‐based vaccines, RNA‐based vaccines have also been developed for therapeutic intervention in HCC. Specifically, poly‐ICLC, a synthetic double‐stranded RNA molecule designed to emulate viral infection, has demonstrated potential in stimulating immune responses. Studies have indicated that a combinatorial therapeutic approach, involving initial intratumoral administration followed by intramuscular injections of poly‐ICLC, significantly suppressed tumor growth and enhanced CD8^+^ T cell infiltration [580]. Furthermore, combining therapeutic vaccines with ICIs has also demonstrated potential in HCC treatment. In a phase I/II trial, a personalized therapeutic cancer vaccine coadministered with plasmid‐encoded IL‐12 and pembrolizumab, exhibited clinical activity in advanced HCC patients by inducing antitumor T cell responses [571].

Serum levels of AFP are commonly elevated in most HCC tumors and can serve as a predictor of HCC recurrence in patients with viral hepatitis [601]. A study by Lu et al. demonstrated that AFP‐based vaccine immunization combined with anti‐PD‐L1 treatment significantly inhibited HCC progression in AFP‐positive tumor models [602]. Additionally, poly(lactic‐coglycolic acid (PLGA)) micro/nanoparticle vaccination combined with anti‐PD1 antibodies and ovalbumin (OVA) has been shown to enhance the activation and proliferation of OVA‐specific CD8^+^ T cells, suggesting its potential to augment antitumor immune responses in HCC [577]. An advanced in situ nanovaccine, utilizing layered double hydroxides as a vehicle for cyclic GMP‐AMP (cGAMP), a stimulator of IFN genes (STING) agonist, and adsorbed TAAs, has been reported to significantly modulate tumor immune microenvironment and augment the efficacy of anti‐PD‐L1 immunotherapies for HCC, offering a promising strategy for in situ cancer vaccination [578].

These vaccination strategies have demonstrated safety profiles, with the majority inducing antigen‐specific responses without toxic or autoimmune reactions. However, the immunosuppressive environment of HCC presents challenges for eliciting effective immune responses, and the heterogeneous expression of TAAs in HCC tumors may limit the effectiveness of vaccines and increase the potential for immune evasion [603]. Consequently, enhancing vaccine efficacy against HCC malignancy requires a deeper understanding of TAAs and the strategic integration of vaccination with immune‐activating approaches.

#### 5.3.4 Oncolytic Virus

The immunosuppressive tumor microenvironment in viral hepatitis‐related HCC may constrain the therapeutic efficacy of ICIs [604]. Oncolytic virus (OV) therapy is a novel immunotherapeutic approach that selectively targets and eliminates tumor cells, inducing tumor lysis and antitumor immune responses [605]. For instance, the oncolytic influenza virus delNS1‐ granulocyte‐macrophage colony‐stimulating factor (GM‐CSF), when combined with PD‐1 blockade therapy, has been shown to effectively target and eliminate HCC cells without impacting normal hepatocytes by activating CD4^+^ and CD8^+^ T cells through the JAK2–STAT3 pathway [584]. Comprehending the immune microenvironment in HCC is essential for elucidating the effects of OV. Spatial transcriptomics and single‐cell RNA sequencing revealed that SynOV treatment increases CD8^+^ T cells infiltration, enhances Cxcl9–Cxcr3‐mediated communication, and normalizes Kupffer cells in the tumor microenvironment. The tumor‐reducing effects of SynOV have been clinically observed in metastatic HCC patients during a phase I clinical trial [606]. Ideally, OVs should selectively replicate in malignant cells to reverse the immunosuppressive microenvironment and be cleared by the host's immune system. The live attenuated vaccine West Nile virus (WNV)‐poly(A) has emerged as a promising oncolytic agent. Mechanistically, it selectively targets and kills tumor cells while sparing normal cells due to its high sensitivity to IFN. Furthermore, WNV‐poly(A) activates DCs, triggering a tumor antigen‐specific response mediated by CD8^+^ T cells, thereby inhibiting the growth of both primary and metastatic tumor cells [587].

To avert host antiviral responses and achieve tumor‐selective targeting, a surface engineering strategy has been devised to mask oncolytic herpes simplex virus (oHSV) with a galactose–polyethylene–glycol (PEG) polymer chain. The glycosylated‐PEG–oHSV mitigates the immunosuppressive tumor microenvironment by augmenting the infiltration of CD8^+^ T cells and NK cells, stimulating the secretion of antitumor cytokines, ultimately impeding HCC progression [607]. A novel recombinant OV has also been constructed by incorporating NA fragments and GV1001 peptides, which exhibits selective cytotoxicity toward tumor cells. In alignment with in vitro observations, the recombinant OV significantly suppresses liver tumor growth and induces an antitumor immune response characterized by increased CD4^+^ and CD8^+^ T cells, thereby enhancing survival rates [585]. Recently, a recombinant Sindbis virus carrying GM‐CSF has been developed for HCC therapy [608]. Studies have demonstrated that Sindbis virus effectively infects HCC cell lines and induces cell death, with the addition of GM‐CSF potentiating its tumoricidal effects and augmenting immune cell infiltration in the tumor microenvironment.

Although OVs have demonstrated potential in the therapeutic management of cancer and preliminary research has yielded promising findings, the absence of substantive clinical data creates a significant void in the comprehensive assessment of their efficacy in the context of hepatic malignancies. Addressing this gap between basic research and clinical application is essential for validating OVs as a viable treatment for patients with viral hepatitis‐associated HCC.

In conclusion, enhancing immune responses is essential to inhibit hepatitis virus replication and promote tumor cell regression. ICIs targeting PD‐1, PD‐L1, and CTLA‐4 can relieve hepatitis virus‐induced inhibition of T‐cells, restoring their activation and cytotoxic function, and serving as a systemic treatment option for HCC alongside targeted therapies. However, low response rates limit their clinical application. CAR‐T therapy modifies T cells to target specific tumor antigens and induce a strong T cell response, enhancing the liver immune microenvironment. Therapeutic vaccines aim to elicit a robust T‐cell response that enhances the liver's immune microenvironment and inhibits the progression from viral hepatitis to HCC. Additionally, OV therapy can selectively replicate in virus‐infected or tumor cells, sparing healthy cells, thereby enhancing immunotherapies efficacy and inhibiting the progression of viral hepatitis to HCC.

### 5.4 Gene‐Based Approaches

Elucidating the specific molecular signatures involved in viral hepatitis‐associated HCC may facilitate their utilization in diagnostic, therapeutic, and prognostic assessment of HCC linked to HBV or HCV infections. Gene‐based approaches present innovative avenues for the management of HCC by selectively targeting these molecular markers. The modulation of miRNAs, lncRNAs, and m6A modifications, in conjunction with the clustered regularly interspaced short palindromic repeats/CRISPR‐associated system 9 (CRISPR/Cas9) genome‐editing system, can directly manipulate oncosuppressive or oncogenic molecules. This precision medicine approach has the potential to enhance therapeutic efficacy, minimize adverse effects, and pave the way for personalized treatment regimens tailored to individual patients.

#### 5.4.1 MicroRNAs

MiRNAs have emerged as promising, novel, noninvasive biomarkers for the early diagnosis, prognosis, and evaluation of HCC. In HCV patients, serum levels of miRNA‐122 expression are elevated compared with controls but decreases in chronic cirrhosis and HCC, suggesting its potential as a biomarker for diagnosing the progression of HCV infection [609]. Detection of specific miRNA expression has also revealed that miR‐210‐3p is uniquely upregulated in HBV‐related HCC, enhancing HBx expression [610]. Consequently, miRNA‐122 and miR‐210‐3p may serve as a valuable biomarker of HBV‐related HCC, and their inhibition could prevent hepatocarcinogenesis associated with HBV. Additionally, a study examining patients with HCV‐related fibrosis, cirrhosis and HCC has demonstrated that miR‐484 and miR‐524 expression offers promising diagnostic potential: miR‐484 could differentiate late‐stage fibrosis from mild fibrosis and HCC, while miR‐524 could distinguish cirrhosis from fibrosis, suggesting that miRNAs can serve as sensitive biomarkers for staging HCV‐related liver disease progression [611].

MiRNA‐based therapies for HCC provide a dual therapeutic strategy: miRNA replacement to restore tumor‐suppressive miRNAs, thereby inhibiting tumor growth, and miRNA antagonism to target oncogenic miRNAs and mitigate their adverse effects. One promising example is RG101, a GalNAc‐conjugated anti‐miR‐122 oligonucleotide, which has shown promise in reducing viral load in chronic HCV patients [612]. Combining miRNA replacement and antagonism therapies can amplify their benefits for HCC. For instance, cotreatment with a miR‐122 mimic and miR‐221 inhibitor inhibits cancer cell proliferation and angiogenesis while promoting apoptosis and necrosis by targeting specific peptidase 1 (SENP1) and ADP‐ribosylation factor 4 (ARF4) genes, respectively [613]. Innovative delivery systems have also been developed to enhance the therapeutic potential of miRNAs in HCC. A synthetic miRNA‐based targeted therapy using ultrasound‐targeted microbubble destruction (UTMD) to locally deliver miRNA‐loaded nanoparticles has been developed for HCC treatment [614]. This UTMD‐mediated delivery of miRNA‐122 and anti‐miRNA‐21 successfully modulates the tumor immune microenvironment at the cytokine level. Another approach involves encapsulating four miRNAs, including miRNA‐122, miRNA‐100, anti‐miRNA‐21, and anti‐miRNA‐10b, within biodegradable PLGA–PEG nanoparticles, and delivering them to HCC models. This significantly enhances the effectiveness of doxorubicin chemotherapies in HCC, achieving marked tumor growth inhibition and an increased apoptotic index [615].

#### 5.4.2 Long Noncoding RNAs

LncRNAs have presented promising avenues for HCC precision medicine by acting as potential biomarkers, prognostic indicators, and therapeutic targets. HOX antisense intergenic RNA (HOTAIR) is significantly upregulated in HBV‐infected cells and peripheral blood mononuclear cells (PBMCs) from CHB patients, making it a potential diagnostic and therapeutic biomarker for HBV [165]. The combined serum expression profiling of HULC, UCA1, and HOTAIR lncRNAs shows strong diagnostic performance in distinguishing HCC from liver diseases, including chronic HBV infection, fatty liver and cirrhosis, with potential implications for early diagnosis and personalized HCC treatment [616]. In HCC patients, contrast‐enhanced ultrasound (CEUS) grading positively correlates with metastasis‐associated lung adenocarcinoma transcript 1 (MALAT1) expression [617]. MALAT1 is also heavily involved in HBx‐related HCC progression, and silencing MALAT1 can block HBx‐induced CSCs generation, stemness‐related factor activation, and tumorigenicity via the PI3K/AKT signaling [147].

Targeting lncRNAs involved in tumor progression presents a promising strategy for virus‐related HCC. For instance, metformin shows an inhibitory effect on HBV‐associated HCC by suppressing HBx‐induced HULC overexpression [618]. Gallic acid has been observed to reduce MALAT1 expression, thereby inhibiting MALAT1‐mediated Wnt/β‐catenin signaling and suppressing tumorigenesis [619]. Furthermore, an endosomal pH‐responsive nanoparticle platform designed for codelivery of siRNA and a cisplatin prodrug specifically transports siRNA into the nucleus, reversing cisplatin resistance by silencing nuclear‐localized MALAT1 and inhibiting HCC tumor growth [620].

#### 5.4.3 M6A Modifications

M6A modifications hold promise as diagnostic, therapeutic, and prognostic tools in HBV/HCV‐associated HCC. ALKBH5 exhibits elevated expression in HBV–HCC tissues, moderate expression in paracancerous tissues, and diminished expression in normal liver tissues, suggesting its potential as a biomarker for diagnosing the progression of HBV‐related HCC [621]. Additionally, m6A modifications serve as prognostic markers for HCC, with METTL3, YTHDF2, and YTHDF1 highly expressed in high‐risk HCC groups, while zinc finger CCCH‐type containing 13 (ZC3H13) is highly expressed in low‐risk groups [622]. Additionally, m6A‐modified lncRNAs may predict OS in HCC patients by revealing differences in tumor‐infiltrating immune cells, immune checkpoint expression, and chemotherapy sensitivity, providing new insights into HCC prognosis, immunotherapies and chemotherapies [623].

Given the role of m6A dysregulation in the pathophysiology of viral hepatitis‐associated HCC, it represents a promising therapeutic target. ISG20, a 3′–5′ exonuclease enzyme, binds to m6A‐containing HBV transcripts through YTHDF2, thereby promoting HBV RNA degradation, inhibiting HBV replication, and potentially preventing HBV‐driven hepatocarcinogenesis [624]. Another m6A reader YTHDF1 has been shown to promote HCC progression through the YTHDF1–m6A–NOTCH1 epigenetic axis and is a viable therapeutic target for HCC. Lipid nanoparticles targeting YTHDF1 significantly enhance the efficacy of lenvatinib and sorafenib in HCC models [625]. Additionally, a mannose‐modified antigen‐capturing nanoplatform has been developed to codeliver TAAs and an m6A demethylase inhibitor to tumor‐infiltrating DCs. In vivo study has demonstrated that the nanoplatform promotes DCs maturation and enhances effector T cell tumor infiltration, which synergizes with ICIs to inhibit distant tumor growth and lung metastasis [626]. Research on targeting m6A is still in the early stages, with limited therapy available, and further validation of their effectiveness is needed in preclinical and clinical studies.

#### 5.4.4 CRISPR/Cas9

The CRISPR/Cas9 is originally a self‐defense mechanism in prokaryotes and an adaptive immune system in bacteria to resist foreign genetic material, has evolved into a powerful gene‐editing tool. Characterized by its simplicity, efficiency, low cost, and versatility, CRISPR/Cas9 opens new avenues for treating viral hepatitis‐induced HCC [627]. On one hand, CRISPR/Cas9 enables targeted disruption of specific genomic regions in infectious agents or modulation of cellular factors implicated in viral replication through RNA‐guided double‐strand DNA breaks [628]. On the other hand, it enables rapid and precise gene editing. Integrating CRISPR/Cas9 with other therapeutic approaches may further inhibit tumor growth and enhance survival rates in patients with HBV/HCV‐associated HCC [627].

As previously noted, the HBx is a momentous mediator in HBV‐induced HCC pathogenesis by promoting EMT, making it an attractive target for CRISPR/Cas9. A novel HBx‐specific single‐guide RNA (sgRNA) designed with CRISPR/Cas9, termed HBx–CRISPR, has been found to reduce cccDNA levels and HBsAg production in HBV–HCC cells [629]. However, the genetic diversity of the HBV genome limits the applicability of genome editing across all patients. To counteract this, adenovirus vectors expressing multiplex guide RNAs for CRISPR/Cas9 allow simultaneous gene knockouts, addressing target heterogeneity and preventing escape mutants in genome‐editing therapy [630]. Additionally, disruption of cyclophilin D with CRISPR/Cas9 impairs mitochondrial membrane integrity, reduces viral replication, and protects against HCV‐induced inflammation, cell damage, and the heightened HCC risk [631].

Despite the promise of CRISPR/Cas9, challenges such as off‐target effects, off‐cell activity, and immunotoxicity should be addressed. To enhance precision and safety, a nanocomplex delivery system enables efficient coloading of gene‐drug combinations, allowing for accurate gene editing and synergistic tumor inhibition without affecting normal tissues [632]. For instance, a polyamidoamine‐aptamer‐coated hollow mesoporous silica nanoparticle codelivering sorafenib and CRISPR/Cas9 enables efficient EGFR gene therapies and tumor inhibition without harming major organs [633]. Growth differentiation factor 15 (GDF15), a cytokine elevated in HCC patients following DAA treatment, promotes immunosuppression in the tumor microenvironment [634]. Targeted delivery of CRISPR/Cas9 against GDF15 using nanocapsules coated with HCC‐specific SP94 peptides, enhances immune cell infiltration in HCC and promotes favorable immune microenvironment changes, as evidenced by cytometry by time‐of‐flight (CyTOF) analysis [635]. Additionally, a biomimetic delivery nanoplatform has been designed for HCC treatment by self‐assembling a PD‐L1‐targeted CRISPR/Cas9 system with ursolic acid (UA). UA activates the natural immune system through the TLR‐2‐myeloid differentiation primary response 88 (MyD88)–TRAF6 pathway, while PD‐L1 blockade promotes cytotoxic T cell infiltration, leading to significant tumor regression by simultaneously engaging innate and adaptive immunity [636].

These gene‐targeted strategies highlight the potential to regulate multiple dysregulated genes and pathways in HCC, offering a more holistic approach compared with single‐target drugs. However, as most of these studies remain in preclinical stages, further clinical trials are essential to confirm the efficacy and safety of these therapies in patients.

### 5.5 Innovative Treatments

In addition to the approaches mentioned for preventing the malignant transformation of viral hepatitis to HCC, new intervention pathways, such as gut microbiota regulation and artificial intelligence (AI) intervention, hold promise for future clinical applications to improve therapeutic efficacy.

#### 5.5.1 Gut Microbiota

Gut microbiota regulation can repair the intestinal barrier and enhance the antiviral immune defense in the liver, thus inhibiting viral hepatitis‐induced HCC [637]. Certain agents with anticancer properties have been identified to modulate the gut microbiota, thereby improving the tumor immune microenvironment. For instance, stigmasterol has been shown to modulate diversity of the intestinal microbiota and significantly enhance populations of *Lactobacillus johnsonii, Lactobacillus murinus*, and *Lactobacillus reuteri*, leading to a reduced Treg/CD8^+^ T cell ratio, which enhances immune responses within the tumor microenvironment [638]. Additionally, intervention with Echinacea purpurea polysaccharide in HCC promotes expression of intestinal tight junction proteins, thereby repairing the intestinal barrier, which aids in controlling LPS leakage, subsequently inhibiting the TLR4/NF‐κB pathway and reducing inflammatory factor expression [639]. A novel “Trojan‐horse” strategy has been proposed, using an oral dextran–carbenoxolone (DEX–CBX) conjugate to combine prebiotics with glycyrrhetinic acid (GA) for targeted GA delivery to HCC via the gut–liver axis [640]. DEX–CBX significantly increases the abundance of *Akkermansia*, a bacterium known to strengthen systemic immune responses, which enhances NK T cells, CD8^+^ T cells, and M2 macrophages, offering a novel strategy to precisely modulate hepatic inflammation and gut microbiota in HCC.

Probiotics, live organisms that support host health, may also benefit HCC patients by improving the intestinal microenvironment [641]. *Lactobacillus brevis SR52‐2* and *Lactobacillus delbrueckii Q80* have exhibited anti‐HBV properties by inhibiting HBeAg and HBsAg expression and supporting gastrointestinal health in HBV‐associated HCC patients [642]. Moreover, modulating the gut microbiota has further been shown to enhance the efficacy of anticancer drugs. In a study of advanced HCC patients treated with sorafenib, *Enterococcus faecium* (*Efm*) was significantly enriched in those who responded to treatment compared with nonresponders, suggesting that *Efm* could enhance sorafenib's therapeutic effects [643]. Additional in vivo study has shown that *Efm* colonization induces IL‐12 and IFN‐γ production and increases the proportion of IFN‐γ and CD8^+^ T cells in the tumor microenvironment. Notably, butyrate, a SCFA produced by gut bacteria from dietary fiber and probiotics, possesses multiple anticancer mechanisms, including epigenetic regulation, immune modulation and metastasis suppression [644]. Che et al. developed nanoparticles coencapsulating butyrate and sorafenib, coated with an anti‐GPC3 antibody, which prolonged drug retention time and demonstrated efficient, safe targeting to HCC cells [645].

Fecal microbiota transplantation (FMT), which transfers functional gut bacteria from a healthy donor to the patient, is a novel approach to restore gut microbiota balance and has shown promise in treating CLDs [646]. In a case‐controlled, open‐label pilot trial, FMT successfully cleared HBeAg in patients with persistent HBeAg positivity after long‐term antiviral therapy [647]. In chronic HBV infection, oral capsule‐based intestinal microbiota transplantation (IMT) decreased HBsAg and total bile acid (TBA) levels and restored gut microbiota abundance [648]. As a unique biological therapy derived from the human body and not classified as an organ transplant, FMT requires stringent monitoring and preservation of donor specimens for broader application.

#### 5.5.2 Artificial Intelligence

Due to delayed diagnoses and the suboptimal efficacy of current therapeutic interventions, the prognosis for HCC remains poor. Recently, AI has risen as a distinctive opportunity to enhance the entire spectrum of HCC clinical management, encompassing risk prediction, diagnosis, and prognostication [649]. A gradient‐boosting machine (GBM)‐based model has been developed to generate risk scores for predicting HCC development in a cohort of 13,508 patients with CHB treated with ETV or tenofovir, which has demonstrated superior performance in risk stratification, identifying a subgroup of patients at minimal risk of developing HCC who could benefit from less intensive surveillance [650]. Using data from 5701 observations of 1985 HCC patients at a single center from 2000 to 2021, an AI‐based system, PRAID, was constructed and evaluated for its prognostic value, specifically in predicting 1‐ and 3‐year survival. Results have demonstrated that PRAID provided valuable prognostic information for short‐ and medium‐term survival, aiding in therapeutic decision‐making for both initial and recurrent HCC cases [651].

Machine learning (ML), a subtype of AI, enables computer programs to “learn” from data and improve through experience. Evidence suggests that ML offers a more accurate risk stratification model for HCC development after SVR compared with conventional methods [649]. Minami et al. developed a novel prediction model, the SMART model, which uses ML algorithms to predict HCC occurrence following HCV eradication. The SMART model incorporates seven accessible parameters: age, platelet count, serum AFP, gamma‐glutamyl transferase (GGT), albumin, AST levels, and body mass index (BMI), allowing for the generation of individualized predictive curves, thereby supporting personalized and cost‐effective surveillance after SVR [652]. Additionally, the SMART‐HCC score, an ML‐based HCC risk score incorporating LS measurements, identified LS as one of the top clinical predictors. The SMART‐HCC score has been trained to predict HCC in 5,155 adult patients with various CLDs in Korea and subsequently validated in two prospective cohorts from Hong Kong (*N* = 2732) and Europe (*N* = 2384), which holds promise for clinicians in stratifying HCC risk among CLD patients [653]. Deep learning (DL) is a more advanced subtype of ML that utilizes multilayered neural networks to process large datasets. When applied to digital histopathologic images, DL can generate recurrence risk scores, potentially enhancing current stratification methods and refining the clinical management of patients undergoing primary surgical resection for HCC [654]. Multi‐DL, a neural network comprising encoder, prediction, and segmentation pathways, has been developed using contrast‐enhanced computed tomography (CT) images, effectively stratifying patients into low‐ and high‐risk groups with significant survival differences, which offers clinicians valuable insights for tailoring therapeutic regimens [655].

AI is expected to significantly transform the treatment and care of patients with virus‐related HCC. Although significant advancements have been made over the past decade, further refinement in risk prediction, diagnosis, and prognostication remains critical. Implementing these technologies into clinical practice presents several challenges, including the need for standardized data collection, sharing, and storage frameworks, as well as further validation to confirm the reliability and robustness of these models.

In conclusion, innovative therapeutic approaches have provided more precise treatment options for HBV/HCV‐associated HCC. Gut microbiota‐targeted treatments, tailored to the unique intestinal microenvironment of each individual, can repair the intestinal barrier, enhance liver antiviral immunity, and inhibit HCC induced by viral hepatitis. Additionally, AI can help predict disease progression, personalize treatment plans, improve outcomes, and reduce side effects through the analysis of large medical datasets. These strategies offer multiple treatment options to inhibit the progression of viral hepatitis to HCC, with the potential to improve outcomes and patient survival rates.

## 6 Conclusion and Prospects

The malignant transformation of chronic viral hepatitis into HCC represents a complex interplay of viral, host, and environmental factors that culminate in a significant health burden globally. Both HBV and HCV play pivotal roles in the development of HCC through various oncogenic mechanisms, including viral integration and genome instability, epigenetic alterations, oxidative stress, disruption of gut microbiota, chronic inflammation and immune evasion, and activation of oncogenic pathways, further underscoring the importance of viral persistence as a key driver of hepatocarcinogenesis. Chronic infection creates an inflammatory microenvironment marked by the influx of immune cells, leading to a cycle of tissue damage and repair. This environment is conducive to cellular transformation, highlighting the critical role of host responses in the development of HCC. Moreover, the dysregulation of critical signaling pathways and the accumulation of genetic and epigenetic alterations further promote oncogenesis. Additionally, the presence of additional risk factors, such as alcohol consumption, obesity, and metabolic syndrome, synergistically contributes to the progression from chronic hepatitis to HCC. Understanding these cofactors is vital for risk stratification and the development of targeted interventions.

Current antiviral therapy has demonstrated efficacy in reducing HCC incidence among patients with chronic hepatitis. However, those with severe liver fibrosis and cirrhosis remain at high risk for HCC development. Notably, the rise of gene and immunotherapies offers new avenues to improve treatment outcomes. ICIs reduce immune suppression and boost the immune response to eliminate tumor cells, providing a systemic treatment option for HCC in conjunction with targeted therapies. However, their clinical application is limited by low response rates. CAR‐T therapy modifies T cells to target specific tumor antigens and induces a strong T cell response, enhancing the liver immune microenvironment. Additionally, genetically engineered viruses employed in OV therapy can selectively replicate within virus‐infected or tumor cells, sparing healthy cells, thereby enhancing immunotherapies efficacy and inhibiting the progression of viral hepatitis to HCC. With the discovery of additional molecules involved in HCC development, targeted strategies such as miRNAs, lncRNAs, m6A modifications, and CRISPR/Cas9 technology can directly target those molecules that either inhibit or promote malignant transformation, leading to more precise treatment options. Furthermore, gut microbiota‐targeted treatments repair the intestinal barrier, enhance liver antiviral immunity, and inhibit HCC induced by viral hepatitis. Additionally, AI aids in predicting disease progression, personalizing treatment plans, improving outcomes, and minimizing side effects by analyzing extensive medical datasets. Moreover, recognizing patients at high risk for HCC is paramount for effective management. Implementing regular screening protocols for individuals with chronic hepatitis can facilitate early detection, significantly improving prognosis. On the other hand, educating patients about the risks of chronic viral hepatitis and empowering them with knowledge can enhance compliance with screening protocols and treatment regimens, ultimately improving outcomes.

While significant strides have been made in understanding the transition from viral hepatitis to HCC, several critical areas require further investigation. Advanced technologies, such as CRISPR gene editing and single‐cell RNA sequencing, elucidate the dynamics of cellular responses to viral infections, providing deeper insights into HCC pathogenesis. Moreover, the development of new therapeutic strategies that target specific molecular pathways involved in malignant transformation is imperative. Research should focus on identifying and validating new drug targets, optimizing existing therapies, and exploring combination strategies that may enhance treatment efficacy. Considering the global impact of viral hepatitis and HCC, it is essential to promote international collaboration among researchers, healthcare providers, and public health officials. Collaborative research initiatives facilitate the knowledge sharing and development of standardized treatment protocols across diverse populations, ultimately contributing to improved global health outcomes. Furthermore, increasing access to healthcare, improving education and awareness in at‐risk populations, and ensuring equitable access to screening and treatment services are equally crucial for reducing the incidence of virus‐related HCC.

In summary, we review the mechanisms driving the malignant transformation of viral hepatitis into HCC and the clinical applications of various intervention methods, providing a clearer understanding of the transformation process and supporting the exploration and development of new drugs and therapies. Moving forward, it is essential to prioritize research that clarifies the complexities of HCC pathogenesis while also developing effective screening, treatment, and prevention strategies. Integrating these efforts into clinical practice aims to alleviate the HCC burden associated with viral hepatitis, improve patient outcomes, and advance public health initiatives.

## Author Contributions

Huimin Yuan and Ruochen Xu wrote the review and drew the figures. Yonghui Zhang and Ming Xiang reviewed the manuscript. Senlin Li, Mengzhu Zheng, and Qingyi Tong assisted in manuscript and provided some helpful suggestions. The manuscript has been read and approved by all coauthors. All authors contributed to the review and approved the submitted version.

## Ethics Statement

The authors have nothing to report.

## Conflicts of Interest

The authors declare no conflicts of interest.

## Supporting information



Supporting Information

## Data Availability

No applicable.
