# Supplementary material for: The Malignant Transformation of Viral Hepatitis to Hepatocellular Carcinoma: Mechanisms and Interventions
Source: MedComm (2020). 2025 Mar 8;6(3):e70121. doi: 10.1002/mco2.70121 (PMC11890166; doi:10.1002/mco2.70121)
Supplement: Supplementary file 1 — Supporting Information [file MCO2-6-e70121-s001.docx]

**The malignant transformation of viral hepatitis to hepatocellular carcinoma (HCC): mechanisms and interventions**

**Running title:** Malignant transformation and interventions of HCC

Huimin Yuan^1^, Ruochen Xu^1^, Senlin Li^1^, Mengzhu Zheng^1^, Qingyi Tong^1,2^, Ming Xiang^1^ * and Yonghui Zhang^2^ *

^1^ Department of Pharmacology, School of Pharmacy, Tongji Medical College, Huazhong University of Science and Technology, Wuhan, Hubei, 430000, China

^2^ Hubei Key Laboratory of Natural Medicinal Chemistry and Resource Evaluation, School of Pharmacy, Tongji Medical College, Huazhong University of Science and Technology, Wuhan, Hubei, 430000, China

***Corresponding author:**

Yonghui Zhang. Tel.: +86 2783692784, E-mail: zhangyh@tjmu.edu.cn

Ming Xiang. Tel.: +86 2783692739, E-mail: xiangming@tjmu.edu.cn

**Supplementary Figures**


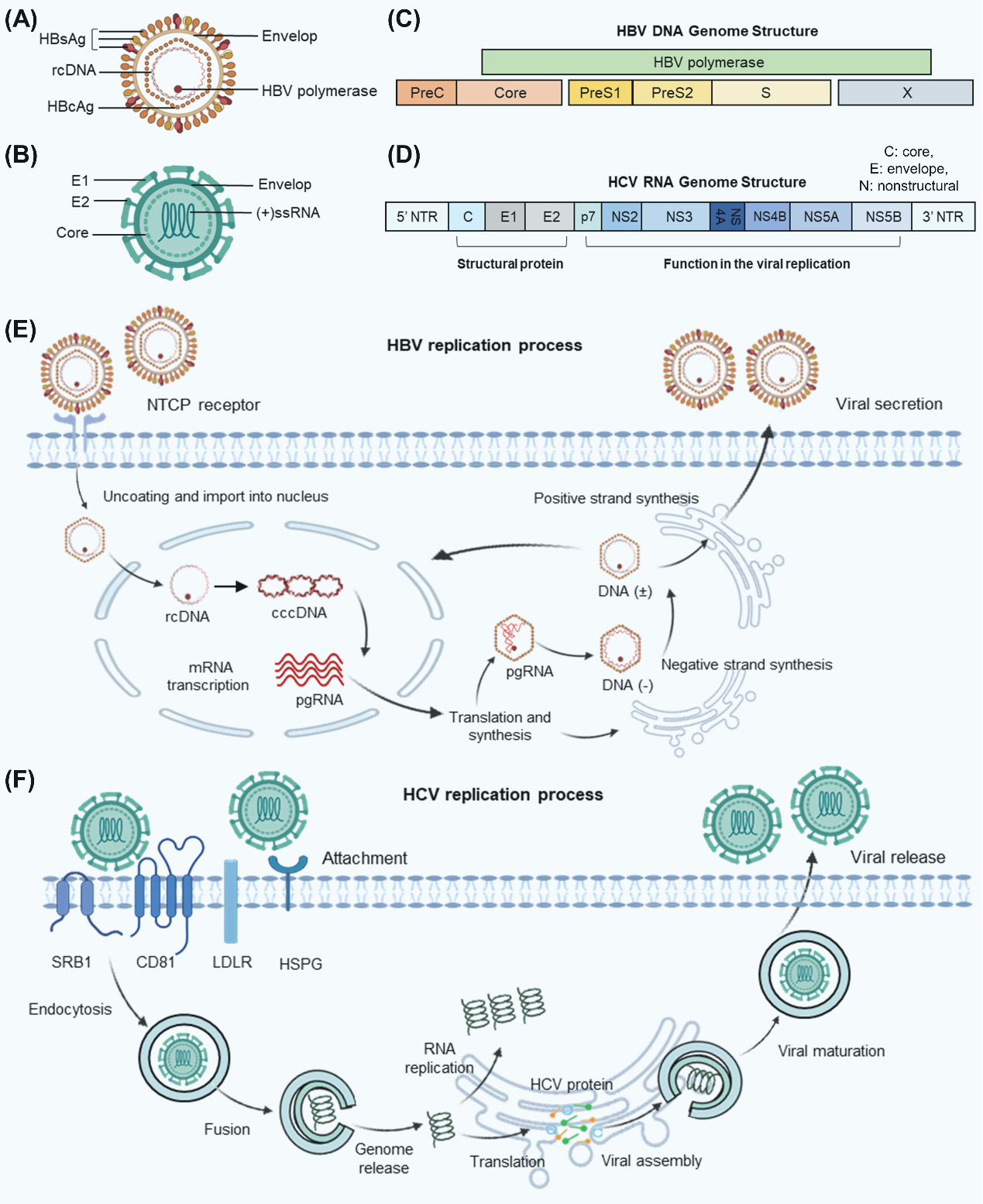


**Figure S1. Overview of viral structure and lifecycle.** (A) Structure of HBV. (B) Structure of HCV. (C) Genomic structure of HBV DNA. (D) Genomic structure of HCV RNA. (E) HBV replication in host cells. (F) HCV replication in host cells. cccDNA, covalently closed circular DNA; HBV, hepatitis B virus; HCV, hepatitis C virus; HSPG, heparan sulfate proteoglycan; LDLR, low density lipoprotein receptor; NTCP, taurocholic acid sodium cotransporter polypeptide 1; pgRNA, pregenomic RNA; rcDNA, relaxed circular DNA; SRB1, scavenger receptor class B1.
